# Rodent Models of Invasive Aspergillosis due to *Aspergillus fumigatus*: Still a Long Path toward Standardization

**DOI:** 10.3389/fmicb.2017.00841

**Published:** 2017-05-16

**Authors:** Guillaume Desoubeaux, Carolyn Cray

**Affiliations:** ^1^Division of Comparative Pathology, Department of Pathology and Laboratory Medicine, Miller School of Medicine, University of MiamiMiami, FL, USA; ^2^Service de Parasitologie—Mycologie—Médecine tropicale, Centre Hospitalier Universitaire de ToursTours, France; ^3^Centre d'Etude des Pathologies Respiratoires (CEPR) Institut National de la Santé et de la Recherche Médicale U1100/Équipe 3, Université François-RabelaisTours, France

**Keywords:** invasive aspergillosis, *Aspergillus fumigatus*, rodent models, mice, experimental infection

## Abstract

Invasive aspergillosis has been studied in laboratory by the means of plethora of distinct animal models. They were developed to address pathophysiology, therapy, diagnosis, or miscellaneous other concerns associated. However, there are great discrepancies regarding all the experimental variables of animal models, and a thorough focus on them is needed. This systematic review completed a comprehensive bibliographic analysis specifically-based on the technical features of rodent models infected with *Aspergillus fumigatus*. Out the 800 articles reviewed, it was shown that mice remained the preferred model (85.8% of the referenced reports), above rats (10.8%), and guinea pigs (3.8%). Three quarters of the models involved immunocompromised status, mainly by steroids (44.4%) and/or alkylating drugs (42.9%), but only 27.7% were reported to receive antibiotic prophylaxis to prevent from bacterial infection. Injection of spores (30.0%) and inhalation/deposition into respiratory airways (66.9%) were the most used routes for experimental inoculation. Overall, more than 230 distinct *A. fumigatus* strains were used in models. Of all the published studies, 18.4% did not mention usage of any diagnostic tool, like histopathology or mycological culture, to control correct implementation of the disease and to measure outcome. In light of these findings, a consensus discussion should be engaged to establish a minimum standardization, although this may not be consistently suitable for addressing all the specific aspects of invasive aspergillosis.

## Introduction

Aspergillosis is an airborne fungal infection due to ubiquitous molds belonging to the genus *Aspergillus*. In human medicine, *Aspergillus fumigatus* is the main species involved in aspergillosis with isolation in more than 80% of the clinical samples with positive culture, regardless the context (Desoubeaux et al., 2014a). *A. fumigatus* is present in the environment, especially in air, water, plants, and soil. When its spores are inhaled, it may be responsible for a wide-range of distinct clinical entities, but invasive aspergillosis—which is primarily reported in immunocompromised individuals—remains the most feared because of its high mortality rates ranging from 30 to 100% (Lortholary et al., 2011; Bitar et al., 2014).

As both basic and clinical knowledge about invasive aspergillosis is limited, laboratory models of the disease are needed. In spite of recent major advances (Sable et al., 2008; Brown, 2011; Steele and Wormley, 2012; Wüthrich et al., 2012; Drew et al., 2013; Lanternier et al., 2013), there are still many concerns to be addressed: for example, why a particular strain is more virulent than another (Becker et al., 2006)? How to prevent a contamination? Which route of drug administration to be privileged to cure the infection (Becker et al., 2002b)? And why a diagnostic tool is better than another one in such context (Becker et al., 2000, 2002a)? All these *scenarii* are very complex and for such purposes, development of animal models seem more valuable research tools than *in vitro* experiments, especially because they span the gap between the bench and the clinic bed. Theoretically, animal models mimic, as closely as possible, the clinical course and the symptoms of the disease as observed in human patients. Also they are assumed to be more easily repeatable, less expensive, and potentially more readily and quickly provide reliable scientific responses than clinical trials. Unfortunately for animal models studying invasive aspergillosis (Mahajan et al., 1978; Ghori and Edgar, 1979; Chaudhary and Singh, 1983; Chaudhary et al., 1988; Chilvers et al., 1989; Andriole et al., 1992; Kurtz et al., 1995; Leenders et al., 1996; Richard et al., 1996; Cicogna et al., 1997; Kirkpatrick et al., 2000b; Clemons and Stevens, 2005; Gavaldà et al., 2005; Lewis and Wiederhold, 2005; Patterson, 2005; Chandenier et al., 2009), heterogeneity has always been great regarding their technical variables, like the species or strains to be used, the animal sex and weight, the immunosuppressive regimen, the route of experimental infection, the fungal inoculum size, and the methods to assess fungal burden (Hohl, 2014). Thus, it currently does not exist any consensus for a unique animal model. However, one can notice that rodents have been mostly used so far, because they are of small size, inexpensive, easy-to-handle, and the ready availability of reagents and methods (Andriole et al., 1992; Clemons and Stevens, 2005; Lewis and Wiederhold, 2005; Patterson, 2005; Paulussen et al., 2014).

Therefore, it is now critical for animal models to be well-defined (Clemons and Stevens, 2005), and efforts to choose the best one(s) are required before a possible standardization. For such a purpose, we decided to complete a comprehensive overview of all the published reports that dealt with models of invasive aspergillosis. Within the text, and in order to perform a personal criticizing analysis, we sometimes subjectively placed emphasis on some studies that were thought to be interesting for providing specific and relevant information. To circumvent confounding bias, we restricted our study to *A. fumigatus* infection in rodent species. We took this opportunity to address most of the current pending issues. They applied to harmonization of the technical features and experimental settings, and to the following questions: what these assays are used for, how the results derived from them should be interpreted, and what philosophy or ethics should be considered.

## Materials and methods

### Search criteria

**Figure 1:**
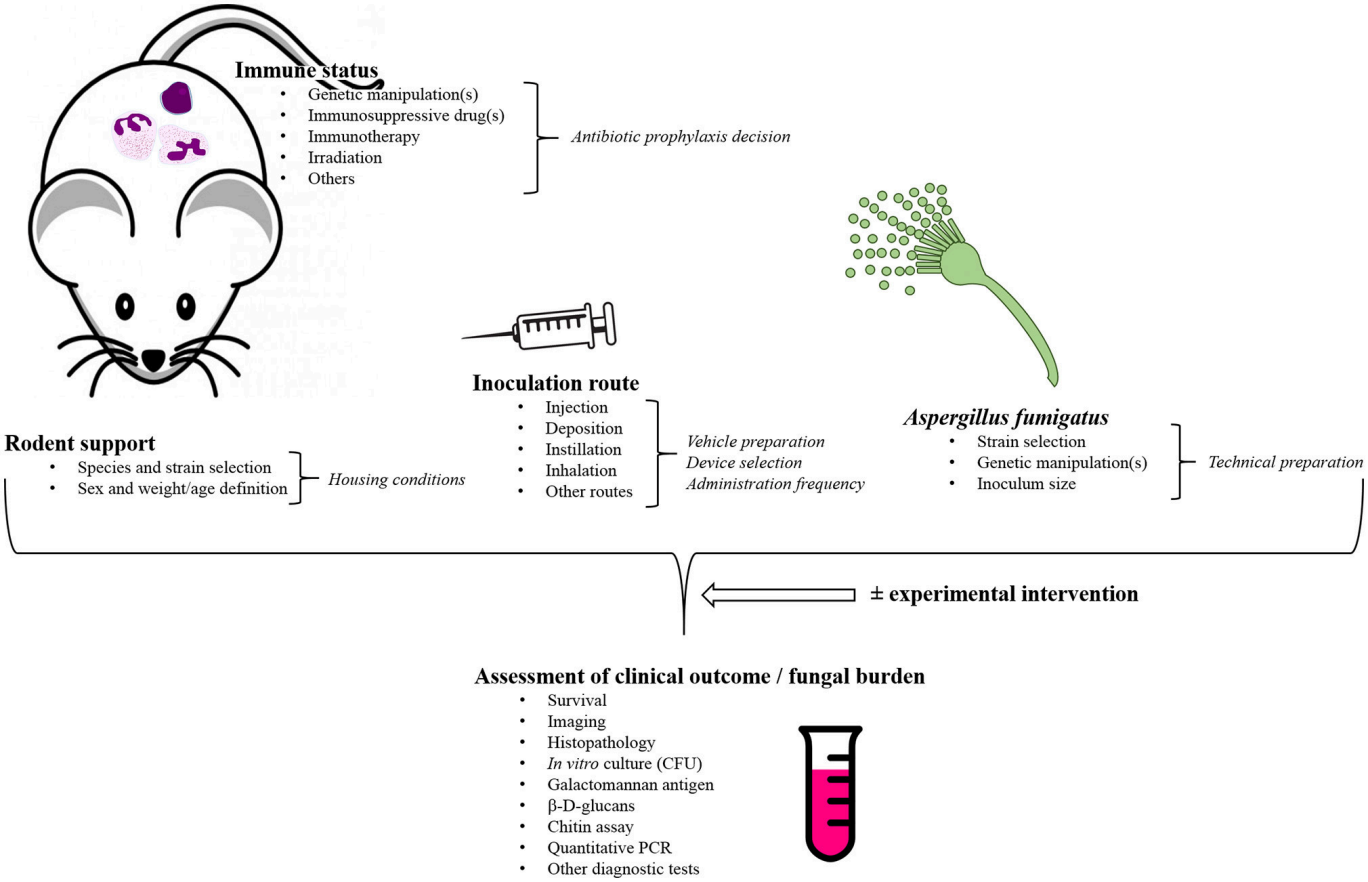
**General overview of the pivotal study parameters that require to be considered when developing a rodent model of invasive aspergillosis caused by ***Aspergillus fumigatus*****. This scheme is probably not exhaustive, but it outlines the major variables that are mostly thought to be critical for model development in laboratories. CFU, Colony-forming unit(s); PCR, Polymerase chain reaction.

A systematic literature review was performed using a rigorous search strategy in the PubMed database for English language literature published up to October 2016, based on the following MeSH terms: [(“*Aspergillus fumigatus*”[Mesh]) OR (((“Aspergillosis”[Mesh]) OR (“Invasive Pulmonary Aspergillosis”[Mesh]) OR (“Pulmonary Aspergillosis”[Mesh])) NOT (“Aspergillosis, Allergic Bronchopulmonary”[Mesh])) AND ((“Models, Animal”[Mesh]) OR ((“Rodentia”[Mesh]) OR (“Mice”[Mesh]) OR (“Rats”[Mesh]) OR (“Guinea Pigs”[Mesh])) OR (“Cricetinae”[Mesh])) NOT (“Rabbits”[Mesh]) NOT (“Birds”[Mesh])]. Then, the authors exhaustively reviewed the retained articles. For each, they thoroughly focused on the pivotal experimental parameters and the major technical features that are assumed to likely influence the results (Schmidt, 2002; Clemons and Stevens, 2005; Lewis and Wiederhold, 2005; Patterson, 2005; Capilla et al., 2007): rodent species and strains as well as their weight and sex, the immunosuppressive regimen they underwent, the *A. fumigatus* strain(s) and the fungal inoculum used for the experimental challenge, the route of inoculation, the clinical, and biological parameters to follow up to assert correct implementation of the disease and its monitoring (Figure 1).

### Analysis

Statistical analyses were performed using XLStat v.2014.6.04® software (Addinsoft, Paris, France). The α-risk was adjusted at 0.05.

## Results

### Number of publications and addressed topics

**Figure 2:**
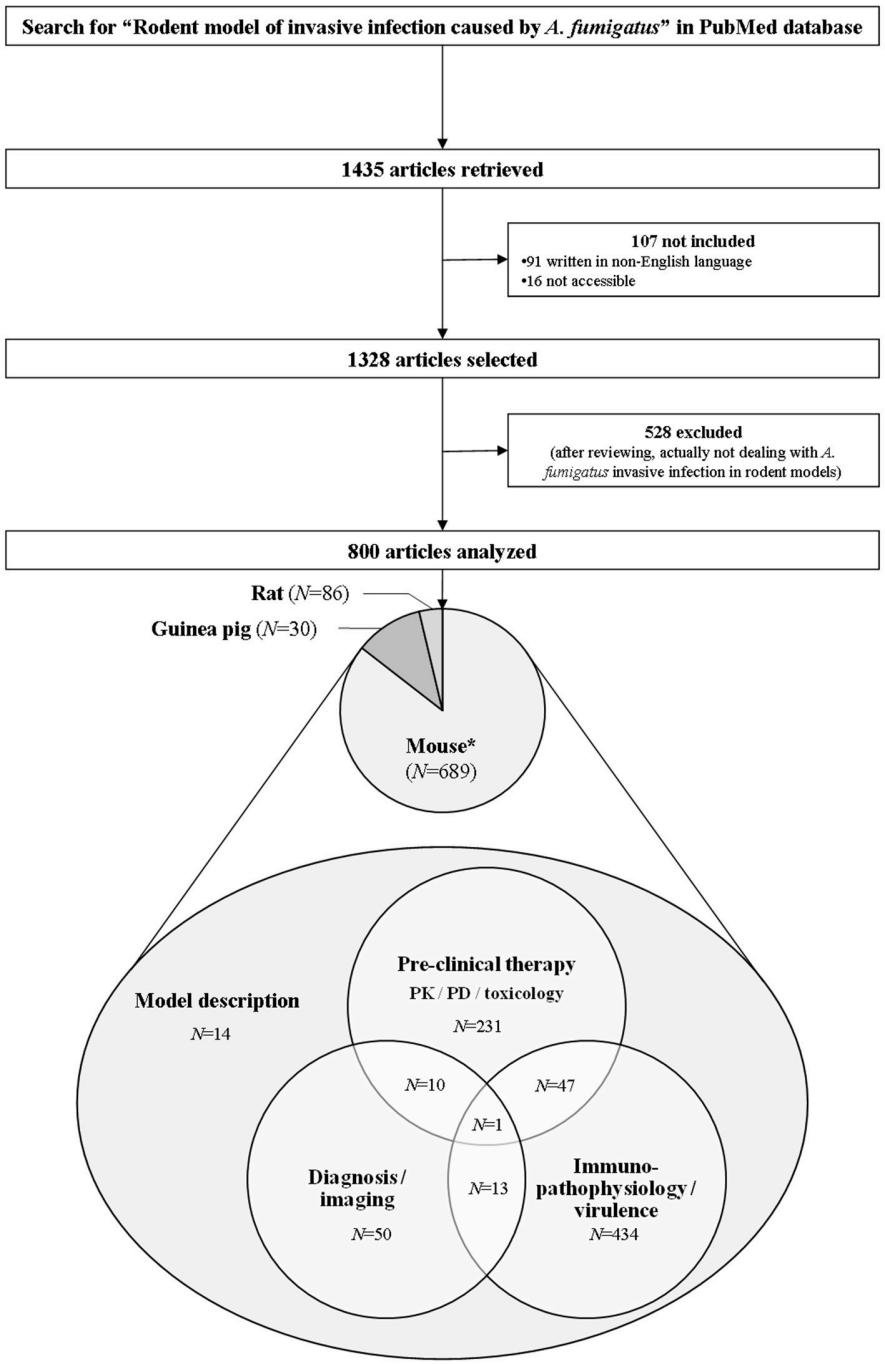
**Flow chart of the bibliometric study**. The research was completed in PubMed up to October 2016 according to the criteria reported in the Section Materials and Methods. Scientific reports, oral communications, and posters were not addressed in this study. *N*, Number; PD, Pharmacodynamics; PK, Pharmacokinetics. ^*^Five articles reported the use of several rodent species at a time: two papers with mice plus rats simultaneously, and three with mice plus guinea pigs.

**Figure 3:**
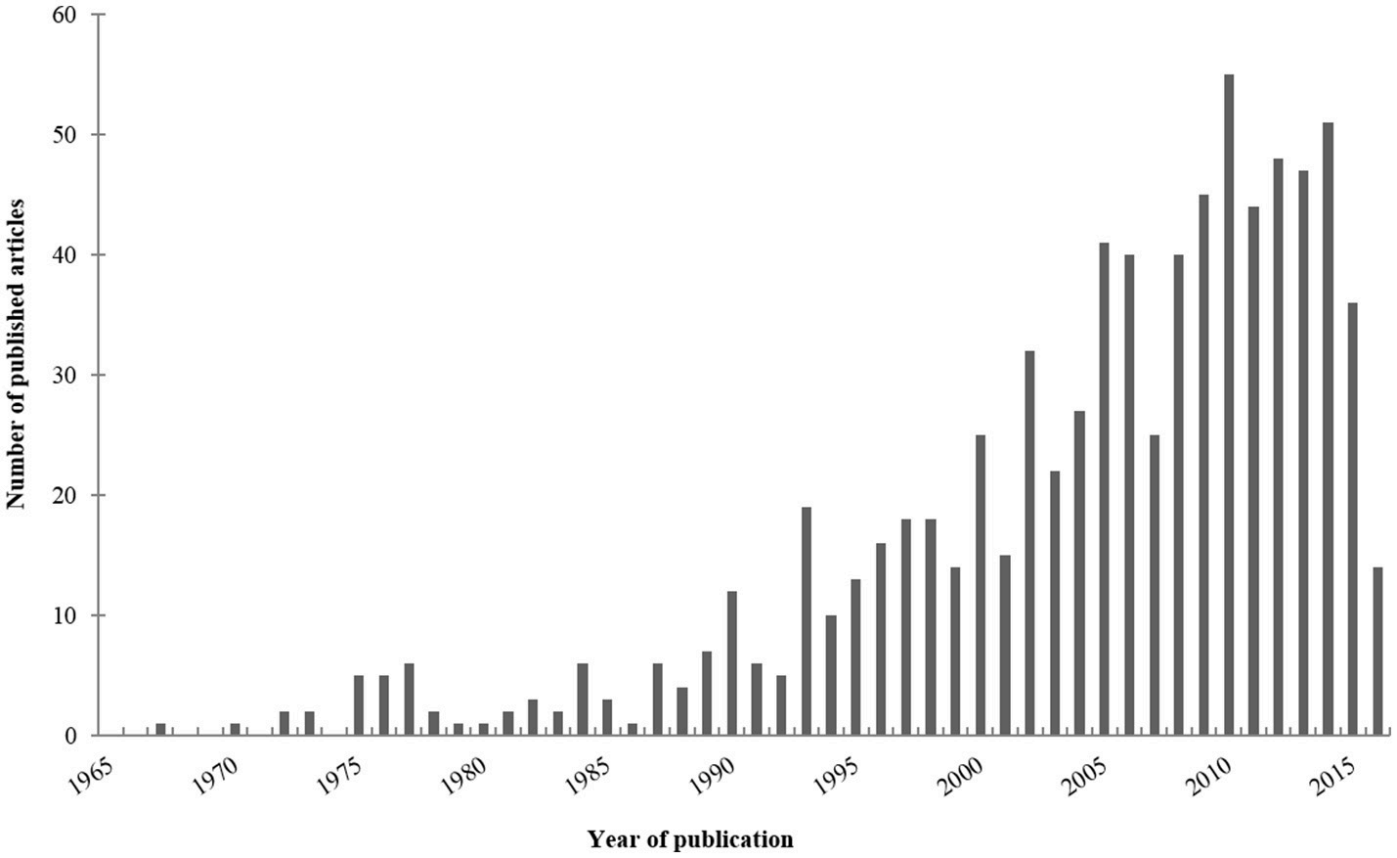
**Published articles ***per*** year**. For this bar chart, have been only taken into account the articles about rodent models of invasive aspergillosis due to *Aspergillus fumigatus* retrieved in PubMed up to October 2016, according to the criteria reported in the Section Material and Methods.

Our electronic search about rodent models of invasive aspergillosis due to *A. fumigatus* retrieved 1,435 publications. Out of them, 91 were excluded since not written in English. Sixteen were not included because not accessible. After thorough reviewing, a total of 800 articles were finally retained for complete analysis (Figure 2). The first paper about rodent model of aspergillosis was published in 1967 (Ford and Friedman, 1967). More than three quarters have been written after the year 2000 (Figure 3).

Key objectives of rodent models of invasive aspergillosis and their major topics are summarized in Table 1. A large majority of all the analyzed articles were dedicated to immunopathology of invasive aspergillosis and study of the fungal virulence, e.g., disease transmission, innate and acquired host-response, genes and proteins involved in fungal invasion, susceptibility to infection: 61.9% addressed this topic vs. 36.1 and 9.3% which were rather devoted to pre-clinical therapy [pharmacology/pharmacokinetics/toxicology, and also several vaccine assays (Clemons et al., 2014) and role of surgery (Habicht et al., 2002)], and to diagnosis/imaging approaches (Yang et al., 2009), respectively. Of note, the analyzed articles frequently covered several research fields at a time: for instance, 10 manuscripts dealt with both diagnosis/imaging and pre-clinical therapy simultaneously, 13 overlapped both diagnosis/imaging and immune-pathophysiology/virulence, and 47 addressed therapeutic and immuno-pathophysiology/virulence concerns within the same studies (Supplementary Material 1). Only a few were purely descriptive, and were thus intended to describe a new model of aspergillosis but with no mention of neither therapeutic assays associated, nor assessment of diagnostic/imaging tests nor immune-pathophysiological studies (Walzl et al., 1987; Dixon et al., 1989; Jensen and Hau, 1990a,b; Jensen and Schønheyder, 1993; Nawada et al., 1996; Yonezawa et al., 2000; Chiller et al., 2002; Sheppard et al., 2004; Steinbach et al., 2004; Zimmerli et al., 2007; Chandenier et al., 2009; Desoubeaux and Chandenier, 2012; Herbst et al., 2013; Leleu et al., 2013a; Zhang et al., 2013; Alcazar-Fuoli et al., 2015). Some of them reported the benefit of new devices to induce the experimental disease (Steinbach et al., 2004; Chandenier et al., 2009; Desoubeaux and Chandenier, 2012; Leleu et al., 2013a), others were focused on the experimental description of particular aspergillosis forms like cerebral aspergillosis (Chiller et al., 2002; Zimmerli et al., 2007) or invasive fungal rhino-sinusitis (Zhang et al., 2013). A few articles described models that offered the opportunity to study aspergillosis in very specific context, like solid-organ transplantation (Herbst et al., 2013) or during bacterial superinfection (Yonezawa et al., 2000). Interestingly, Jensen et al. addressed the clinical consequences for the fetus when the mouse mother was infected with *A. fumigatus* during pregnancy (Jensen and Hau, 1990a,b).

**Table 1:** **Main general objectives for rodent models of invasive aspergillosis due to ***Aspergillus fumigatus*****.

**✓ Mimicry the human disease in**
➢ Local or disseminated aspergillosis
➢ Time lapse of clinical course
➢ Clinical outcome
**✓ Stability and inter-lab reproducibility**
**✓ Ease of use for**
➢ Animal handling
➢ Animal housing
➢ Animal sampling
**✓ Availability of lab tools and reagents dedicated to**
**✓ Reliable basis and background to address studies focusing on**
➢ Strain virulence and pathogenicity
➢ Pathophysiology
➢ Host immune response
➢ Diagnostic tool(s) assessment
➢ Imaging technology(-ies) assessment
➢ Pharmacodynamics/pharmacokinetics/toxicology
➢ Pre-clinical prophylactic assay(s)
➢ Pre-clinical therapeutic assay(s)
➢ Pre-clinical vaccine assay(s)
**✓ Low costs**
**✓ Respect of ethical guidelines**
**✓ Minimum standardization**

*This above listing is probably not exhaustive, but it outlines the goals that are mostly thought to be critical for rodent support development in laboratories*.

### General description of the various rodent models: species and strains, weight and sex

Overall, high variability was noticed regarding the rodent species (Figure 1, Table 2). Mice were used in 86.1% of the selected works, vs. 10.8 and 3.8% for rats and guinea pigs, respectively. Sometimes two of these species were tested concurrently in the same study (Reichard et al., 1997; Odds et al., 1998; Niwano et al., 1999; Hanazawa et al., 2000; Dufresne et al., 2012), like Odds et al. that reported evaluation of possible correlation between *in vitro* antifungal susceptibilities and treatment outcomes in both mice and guinea pigs (Odds et al., 1998), or Dufresne et al. who established that point-of-care diagnosis-based on urinary galactomannan-(GM)-like antigens detection is feasible in both mice and guinea pigs (Dufresne et al., 2012). To date, hamsters have not been tested as a model of invasive aspergillosis.

**Table 2:** **Overall description of all the published rodent models of invasive aspergillosis due to ***Aspergillus fumigatus*****.

	**Mean (unit** ±**standard deviation) or Number (%), [95% confidence interval]**		
	**Mouse (*N* = 689)**	**Rat (*N* = 86)**	**Guinea pig (*N* = 30)**
Weight (grams)	21.7g (±3.8), [21.4–22.0 g]	218.5 g (±71.1), [201.8–235.2 g]	486.1 g (±36.9), [471.5–500.7 g]
Gender (male sex)	277^Ψ^ (50.2%), [46.0–54.35%]	43^Δ^ (60.6%), [49.2–71.9%]	22 (91.7%), [80.6–100.0%]
**Rodent strains, *including*[Table-fn TN3]:**			
– Outbred	201 (29.2%), [26.0–32.8%]	56 (65.1%), [55.0–75.0%]	20 (83.3%), [68.4–98.2%]
– Inbred	459 (66.6%), [63.5–70.6%]	24 (27.9%), [18.4–37.4%]	4 (16.7%), [1.8–31.6%]
– Hybrid/Congenic	18 (2.5%), [1.3–3.6%]	/	/
**Immunosuppressive regimens, *including*[Table-fn TN3]:**	529 (78.0%), [74.9–81.1%]	67 (78.8%), [70.1–87.5%]	19 (63.3%), [46.1–80.6%]
– Alkylating drug(s)	283 (41.9%), [38.2–45.6%]	44 (51.8%), [41.1–62.4%]	19 (63.3%), [46.1–80.6%]
– Steroid(s)	299 (44.3%), [40.6–48.0%]	43 (51.2%), [40.5–61.9%]	15 (50.0%), [32.1–67.9%]
– Other immunosuppressive drug(s)	30 (4.4%), [2.9–6.0%]	1 (1.2%), [0.0–3.5%]	1 (3.3%), [0.0–9.8%]
– Immunotherapy	44 (6.5%), [4.6–8.4%]	/	/
– Irradiation	21 (3.1%), [1.8–4.4%]	/	/
– Mutation(s)/deletion(s) in rodent genetic background	124 (18.4%), [15.4–21.3%]	/	/
– Alternative method(s)	3 (0.4%), [0.0–0.9%]	1 (1.2%), [0.0–3.5%]	/
***Aspergillus fumigatus* strains^*^*including[Table-fn TN3]:***			
– AF293, ATCC MYA-4609, FA/1153, FGSC A1100, CBS 101355, NCPF 7367, IHEM18963	126 (19.5%), [16.4–22.5%]	3 (3.8%), [0.0–7.9%]	9 (30.0%), [13.6–46.4%]
– Dal/CEA10, CBS 144.89, D141, IHEM6963, NIH 4215, ATCC MYA-1163, AF10	56 (8.7%), [6.5–10.8%]	3 (3.8%), [0.0–7.9%]	1 (3.3%), [0.0–9.8%]
– 10AF/86/10/1, ATCC 90240	35 (5.4%), [3.7–7.1%]	/	/
– NIH 5233, ATCC 13073, B-5233, MF5668	53 (8.2%), [6.1–10.3%]	/	/
– AF216, IHEM 3372, B19119	2 (0.3%), [0.0–0.7%]	/	9 (30.0%), [13.6–46.4%]
– H11-20	4 (0.6%), [0.0–0.5%]	11 (13.8%), [6.2–21.3%]	/
– NCPF 2109, ATCC 46645	40 (6.2%), [4.3–8.0%]	3 (3.8%), [0.0–7.9%]	/
– Ku80/CEA17, FGSC 1152, CEA10:^Δ^Ku80, ^Δ^akuBKU80 (PyrG−)	32 (4.9%), [3.3–6.6%]	/	/
– KU80^Δ^pyrG, FGSC A1160	17 (2.6%), [1.4–3.8%]	/	/
– CNM-CM-AF237, IHEM 5702	13 (2.0%), [0.9–3.1%]	/	1 (3.3%), [0.0–9.8%]
– AfS35	11 (1.7%), [0.7–2.7%]	/	/
– AfS35	9 (1.4%), [0.5–2.3%]	/	/
– P171	/	/	5 (16.7%), [3.3–30.0%]
– Mutant strain(s)	105 (16.1%), [13.2–18.9%]	1 (1.3%), [0.0–3.7%]	1 (3.3%), [0.0–9.8%]
– Local strain(s)	104 (16.1%), [13.2–18.9%]	37 (46.3%), [35.3–57.2%]	1 (3.3%), [0.0–9.8%]
**Route of experimental infection/inoculum size[Table-fn TN3]:**			
– Intravenous injection	213 (31.4%), [27.9–34.9%]	12 (14.6%), [7.0–22.3%]	18 (60.0%), [42.5–77.6%]
	2.5 × 10^7^ (±1.8 × 10^8^), [0.0–5.1 × 10^7^]	1.4 × 10^7^ (±1.1 × 10^7^), [0.7–2.1 × 10^7^]	6.1 106 (±1.8 × 10^7^), [0.0–1.5 × 10^7^]
– Intraperitoneal injection	4 (0.6%), [0.0–1.2%]	2 (2.4%), [0.0–5.8%]	/
	1.2 × 10^8^ (±1.2 × 10^8^), [0.0–4.0 × 10^8^]	8.3 × 10^6^ (±9.4 × 10^6^), [0.0–9.3 × 10^7^]	
– Intranasal deposition	297 (43.7%), [40.0–47.5%]	5 (6.1%), [0.9–11.3%]	1 (3.3%), [0.0–9.8%]
	2.0 × 10^7^ (±5.8 × 10^7^), [1.3–2.6 × 10^7^]	7.2 × 10^6^ (±8.4 × 10^6^), [0.0–1.8 × 10^7^]	1.0 × 10^6^
– Intra-tracheal/intra-bronchial instillation	114 (16.8%), [14.0–19.6%]	55 (67.1%), [56.9–77.2%]	2 (6.7 %), [0.0–15.6%]
	2.2 × 10^7^ (±3.9 × 10^7^), [1.5–3.0 × 10^7^]	2.5 × 10^7^ (±1.4 × 10^8^), [0.0–6.1 × 10^7^]	5.1 × 10^7^ (±7.0 × 10^7^), [0.0–6.8 × 10^8^]
– Inhalation in chamber	55 (8.1%), [6.0–10.2%]	6 (7.3%), [1.7–13.0%]	10 (33.3%), [16.5–50.2%]
	1.8 × 10^9^ (±3.4 × 10^9^), [0.8–2.7 × 10^9^]	6.3 × 10^8^ (±4.3 × 10^8^), [0.2–1.1 × 10^9^]	9.7 × 10^7^ (±9.5 × 10^6^), [0.9–1.0 × 10^8^]
– Intracerebral injection	11 (1.6%), [0.7–2.6%]	1 (1.2%), [0.0–3.3%]	/
	6.3 × 10^6^ (±3.8 × 10^6^), [3.6–9.0 × 10^6^]	6.7 × 10^6^ (±7.4 × 10^6^), [0.0–1.8 × 10^7^]	
– Intraocular injection / scraping	15 (2.2%), [1.1–3.3%]	4 (4.9%), [0.2–9.6%]	1 (3.3%), [0.0–9.8%]
	1.8 × 10^6^ (±5.3 × 10^6^), [0.0–4.8 × 10^6^]	3.4 × 10^7^ (±5.7 × 10^7^), [0.0–1.8 × 10^8^]	2.0 × 10^4^
– Other miscellaneous routes	14 (2.0%), [1.0–3.1%]	2 (2.4%), [0.0–5.8%]	/
	1.9 × 10^8^ (±4.0 × 10^8^), [0.0–4.6 × 10^8^]	6.3 × 10^6^ (±1.5 × 10^6^), [0.0–2.0 × 10^7^]	
**Validation of the model/parameters to follow, *including[Table-fn TN3]:***	545 (79.6%), [76.5–85.6%]	76 (88.4%), [81.6–95.1%]	30 (100.0%), [100.0–100.0%]
– Histopathology	361 (53.2%), [49.5–57.0%]	49 (57.0%), [46.5–67.4%]	11 (33.7%), [19.4–53.9%]
– *In vitro* mycological culture (CFU)	319 (47.1%), [43.3–50.8%]	51 (59.3%), [48.9–69.7%]	23 (76.7%), [61.5–91.8%]
– Galactomannan antigen measurement	33 (4.9%), [3.2–6.5%]	25 (29.1%), [19.5–38.7%]	12 (40.0%), [22.5–57.5%]
– β-D-glucans measurement	5 (0.7%), [0.1–1.4%]	5 (5.8%), [0.9–10.8%]	6 (20.0%), [5.7–34.3%]
– Polymerase chain reaction	91 (13.4%), [10.9–16.0%]	14 (16.3%), [8.5–24.1%]	7 (23.3%), [8.2–38.5%]
– Chitin assay	34 (5.0%), [3.4–6.6%]	4 (4.7%), [0.2–9.1%]	1 (3.3%), [0.0–9.8%]
– Other surrogate biomarker(s)	3 (0.4%), [0.0–0.9%]	2 (2.3%), [0.0–5.5%]	/

*CFU, Colony-forming unit(s); g, grams; /, 0 (0.0%), (0.0–0.0%)*.

Ψ *31 studies used both males and females*.

Δ *One study encompassed males and females*.

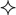
*Associations are possible*.

* *Non-exhaustive listing; when do exist, synonym strain names are provided on the same line*.

*Key-issues of pivotal study parameters are displayed in this table, except when not available in some of the retained articles. Among the 800 articles, five reported the use of several rodent species at a time: two with mice plus rats, and three with mice plus guinea pigs*.

There were significant differences regarding the body weight of the rodents at time of experimental inoculation. For example, weight ranged from 9 g for very young mice (Li et al., 2014) to around 45 g, when they lived up to 42 months (Khosravi et al., 2012). For rats, it spanned from 26.5 g for 11 day old-pups (Zimmerli et al., 2007) to almost 400 g for oldest animals (Sivak et al., 2004a,b; Risovic et al., 2007; Wasan et al., 2007, 2009). Weight for guinea pigs was more homogeneous: 486.1 g ± 36.9. While animal sex was not specified in 152 articles, males were reported to be used in 42.6% of all the selected papers, and females in 41.9%. Both sexes were used without differentiation in 32 works (Supplementary Material 1).

Inbred rodents were more frequently used than outbred, 60.5 vs. 34.5% respectively. Among inbred mice, BALB/c, C57BL/6, and ICR/HaJ were reported in 209, 161, and 55 articles, respectively (Supplementary Material 2), i.e., 30.3, 23.5, and 8.2% of all the experimental studies using mice. When addressing chronic granulomatous disease, C57BL/6 strain was used to induce mutation in gp^91phox^ or gp^47phox^ genes in order to generate deficiency of the oxidative burst in phagocytic cells. A lot of other inbred mouse strains were only sporadically employed, like DBA/2J, CF-1, albino DDY—mainly in Japanese facilities—, or 129/Sv mice, these latter being also particularly useful for production of targeted mutations (Supplementary Material 2). Regarding the outbred strains, CD-1 and albino Swiss Webster mice were the most used, in 122 and 49 papers (Supplementary Material 2), i.e., 17.9 and 7.0% of the reported experiments in this rodent species. Outbred Swiss OF1 and NMRI mice were less employed (Supplementary Material 2), this latter being mostly tested in pharmacology and toxicology studies. Hybrid and congenic mice were generated in 2.5% of the labs using mouse species as animal models of invasive aspergillosis by mating two inbred strains and backcrossing their descendants over several generations. For instance, BALB/c mice were coupled with DBA/2 to get CD2F1 hybrids, or to generate mutation in order to mimic chronic granulomatous disease in B6.129S2 strains (Supplementary Material 2).

For studies with rats and guinea pigs, outbred strains were more often used than inbred ones: Sprague-Dawley represented 51.2% of the reports dealing with rats, while Pirbright white Dunkin-Hartley were associated with 76.7% of assays in guinea pig (Supplementary Material 2). These two strains were judged as excellent multipurpose models for safety and efficacy testing, as were also outbred albino Wistar and albino-CD rats that were tested in 14 and 1 works, respectively. For inbred rats, RP-strain albino, Lewis, Dark Agouti, and albino Oxford strains were used in 15, 3, 3, and 2 articles, respectively (Supplementary Material 2). The last three ones were considered as amenable supports for addressing immunology and inflammation response.

### Selection of the immunosuppressive regimen

Induction of immunosuppression in rodents was reported in 617 publications, i.e., 78.0, 78.8, and 63.3% of articles that included mice, rats, and guinea pigs, respectively (Table 2). It was not the case for some very specific models, especially those for which experimental infection was achieved by intravenous (IV) route (models based on IV route represented 79.5% of the works with no immunosuppressive regimen), or when only local course of invasive aspergillosis was expected like ocular infection (Jie et al., 2009; Zhong et al., 2009, 2016; Ren et al., 2010; Guo et al., 2012; Gresnigt et al., 2014; Taylor et al., 2014a; Jiang et al., 2015, 2016; Li et al., 2015; Xu et al., 2015; Zhao et al., 2015).

Steroids were used in 44.4% articles, especially by the means of subcutaneous (sc) injections of cortisone or triamcinolone acetonide. Dexamethasone was less employed (Baisakh et al., 1975; Bartroli et al., 1998; Clemons et al., 2000b; Ullmann et al., 2007; Zimmerli et al., 2007; Morisse et al., 2012, 2013), sometimes given in drinking water (Meulbroek et al., 2003; García et al., 2006). Prednisolone and methylprednisolone were only rarely administered (Corbel and Eades, 1977; Yamakami et al., 1996; Hashimoto et al., 1998; Paris et al., 2003; McCulloch et al., 2009, 2012; Zhao et al., 2010; Rebong et al., 2011; Alsaadi et al., 2012; Zhao and Perlin, 2013; Zhang et al., 2014). Many dosages were tested for steroids, either in a single administration or in repeated applications. Cortisone sc 100–200 mg/Kg 3 times a week (*tiw*) for the 2 weeks before experimental infection was quite common in mice.

Alkylating drugs were reported to be used in 42.9% papers, and in 24.9% in association with steroids. Intraperitoneal injection of cyclophosphamide at a 150 mg/Kg *tiw* dosage the week before infection was mostly used in mice. Perifosine (Bonifazi et al., 2010), busulphan (Baisakh et al., 1975; Stein et al., 2013), and nitrogen mustards (Schaffner et al., 1982; Schaffner and Frick, 1985; Schaffner and Böhler, 1993) were also tested, but all of them primarily in former works.

Other immunosuppressive medicines were used, like 5-fluorouracil (Hata et al., 1996a,b, 2011; Wallace et al., 1997; Graybill et al., 1998, 2003a; BitMansour et al., 2005; Stojanovic et al., 2011; Salas et al., 2013), tacrolimus (FK506; High and Washburn, 1997; Herbst et al., 2013, 2015; Shirkhani et al., 2015), cyclosporine A (Polak-Wyss, 1991; High and Washburn, 1997), mitotic poisons (Baisakh et al., 1975; Balloy et al., 2005a,b; Loussert et al., 2010; Hein et al., 2015). Tunicamycin was employed once because of its anti-Natural Killer (NK) cell property (Maheshwari et al., 1988), and liposomal dichloromethylene diphosphonate (DMPD) because it decreases macrophages in liver and spleen (Moonis et al., 1994). Interestingly, 5 mg gold sodium thiomalate were injected 1 h before *A. fumigatus* inoculation in mice by Williams et al. (1981).

Forty-five articles recorded usage of immunotherapy. For 24 of them, anti-neutrophil Ly6 (Gr-1) rat IgG_2b_ MAb57 antibody (clone RB6-8C5) was used on the basis of its property to react with mouse Ly-6G, i.e., a 21–25 kDa protein also known as the myeloid differentiation antigen Gr-1, for 24 of them (Supplementary Material 3). Some other antibodies, like anti-asialo GM1 and PK136, were injected to specifically study response due to NK cells (Maheshwari et al., 1988; Tandon et al., 1988; Morrison et al., 2003) or to target CD_4_+ and/or CD_8_+ T-cell lymphocytes (Corbel and Eades, 1977; Carvalho et al., 2012; Cruz et al., 2013). Some models described genetic rough depletion of all B-cell (Montagnoli et al., 2003) and T-cell lymphocytes (Maheshwari et al., 1988; Tandon et al., 1988).

Besides in 21 studies, 6–9 Gy irradiation was enforced to rodents in order to mimic total blood-cells depletion during bone marrow transplantation (Supplementary Material 3).

Genetic mutations were sometimes induced in some targeted rodent genes, when studying role of particular immunological pathways, for instance those involving the cytokines, receptors, proteins, enzymes associated with immune response, and activation of immunoglobulins, like MyD88, Card9, Rag1 and Rag2, PTX3, TRIF, and many others (Supplementary Material 3). Mutations in gp^91phox^ or gp^47phox^ genes were generated to mimic chronic granulomatous disease (Supplementary Material 3). Notably, an original model of surfactant protein (SP)-deficient mice was used to show that absence of SP-D protein made rodents more susceptible to invasive aspergillosis than the absence of SP-A (Brieland et al., 2001).

Alternative procedures were developed to allow invasive aspergillosis in very original opportunistic contexts, like septic infections following caecal ligation and puncture (Benjamim et al., 2003, 2005), as well as cancerous disease using 1.0 × 10^6^ S-180 tumor cells suspension (Okawa et al., 2002), and rhinosinusitis after unilateral nasal obstruction with sponges (Zhang et al., 2013). In addition to all these abovementioned methods, rodents were sometimes fed with low-diet hypo-protein regimen to mimic the malnourished status of the weakest human patients that are usually infected with *A. fumigatus* in hospitals (Miyazaki et al., 1993; Mitsutake et al., 1995; Chandenier et al., 2009; Lo Giudice et al., 2010; Desoubeaux and Chandenier, 2012; Desoubeaux et al., 2014b).

In order to prevent any undesirable bacterial infection during experiments, antibiotics were reported to be administered in 181 works, that means in only 27.7% of the studies in which immunosuppression was induced. Variability of the drug(s) to be used for such a purpose was very high. Because they are cheap, cyclins, and especially tetracycline at a dosage of 1 mg/mL in drinking water, were mostly provided to rodents (Supplementary Material 4). Quinolones, usually administered in gavage food or in drinking water like enrofloxacin at a 5–8 mg/kg daily dosage, were second choices (Supplementary Material 4). They were sometimes dispensed in association with sulfamethoxazole-trimethoprim (Chang et al., 2004; Ito et al., 2006), oral cyclins (Aufauvre-Brown et al., 1997; Brown et al., 2000; Balloy et al., 2005b; McDonagh et al., 2008; Herbst et al., 2013, 2015; Huber and Bignell, 2014), or intra-muscular (IM) teicoplanin (van Vianen et al., 2006; Petrik et al., 2010, 2012, 2014; Verwer et al., 2013). Cephalosporins were a quite frequently-used alternative (Supplementary Material 4): ceftazidime was sc- or IM-injected at a 50 mg/Kg daily dosage. Oral and IM usage of aminosides was rarer (Supplementary Material 4). Injectable glycopeptides and carbapenems were sporadically used to prevent infections due to multi-resistant staphylococci and enterobacteriae (Melchers et al., 1994; Hashimoto et al., 1998; Yonezawa et al., 2000; Benjamim et al., 2005; Steinbach et al., 2006; Tansho et al., 2006; van Vianen et al., 2006; Cramer et al., 2008; Pinchai et al., 2009; Rivera et al., 2009; Petrik et al., 2010, 2012, 2014; Grahl et al., 2011; Martinez et al., 2013; Verwer et al., 2013). Likewise, multi-antibiotic associations were sometimes reported, like the following scheme: ciprofloxacin (660 mg/L) and polymyxin B (100 mg/L) in water, plus IM amoxicillin (40 mg/kg/day) and a single shot of IM gentamicin (6 mg/kg) at time of experimental infection (Leenders et al., 1996; Dams et al., 1999; Becker et al., 2000, 2002a,b, 2003, 2006; Van Etten et al., 2000; Ruijgrok et al., 2001, 2005, 2006). Interestingly, a few authors preconized to dilute halogenated derivatives, like chlorine or iodine, as disinfectants in drinking water (Corbel and Eades, 1977; Xu et al., 2015). To note, six studies clearly asserted that antibiotic usage was not required, because of low incidence rate of opportunistic bacterial infection that they had noticed in the past works (de Repentigny et al., 1993; Kretschmar et al., 2001; Lewis et al., 2002; Chaturvedi et al., 2005; Iannitti et al., 2013; Speth et al., 2013).

### Choice of the *aspergillus fumigatus* strain

Overall, 230 distinct *A. fumigatus* strains were reported in the literature for experimental infection (Table 2). The analysis was made difficult because of the global use of synonyms and unofficial alternative strain names.

AF293, also known as ATCC MYA-4609, FA/1153, FGSC A1100, CBS 101355, NCPF 7367, or IHEM18963, was the most used strain, including sometimes its derivatives like the fluffy variant (Ben-Ami et al., 2010b) or the Af293.1 (Bok et al., 2005, 2006; Tsitsigiannis et al., 2005; Cramer et al., 2006; Romano et al., 2006; Dagenais et al., 2008; Ma et al., 2008; Qiao et al., 2008; Lee et al., 2009; Han et al., 2010; Leal et al., 2010, 2012, 2013; Jhingran et al., 2012; Sekonyela et al., 2013; Taylor et al., 2014b; Kerr et al., 2016) and Af293.6 mutants (Dagenais et al., 2008; Leal et al., 2012; Sekonyela et al., 2013; Kerr et al., 2016), in 18.3% of the experimental infections (Supplementary Material 5). It was followed by Dal/CEA10, also referred to as CBS 144.89, D141, IHEM6963, NIH 4215, ATCC MYA-1163, and AF10, in 8.0% of the cases (Supplementary Material 5). Ku80 strains, like CEA10: ΔKu80, also named Ku80/CEA17 or FGSC 1152, which is one of Dal/CEA10 derivate, have been largely used because they increased homologous recombination for gene replacement. There are Ku80 pyrG+ and pyrG– strains, and the ones that are auxotrophic mutants in the uracil/uridine pathway express attenuate virulence. H11–20 strain was chosen in 15 works, but mostly in rat models (Supplementary Material 5), as it was first isolated from rat dying of spontaneously-acquired aspergillosis while on steroid treatment.

Some *A. fumigatus* strains were selected for their very specific features like bioluminescent AfC3 (Brock et al., 2008; Ibrahim-Granet et al., 2010; Fekkar et al., 2012; Jouvion et al., 2012; Morisse et al., 2012) or Af 2/7/1 (Galiger et al., 2013; Savers et al., 2016). Among other examples, one should notice for instance AF91 (also maned NCPF 7100, IHEM 13936, or J960180) which expresses attenuated virulence (Denning et al., 1997a,b; Dannaoui et al., 1999; Overdijk et al., 1999; Warn et al., 2003, 2006, 2010; Paisley et al., 2005), or EMFR S678P that resists to echinocandins (Miyazaki et al., 1993; Lepak et al., 2013a,b,c) and AZN 58 for which flucytosin is not active (Verweij et al., 2008). V28–77 and V59–73 are azole-resistant strains that were employed to assess impact of M220I mutation and G54 substitution in the gene coding Cyp51A upon the pharmacokinetics and pharmacodynamics properties of voriconazole (Mavridou et al., 2010a,b; Seyedmousavi et al., 2015).

In addition, 105 articles reported usage of reference strains but genetically modified by specific mutation/deletion, like Δ*gliA* (Cramer et al., 2006; Kupfahl et al., 2006; Sugui et al., 2007a; Chiang et al., 2008) and Δ*gliP* (Wang et al., 2014), Δ*chsC* (Mellado et al., 1996; Aufauvre-Brown et al., 1997) and *chsG* (Mellado et al., 1996), Δ*sebA* (Dinamarco et al., 2012a,b), Δ*catA* (Paris et al., 2003; Ben-Ami et al., 2010a; Ben-Ami and Kontoyiannis, 2012; Leal et al., 2013), or Δ*aspB* (Vargas-Muñiz et al., 2015). They were usually tested in virulence studies that focused on fungal factors like proteins of the cell wall integrity, elastase, and other miscellaneous proteases (Kothary et al., 1984; Kolattukudy et al., 1993; Frosco et al., 1994), catalases (Paris et al., 2003), phospholipase, toxins (Paris et al., 1993; Bok et al., 2006; Cramer et al., 2006; Sugui et al., 2007a,b; Gravelat et al., 2008; Ben-Ami et al., 2009; Wang et al., 2014), adhesins, restrictocins (Smith et al., 1993), conidial pigments like melanin (Jahn et al., 1997; Langfelder et al., 1998), histidine kinase (Bartroli et al., 1998; Clemons et al., 2002; Du et al., 2002), calcineurine (Steinbach et al., 2006; Juvvadi et al., 2013), alkaline protease (Monod et al., 1993; Tang et al., 1993; Jaton-Ogay et al., 1994; Smith et al., 1994), and chitin synthase (Mellado et al., 1996; Aufauvre-Brown et al., 1997).

Moreover, 141 manuscripts reported usage of miscellaneous strains that had been locally isolated and that were not referenced in official collections (Supplementary Material 5).

### Implementation of the experimental infection

Size of the fungal inoculum for the experimental infection varied greatly (Table 2), depending especially on the *A. fumigatus* strain(s) selected (Johnson et al., 2000; Takemoto et al., 2004, 2006; Cacciapuoti et al., 2006; Warn et al., 2006, 2010; Mavridou et al., 2010a,b), the aims of the study [e.g., Gao et al., doubled the inoculum when studying histopathology (Gao et al., 1997), whereas O'Hanlon et al. increased it 6-fold for the same purpose (O'Hanlon et al., 2011)], as well as the immunocompromised status of the animals (globally, the more immunocompromised are the rodents, the lower inoculum is needed to induce aspergillosis in them) and the route of administration (Mehrad et al., 1999; Cenci et al., 2000; BitMansour and Brown, 2002; Bozza et al., 2003; Chang et al., 2004; Takemoto et al., 2004, 2006; Grahl et al., 2012; Slesiona et al., 2012; Juvvadi et al., 2013; Wharton et al., 2015).

For instance, inoculum deposited into the rodents was particularly low when *A. fumigatus* spores were aerosolized into an inhalation chamber, like the acrylic Hinners' chamber described by Steinbach et al. and the large-scale inhalational Madison chamber: around 1.0 × 10^4^ conidia *per* animal, although a larger fungal suspension was needed to generate the inoculum (Steinbach et al., 2004, 2006; Cramer et al., 2008; Pinchai et al., 2009; Leleu et al., 2013a,b; Lamoth et al., 2014a,b): the mean size was ~1.4 × 10^9^ conidia/mL, extreme values ranging from 1.0 × 10^3^ (Ahmad et al., 2014) to 1.2 × 10^12^/mL (Chiang et al., 2006; Gravelat et al., 2008; Evans et al., 2010a; Ibrahim et al., 2011), in a 10–40 mL-suspension (mean: 12 mL) spread during 25 min to 1 h, with a flow rate of 100–200 kPa (1–2 bar). Out of all the articles retained for analysis, 8.8% used such devices (Supplementary Material 6). In 304 works, the intranasal route was privileged. Out of them, 297 used mice, which represented 43.7% of all the articles dealing with this rodent species: an average of 2.0 × 10^7^ *A. fumigatus* spores were instilled in nares (min: 3.0 × 10^3^—max: 5.0 × 10^8^). In contrast, the intranasal route was more rarely reported for rats (mean inoculum size: 7.2 × 10^6^, min: 5.0 × 10^3^—max: 2.0 × 10^7^; Hachem et al., 2006; Morisse et al., 2012, 2013; Zhang et al., 2013; Yan et al., 2014), in part because their respiratory apparatus is longer and their alveoli harder to reach. Instead in them, the bronchial-tracheal instillation was primarily chosen in 67.1% of the papers (Supplementary Material 6): the mean inoculum size was 2.5 × 10^7^ spores, and spanned from 1.0 × 10^3^ (Khan et al., 2008) to 1.0 × 10^9^ (Land et al., 1989), whereas it was quite close to this in mice undergoing the same bronchial-tracheal procedure, 2.2 × 10^7^ (min: 1.5 × 10^2^—max: 2.0 × 10^7^; Björgvinsdóttir et al., 1997; Bozza et al., 2002b, 2003; Garlanda et al., 2002; Montagnoli et al., 2003). In most of the cases, spores suspension was most often instilled into the trachea or directly into the lungs after tracheotomy or small thoracotomy (Habicht et al., 2002). In some studies, a cannula was passed into the trachea through the mouth, and then one of the lung lobes (Balloy et al., 2005b; Ruijgrok et al., 2005; Nagasaki et al., 2009). Passing through the upper airways, the MicroSprayer® Aerosolizer device was proven to generate an air-dispersed controlled cloud of conidia in bronchial-tracheal apparatus of rats, mimicking then the real pathophysiology of airborne *A. fumigatus* exposure (Chandenier et al., 2009; Desoubeaux and Chandenier, 2012; Desoubeaux et al., 2014b). To our knowledge, only one research facility employed bronchial-tracheal route in guinea pigs (Chandrasekar et al., 2000, 2004).

IV route was used to generate rapid fungal dissemination through bloodstream. It usually required a smaller *A. fumigatus* inoculum, regardless the immunocompromised status of the rodents: 2.3 × 10^7^ spores in average, extremes ranging from 5.0 × 10^1^ (Cutsem et al., 1993) to 2.5 × 10^9^ (Bowman et al., 2001), and its usage was reported in 30.0% of the selected articles of this review. Jugular vein (Sivak et al., 2004a,b; Risovic et al., 2007), femoral vein (Wong et al., 1989), and penis vein (Overdijk et al., 1996; Reichard et al., 1997; Odds et al., 1998) were generally chosen for such a purpose in large rodent species, while lateral tail vein was privileged in mice (Odds et al., 1998). Retro-orbital vein was an alternative, but has become less frequent in recent years for ethical considerations (te Dorsthorst et al., 2005; Verweij et al., 2008; Wagener et al., 2008; Mouton et al., 2009; Dirr et al., 2010; Kotz et al., 2010; Heesemann et al., 2011). To provide free movement for the animal and to make easier the IV administration, Odds et al. and Meerpoel et al. connected a catheter to the ligated jugular vein via a proprietary swivel device (Odds et al., 2000; Meerpoel et al., 2010). To induce endocarditis in guinea pigs, Martin et al. injected 1.0 × 10^4^ spores in the internal carotid, passing a catheter through the left auricle and just through the mitral valve (Martin et al., 1997).

Alternative route of experimental infection were sometimes studied to address some very specific forms of invasive aspergillosis, like local eye invasion during endophthalmitis or ulcerative keratitis, and cerebral infection (Supplementary Material 6). For ocular infection, the corneal epithelium was abraded by needle (Carrion et al., 2013) or totally removed with a paracentesis knife that perforated cuts perpendicular to each other (Ren et al., 2010), then the damaged region was either smeared with conidia (Zhong et al., 2016) or directly injected (Zhong et al., 2009); but in both situations, great difficulties were encountered to reliably adjust the inoculum size. For the models of cerebral aspergillosis, the investigators injected *A. fumigatus* spores directly into the brain resulting in local high tissue burdens (Chiller et al., 2002). For instance, Mozzala et al. used a 0.1 mL glass micro-syringe associated with a 27-gauge disposable needle to go through the central area of the frontal bone of mice to a depth of 2 mm (Mazzolla et al., 1991). In rats, Zimmerli et al. proposed to reach the *cisterna magna* using an innovative procedure that does not produce structural brain damage. It appeared to be well-tolerated by all animals as no mortality was observed during the first 12 h after injection (Zimmerli et al., 2007). For a nephritis model, 1.0 × 10^3^ spores were injected into the intra-medullar kidney (Walzl et al., 1987; Schaude et al., 1990). Cutaneous abscesses were generated in murine soft tissues by sc injections, mainly into the thigh after fur removal (Lupetti et al., 2002; Ruiz-Cabello et al., 2002; Donat et al., 2012), or by intradermal injections into the ears (Petersen et al., 2002; Goebel et al., 2005; Stein et al., 2013).

Altogether, the frequency of spore administration to induce experimental infection was generally based on a single application, except for some specific studies that performed daily inoculations over 2 or 3 days (or even over a longer period; Smith et al., 1994; Cenci et al., 1998, 1999, 2000, 2001, 2002; Del Sero et al., 1999; Bozza et al., 2002a; Du et al., 2002; Bellocchio et al., 2004a,b, 2005; Gaziano et al., 2004; Mellado et al., 2005; Shao et al., 2005a; Mazaki et al., 2006; Zelante et al., 2007, 2009, 2015; D'Angelo et al., 2009; Morton et al., 2010, 2012; Moretti et al., 2012, 2014; Zhang et al., 2013; Hein et al., 2015), or repeated spaced administrations (Smith, 1972, 1973, 1977; Turner et al., 1975a,b; Morton et al., 2012; Turner et al., 1976; Lehmann and White, 1976; de Repentigny et al., 1993; Cenci et al., 1997; Mazaki et al., 2006; Fei et al., 2011; Templeton et al., 2011; Alcazar-Fuoli et al., 2015; Savers et al., 2016), sometimes in order to induce immune protection (Centeno-Lima et al., 2002) or to enhance the infection yields, particularly for inhalational models in chamber (Buskirk et al., 2014).

### Validation of the model and outcome parameters to follow up

Overall, 81.6% of the studied literature reported usage of at least one test to confirm that the experimental infection has been correctly implemented in rodents or to assess the fungal burden (Table 2), and 38.6% noticed the concomitant usage of two or more distinct techniques. Histopathology and *in vitro* mycological culture have been more largely described, in 52.8 and 49.0% articles, respectively, sometimes concomitantly in 25.6% of the cases (Supplementary Material 7).

More recent methods include the detection of GM, (1 → 3)β-D-glucans, and fungal DNA by polymerase chain reaction (PCR; Supplementary Material 7). In 4.9% of the animal studies, the chitin assay was used for assessment of fungal burden in fluids or homogenized tissues (Supplementary Material 7).

Some investigators analyzed the host immune response as outcome measure (Morgenstern et al., 1997; Cenci et al., 1998, 1999; Duong et al., 1998; Brieland et al., 2001; Shao et al., 2005b; Steele et al., 2005; Bonnett et al., 2006; Montagnoli et al., 2006; Cornish et al., 2008; Herbst et al., 2013; Kasahara et al., 2016; Renshaw et al., 2016). For instance in bronchial-alveolar lavage (BAL) fluids, they measured cytokine concentrations through ELISA assays, and addressed representation changes of lymphocyte cells or phagocytic cells by flow cytometry. NADPH-oxidative pathway was also investigated as markers for evolution of the infection (Aratani et al., 2002; Philippe et al., 2003; Cornish et al., 2008; Stein et al., 2013; Prüfer et al., 2014; Röhm et al., 2014).

Detection of anti-*Aspergillus* antibodies was sometimes performed (Turner et al., 1975b, 1976; Naik et al., 2011). In human medicine it is nonetheless not considered as a biomarker of invasive infection, but rather of chronic or allergic aspergillosis. To attest of the consequences of invasive infection, blood urea nitrogen, creatinine, serum glutamic pyruvic transaminase (ALAT), and serum glutamic oxaloacetic transaminase (ASAT) in serum were measured as indirect but unspecific surrogate endpoints (Singh et al., 2014).

## Discussion

Animal models of invasive aspergillosis have been developed to make the link between *in vitro* experiments and clinical trials. They have been used extensively to study various aspects of pathogenesis, innate and acquired host-response, disease transmission, diagnostic tools assessment, and preclinical therapy during aspergillosis (Clemons and Stevens, 2005, 2006a,b). Theoretically, a perfect unique model, i.e., highly reproducible, economical and standardized, should be expected to address reproducibly all these issues (Najvar et al., 2004; Patterson, 2005). This present overview attempted to summarize all the technical parameters reported in the literature about rodent models of invasive infection caused by *A. fumigatus*, but was not intended to detail the wealth of insight gained from them into the pathogenis, host immune response, diagnosis, and treatment, and the reader is invited to refer to other publications (Sable et al., 2008; Brown, 2011; Steele and Wormley, 2012; Wüthrich et al., 2012; Drew et al., 2013; Lanternier et al., 2013). Overall, it highlighted great variations regarding all the experimental settings of the lab models. Actually, such heterogeneity has always existed in disease models in other systems (Maarman et al., 2013; Golden et al., 2015), and this is rather a general problem in research and not exclusive to aspergillosis, but herein, the variables were both related to the host and the pathogen factors, as well as to the route of infection and the size of fungal inoculum. These discrepancies likely resulted in great differences on the study results. For instance, combination of caspofungin (an echinocandin drug) and liposomal amphotericin B (a polyen), as well as the association of caspofungin with amphotericin B lipid complexes, was shown to have no significantly enhanced activity in a cerebral model of aspergillosis (Luque et al., 2003; Clemons et al., 2005; Imai et al., 2005), whereas in contrast, micafungin (another echinocandin) had reduced activity and even possible enhanced drug toxicity with triamcinolone against pulmonary disease in steroid-suppressed mice (Clemons and Stevens, 2006c). In this latter model, it was also found that the combination of micafungin and itraconazole was antagonistic, highlighting a decrease in efficacy (Clemons and Stevens, 2006c), while it was not in a systemic model (Luque et al., 2003). Investigating the pathogenesis of gliotoxin-producing and non-producing isogenic strains of *A. fumigatus*, a series of studies demonstrated that the secondary metabolite contributes to virulence in a non-neutropenic murine model of disease, through its effects on NF-κB-dependent host cell apoptosis and on phagocyte NADPH oxidase function, but not in neutropenic murine models (Kupfahl et al., 2006; Sugui et al., 2007b; Spikes et al., 2008). Beyond these few examples, several other studies also demonstrated that drugs may have different therapeutic effects and virulence factors have distinct impacts depending on the chosen model (Graybill et al., 2003a). Thus, a minimum standardization seems necessary to reliably compare the results between laboratories.

First, choice regarding the rodent species and strain is critical to ensure correct reproducibility (Mitsutake et al., 1995; Durrant et al., 2011; Mirkov et al., 2014). Of course, murine models have predominated for most investigators over the years. Regarding their respective physiology, it is indeed acknowledged that mice and humans have similarities in organ systems, biochemistries, pathologies, and even in their two genomes that both encompass ~30,000 genes and for which the proportion with no homology between them is < 1% (Mouse Genome Sequencing Consortium et al., 2002). Moreover, the limited body size of mice allows usage of a relatively large number of animals to be tested simultaneously under identical conditions, which is of course relevant to perform statistical analysis. In addition, many commercial reagents and kits are readily available to aid in studies. Furthermore, in the light of all the genetically-defined mouse strains that are currently available, scientists have the great possibility to select the most appropriate host factors they need for mimicking specific clinical situations and to generate an infection according to a well-defined pattern (Pollock et al., 1995; Morgenstern et al., 1997; Dennis et al., 2006; Lengerova et al., 2012; Leleu et al., 2013b). For instance, BALB/c mice are a well-known general multipurpose model allowing studying for infectious diseases, while C57BL/6 strain represents permissive background for maximal expression of most mutations, like those in genes coding for cytokines, toll-like receptors (TLRs), Dectin-1 or other receptors/proteins associated with immune response (Steele et al., 2005; Carrion et al., 2013; Herbst et al., 2013, 2015; Leal et al., 2013; Shepardson et al., 2013; Bozza et al., 2014; Espinosa et al., 2014; Moretti et al., 2014; Taylor et al., 2014b; Caffrey et al., 2015; Jhingran et al., 2015; Karki et al., 2015; Wharton et al., 2015; Zelante et al., 2015; Kasahara et al., 2016; Savers et al., 2016). Usage of genetically-deficient knockout (KO) mice provided new insights upon the pathophysiology of invasive aspergillosis (Deepe et al., 2000). For example, it proved that Interleukin- (IL-)6, IL-12 and interferon-γ (INFγ) were protective factors against *A. fumigatus*. In contrast, IL-10 and IL-4 deficiency made respective KO-mice more resistant to infection (Cenci et al., 1998, 2001; Del Sero et al., 1999; Clemons et al., 2000a): neutralization of IL-10 was reported to up-regulate production of nitric oxide, contributing to an effective fungicidal (Romani et al., 1994), and IL-4 cured 70% of infected mice when administered exogenously while protecting them from a second lethal challenge (Cenci et al., 1997). Importantly, KO-models also showed that IL-17, as well as TLR-4 and TLR-2 are of great importance in the innate response against *A. fumigatus*. For instance when they are TLR-2 KO, mice had low tumor necrosis factor-(TNF)-α and IL-12 rates, as well as reduced survival and higher fungal burdens in the tissues than competent mice (Bellocchio et al., 2004a; Balloy et al., 2005b). In a near future, it is expected that the improvement of all the molecular tools will be able to provide more and more genetically modified rodent strains. It is also noteworthy that AKR/J, C57BL/6, 129/SvJ, and BALB/c inbred strains were shown to be more resistant to *A. fumigatus* infection than MRL/MPJ and NZW/LacJ mice which were themselves more resistant than DBA/2 (Zaas et al., 2008). By the way, these latter are complement deficient, and their susceptibility to invasive aspergillosis sheds light on the role of complement in host defense against the fungus. One of the advantages of all the aforementioned inbred strains relies in that they express less host genetic variability (Festing, 2010), although some slight differences have been raised up for DBA/2 and BALB/c strains between Great Britain and U.S.A. (Hector et al., 1990). However, one could argue that invasive aspergillosis actually occurs in genetically-not defined human patients which probably exhibit great genomic heterogeneity (Goldman and Osmani, 2007). In contrast, outbred strains are genetically randomized, and their phenotypic background is not totally controllable (Chia et al., 2005). As evidenced by several examples with Albino Swiss Webster before the year 2000 (Sandhu et al., 1970, 1976; Smith, 1972, 1973; Baisakh et al., 1975; Lehmann and White, 1975, 1976, 1978; Ghosh et al., 1977; White, 1977; Saeed and Hay, 1981; Polak, 1982, 1987; Polak et al., 1982; Van Cutsem et al., 1984, 1987; Dixon, 1987; Maheshwari et al., 1988; Tandon et al., 1988; Dixon et al., 1989; Hector et al., 1990; Clark et al., 1991; Polak-Wyss, 1991; Paris et al., 1993; Thau et al., 1994; Wiederhold et al., 2004; Lewis et al., 2005), they should only be considered during drug development for pre-clinical screening studies, when specific host factors are not assumed to be critical (Clemons and Stevens, 2006a). In contrast, outbred mice are not suited to precise pharmacology/pharmacokinetics studies, because for example their gut mucosae enable too rapid metabolism for azole drugs (Sugar and Liu, 2000; MacCallum and Odds, 2002; Graybill et al., 2003b). Although the mouse strains are less expensive, use of bigger animals, like guinea pigs, have the advantage to allow serial sampling, like repeated blood sampling as well as BAL. Moreover, guinea pigs do not express an acute infection pattern, and thus likely allow reducing the number of animals to be used by reducing rapid mortality (Riera et al., 1983; Capilla and Guarro, 2004). Doses and antifungal regimens can also be easily monitored and modified (Reichard et al., 1997; Chandrasekar et al., 2004; Clemons and Stevens, 2005; Vallor et al., 2008; Wiederhold et al., 2009, 2013, 2015; Dufresne et al., 2012; Hooper et al., 2012; Kirkpatrick et al., 2012; Lengerova et al., 2012; Jambunathan et al., 2013; Zhao et al., 2015; White et al., 2016), especially for addressing clinical efficacy and pharmacodynamics/pharmacokinetics of echinocandin or azoles (Van Cutsem et al., 1989, 1990; Cutsem et al., 1993; Arrese et al., 1994; Overdijk et al., 1996, 1999; Reichard et al., 1997; Odds et al., 1998, 2000; Kirkpatrick et al., 2000b, 2002, 2006; Loeffler et al., 2002; MacCallum et al., 2005; Meerpoel et al., 2010). Indeed, the *in vivo* metabolism in guinea pigs is thought to be comparable to this in humans because of a slow clearance (Graybill et al., 2003b). Using an interesting endocarditis model in guinea pigs (Martin et al., 1997), some investigators were able to highlight the superiority of voriconazole over itraconazole to cure aspergillosis. Nonetheless, in spite of all their valuables features, guinea pigs have been poorly used so far, maybe because they express complex social structure and are stressed under unfamiliar environments or the experimental manipulations (Hennessy, 1999). In such a context, rats may be considered as an interesting compromise between mice and guinea pigs. Rats have been most often used to study invasive aspergillosis with initial pulmonary course (Habicht et al., 2002; Chandenier et al., 2009; Desoubeaux and Chandenier, 2012; Desoubeaux et al., 2014b). Rabbit models (Kurup, 1984; Komadina et al., 1985; Longman and Martin, 1987; Patterson et al., 1988, 1989; Singh et al., 1990; Berenguer et al., 1995; Walsh et al., 1995; Mylonakis et al., 1997; Kirkpatrick et al., 2000a; Roberts et al., 2000; Petraitiene et al., 2004; Clemons and Stevens, 2005; Hao et al., 2008; Petraitis et al., 2009), as well as those developed in birds (Ghori and Edgar, 1979; Chaudhary et al., 1988; Suleiman et al., 2012; Melloul et al., 2014) or in other animals like cows (Jensen et al., 1996), sheep (Corbel et al., 1973; Boase et al., 2011), monkeys (Mahajan et al., 1978), or invertebrates (Lionakis et al., 2005; Chamilos et al., 2010; Cheema and Christians, 2011; Lionakis and Kontoyiannis, 2012; Gomez-Lopez et al., 2014) have also been tested. Invertebrates like *Drosophila melanogaster, Danio rerio, Caenorhabditis elegans*, and *Galleria mellonella* were shown to be useful to study drug distribution, toxicology and metabolic stability, but their highly-simplistic physiology is far much different from that in humans (Giacomotto and Ségalat, 2010). In contrast, work in rabbits appeared relevant, mainly because this species is highly susceptible to infection, and allows serial sampling and easy administration of drugs (Schmidt, 2002). The ability to visualize anatomic details by computed tomography is particularly advantageous to check the progression of focal aspergillosis (Walsh et al., 1995; Petraitiene et al., 2002). However this animal model is more expensive, requires specific facility for this husbandry, and is limited by the availability of a few immunological and biomolecular reagents. There is also a lack of genetically-defined rabbit strains (Capilla et al., 2007).

Another point to thoroughly discuss is the immunosuppressive regimen to administer to the rodents. Since many human patients with invasive aspergillosis are rendered immunocompromised by prior exposure to cytotoxic chemotherapy and/or steroids (Bitar et al., 2014), most of the animal models have included one or both of these medications in order to facilitate correct implementation of *A. fumigatus* disease. Cytotoxic agents, like alkylating drugs e.g., cyclophosphamide or cytosine arabinoside, bind to DNA during cellular replication and thus induce profound neutropenia (Johnson et al., 2000; Chandenier et al., 2009; Desoubeaux and Chandenier, 2012). The histological and radiological features of models treated with alkylating drugs were very close to those of profoundly neutropenic infected patients, like those undergoing leukemia (Chandenier et al., 2009): Fungal growth, dissemination, and destruction of parenchymal architecture by invasive hyphae is the primary mechanism of tissue injury and death. However, the neutropenic models are currently becoming less relevant, since the characteristics of human patients infected with *A. fumigatus* are progressively changing and have less such traditional risks for invasive disease (Upton et al., 2007; Lortholary et al., 2011). Steroids use in mice showed distinct pattern of pathogenesis in comparison with neutrophil-depleting drugs: in steroid-treated rodents, fungal growth is significantly reduced in comparison to chemotherapy-treated animals, and the massive influx of functionally impaired leukocytes triggers dysregulated responses associated with tissue damage, hypoxia, and immunopathology (Balloy et al., 2005a; Grahl et al., 2011). Indeed, steroids affect alveolar macrophage function, and thus reduce the first barrier to pulmonary infection. They also impact T- and B-cell lymphocytes, and they decrease the production of cytokines, which compromises the adaptive immune response against invasive aspergillosis (Tang et al., 1993). In experimental works, dexamethasone was less effective than hydrocortisone or triamcinolone to induce aspergillosis (Marr et al., 2004). Convincingly, the combination of both alkylating and steroid drugs seems valuable to increase lethality in infected animals (Dixon et al., 1989). Some non-neutropenic immunocompromised models have been developed with cyclosporine A and/or steroids to stimulate long-term post-engraftment immune-impaired conditions of bone marrow transplant recipients (Lengerova et al., 2012; Leleu et al., 2013b), while persistent neutropenic supports may be generated by neutrophil-depleting monoclonal antibodies, like IgG_2b_ MAb57 antibody (Mehrad et al., 2002; Richie et al., 2007a,b; Park et al., 2010; Kapp et al., 2014; O'Dea et al., 2014). Regardless of method, a regular monitoring of leucocytes count is recommended to ensure a correct achievement of the immunocompromised status (Stephens-Romero et al., 2005). However, it is important to underline herein that any experimental immunosuppression can affect the host-response to infection, and *de facto* enhances complexity of the model understanding. These effects should be clearly defined, as they actually impact the final conclusions of the study (Balloy et al., 2005a). For instance although nude mice have no mature T-cell lymphocytes, their macrophages were described as being at a higher basal state of activation, and they had increased numbers of NK cells (Cheers and Waller, 1975), so that they experienced potential paradoxical resistance during early during infection. Therefore, it is imperative not to extrapolate data to other susceptible or non-susceptible host states in the absence of experimental confirmation.

Only a few articles out of all the literature actually mentioned anti-opportunistic antibiotic prophylaxis, when rodents underwent immunosuppressive regimen, although undesirable bacterial infections had been shown to hamper and precede correct development of experimental fungal infection (Clemons et al., 2006a). Thus, every effort to prevent them appears worthwhile (Schmidt, 2002). Specific pathogen-free animals represent a valuable option, but their cost makes them less available, especially for the research facilities that are not equipped with adapted sterile housing conditions. Instead, three antibiotic schemes have been mainly used, alone or in combination: cyclins or quinolones provided through the beverage (Yu et al., 1990; Pollock et al., 1995; Cenci et al., 1997; Martin et al., 1997; Niwano et al., 1999; Morisse et al., 2012; Herbst et al., 2013), and sc ceftazidime (Kirkpatrick et al., 2012; Lengerova et al., 2012; White et al., 2016). The oral route may actually be considered as only suboptimal as it results in variable exposure. Indeed, drinking water which contains antibiotics tends to be progressively less intake by rodents as their infection progresses (Chandenier et al., 2009), and oral bio-availability is low for the above-mentioned antibiotics (Cunha et al., 1982). Likewise, injection of third generation-cephalosporin with large antibiotic spectrum have effects upon cytokine expression and disruption to normal microflora in the host. Thus, ceftazidime, which displays anti-*Pseudomonas* and anti-*Enterobacteriaceae* activities, may play a deleterious role when studying pathophysiology during aspergillosis (Cramer et al., 2008; Pinchai et al., 2009).

Regarding the route of experimental challenge, IV inoculation primarily induces overwhelming systemic disseminated infection in rodents (Kirkpatrick et al., 2000b; Seyedmousavi et al., 2013). It is probably the easiest route to standardize, because all the *A. fumigatus* inoculum is directly and entirely injected into the bloodstream through an accessible vein. Liquid fungal suspension can be precisely quantified and calibrated for minimal inter-experimental variability. Thus, IV route evidenced an excellent infection/dose-to-mortality ratio, and did not systematically require a preceding immunosuppression of the animals (Schmidt, 2002): without immunosuppressive regimen, an inoculum seized around 1.0 × 10^7^ *A. fumigatus* conidia *per* mouse was largely enough to lead to an acute and reproducible infection (Paulussen et al., 2014, 2015). On the contrary, administration of cyclophosphamide or cortisone in such a context led to higher variation in clinical outcome. Thus in immunocompetent guinea pigs, the IV route has been particularly useful in pre-clinical therapeutic studies, as well as in assessing the kinetics of diagnostic markers (Kirkpatrick et al., 2000b), meanwhile reducing the number of animal experiments (Kirkpatrick et al., 2013). However, the IV route is of course estimated as an unnatural process, especially because this experimental inoculation procedure does not recapitulate the real one during aspergillosis bypassing mucosal host defense, and because it involves unusual organs, like kidneys (Andriole et al., 1992). Likewise, intra-abdominal infection should not be privileged, since it is not clinically relevant, and rodents often resist to this artificial route of inoculation. In comparison, experimental challenge through the respiratory airways consistently mimic the natural entry into the human body and leads first to the development of invasive aspergillosis in lungs (Andriole et al., 1992; Sheppard et al., 2004, 2006; Steinbach et al., 2004). Nevertheless, it systematically requires a prior immunosuppression to be reproducible (Denning et al., 1995), except for models simulating chronic granulomatous disease (Morgenstern et al., 1997; Philippe et al., 2003). It is harder to standardize because the number of fungal elements arriving in lung tissue is generally uncertain, and all the animals do not react stereotypically (Sheppard et al., 2004; Steinbach et al., 2004). Consequently, respiratory challenge requires a higher number of rodents to be statistically relevant (Latgé, 1999). Besides, organ tropism remains a little bit different from the primary tissues that are usually infected in human patients: substantial renal involvement is high following pulmonary infection in mice (Clemons and Stevens, 2005). Given that they allow good histopathologic correlation with the human disease and reproducible colony-forming unit(s) (CFU) counts (Sheppard et al., 2004), inhalational models in hermetic chamber have been largely promoted for better recapitulating course of natural exposure (Andriole et al., 1992; Patterson, 2005), but they require a heavy inoculum (typically 12 mL of 10^9^ conidia/mL spread during 40 min before a 1h-subsequent exposure). Using a particle counter, it was evidenced that < 5% of generated particles were bigger than 5μm, which means that nebulization was likely efficacy enough to disperse *A. fumigatus* spores and avoid agglomerates (Leleu et al., 2013a,b). Tween and triton detergents have been commonly used to prepare fungal cell suspensions (Stephens-Romero et al., 2005). Some assays attempted to measure the amount of fungus that was actually inhaled by the challenged animals, and results were contradictory: Bretz et al. estimated the intake inoculum at 3.4 × 10^6^ in mouse lungs, when a 1.0 × 10^8^ *A. fumigatus* conidia/mL suspension was aerosolized during 90 s by pumping air into the flask using a 60 mL-syringe (Bretz et al., 2008). Ibrahim et al. estimated a mean inhalation of only 2.1 × 10^3^ conidia, when 1.2 × 10^10^ were nebulized in the chamber (Ibrahim et al., 2010, 2011). To improve the infection rate, Evans et al. suggested to supplement room air with 5% CO_2_ to promote maximal ventilation and homogeneous exposure throughout the lungs (Evans et al., 2010a,b). Some large-scale devices, like the Madison chamber, now offer the possibility to simultaneously deliver an accurate infectious inoculum by aerosol to an extensive number of rodents, but also to larger species (McMurray, 2001). Buskirk et al. described an interesting device based on an acoustical generator to chronically deliver dry fungal powder aerosols to mice housed in a nose-only exposure chamber. It works for 2 h twice a week during 1 month (Buskirk et al., 2014). Older inhalational processes, like the 1 min-forced exposure over *A. fumigatus* culture in flask, appear obsolete: although this insufflation technique recapitulates human infection faithfully since conidia are not solubilized in solutions that typically contain detergents (Stephens-Romero et al., 2005), it remains hard to control as animals were dying rapidly within 3–4 days following infectious challenge (Le Conte et al., 1992). Furthermore, the number of rodents that can be fitted into the specialized inhalation chamber is modest. Instead of the inhalation route, the intranasal deposition of *A. fumigatus* droplets close to the nares may be considered valuable, as theoretically allowing a more controllable intake by the rodents. Unfortunately through this procedure, about only 10% of the deposited fungal load was estimated to reach the lungs (Markaryan et al., 1994). Besides, evaluation by qPCR showed that the intranasal route actually generated smaller fungal burdens with higher standard deviation, and less homogenous pneumonia (Steinbach et al., 2004), and so fungal lesions were likely to arise in larger airways rather than in alveoli (Tang et al., 1993; Shibuya et al., 1999; Steinbach et al., 2004). Thus, some investigators suggested to introduce 5 μL saline serum into the nostrils with the objective of drawing out some of the spores which could possibly have been left in this area (García et al., 2006), and/or to place the rodents in semi-vertical position just after the experimental infection (Bakker-Woudenberg, 2003). Likewise, Lepak et al. recommended a pulmonary aspiration following the intranasal deposition in order to drive the spore suspension toward the lung alveoli (Lepak et al., 2013a,b,c). This procedure produced invasive aspergillosis in more than 90% of animals for which mortality was 100% by 72–96 h post-infection, when not treated. In such a context, the bronchial-tracheal instillation appears as a relevant alternative to inhalational and intranasal models, but so far it usually required a minor surgical procedure to expose the trachea for injection below a small incision (Bakker-Woudenberg, 2003; Clemons and Stevens, 2005; Goldman and Osmani, 2007). In order to enhance dispersion of fungal suspension into the lungs, some studies reported that rodents were mechanically ventilated following the bronchial-tracheal deposition, using a respirator for 2 min (Prüfer et al., 2014). Instead, alternative techniques that minimize surgery seem interesting, like oropharyngeal aspiration (Amarsaikhan et al., 2014; Shepardson et al., 2014; Sugui et al., 2014). Some investigators suggested a spore delivery into the caudal oropharynx of anesthetized rodents, in which normal breathing resulted in fluid aspiration into the lungs (Sugui et al., 2010, 2011, 2012, 2014; Faro-Trindade et al., 2012; Liu et al., 2013; Amarsaikhan et al., 2014; Lilly et al., 2014; O'Dea et al., 2014; Röhm et al., 2014; Shepardson et al., 2014). Animals were suspended by their upper incisors from a suture thread on a 90° inclined board, and their tongue was gently extended to prevent them to swallow during the experimental infection, then the chest was gently compressed and released just after deposition of liquid (Vethanayagam et al., 2011; Grimm et al., 2013). To better target the lungs, some authors described utility of visual guidance into the trachea (Rayamajhi et al., 2011). Original devices, like the MicroSprayer® aerosolizer, that generate a cloud of *A. fumigatus* spores directly into the trachea is even more valuable to get closer to the human disease. They do not require highly-seasoned personnel (Goldman and Osmani, 2007; Chandenier et al., 2009; Desoubeaux and Chandenier, 2012). In addition to the respiratory and the IV challenge, miscellaneous alternative routes of infection were developed to address some very specific forms of invasive aspergillosis, like cerebral aspergillosis (Chiller et al., 2002, 2003; Zimmerli et al., 2007) which is probably the most common extra-pulmonary site of infection in human medicine and that results in more than 80% mortality. Such models of intra-cranial inoculation do not have to receive systematic exogenous immunosuppression. Although this is not the natural route by which people usually acquire cerebral aspergillosis, the histopathological lesions and cellular host-response were very similar to the observations in human infection (Chiller et al., 2002), describing development of abscesses and necrotic areas in brain and subsequent infectious *foci* through bloodstream dissemination (Kleinschmidt-DeMasters, 2002). These models were primarily helpful to show the benefit of combination therapies, and also to evidence that higher dosages of an antifungal drug are not always more curative (Clemons et al., 2005, 2006b; Imai et al., 2005; Singh et al., 2005; Clemons and Stevens, 2006c).

This review noticed great variability regarding the name of *A. fumigatus* strain(s) to be used in experimental models. This choice has probable important consequences on the conclusions that were drawn from the assays, because host responses to individual strains likely differ in magnitude and in quality (Rizzetto et al., 2013): actually, no one could legitimately extend his finding to other works because each strain owns its particularities. However, the actual need to choose of a unique strain for all the rodent models is still a debatable issue today. For instance, one could imagine that selecting an hypo-virulent strain to induce lower mortality is more convenient to study diagnostic tools benefits at early stage and over the course of the disease, but in the other hand, one could address easily the overall survival in pre-clinical therapeutic assays, when mortality rates is almost 100% with an hyper-virulent strain. Globally, investigators always privileged the usage of referenced *A. fumigatus* strains that had been first isolated from clinical samples during invasive aspergillosis course in patients. For instance, AFB62 (Sugui et al., 2011, 2014; Losada et al., 2015), TIMM 2920 (Tansho et al., 2006), AF210 also named as NCPF 7101 or 2.06013 (Denning et al., 1997a,b; Verweij et al., 1998; Johnson et al., 2000; Paisley et al., 2005), IFM 4942 (Yamada et al., 1993), or BMU 01200 were isolated in hematopoietic stem cell transplants (Sun et al., 2012; Zhang et al., 2015), as well as A22 and AF65 (also referred to as NCPF 7097 or ATCC® MYA772 for the latter) were collected in lung biopsy from immunocompromised patients (Denning et al., 1997a; Verweij et al., 1998; Denning and Warn, 1999; Johnson et al., 2000; Paisley et al., 2005; Speth et al., 2013). Likewise, Zhao et al. relevantly used a *A. fumigatus* strain that came from fungal endophthalmitis for their work about eye aspergillosis (Zhao et al., 2015). In contrast, it was more questionable when were used *A. fumigatus* strains that had not been isolated in a context of invasive aspergillosis, like MF13 first found in a sputum secondary to aspergilloma (Mitsutake et al., 1995; Otsubo et al., 1998, 1999; Kakeya et al., 2008; Takazono et al., 2009) or CBS 100079 in a human ear (Sarfati et al., 2002). Likewise, Af285 had been isolated from the sputum of a patient suffering from allergic aspergillosis, but not invasive form (Madan et al., 2001, 2010; Kaur et al., 2007; Singh et al., 2009). Sometimes, the strains were derivative from the veterinary medicine, since they had been first isolated in chicken or pigeons (Van Cutsem et al., 1984). However, in the light of its historical widespread usage and as it was sequenced first (Nierman et al., 2005), AF293, also referred to as ATCC MYA-4609, FA/1153, FGSC A1100, CBS 101355, NCPF 7367, or IHEM18963, and its derivatives appears of course as the most standard strain, although it is also known to express less virulence than other ones. Dal/CEA10, also named CBS 144.89, D141, IHEM6963, NIH 4215, ATCC MYA-1163, FGSC A1163, or AF10, has also been largely used. It is regrettable that only few studies thoroughly addressed variations in virulence among distinct *A. fumigatus* strains (Hanson et al., 1995). Comparison with other *Aspergillus* species like *A. flavus, A. terreus, A. niger*, and *A. nidulans* should be even harder: great inter-species discrepancies are supposed, as it was preliminary illustrated through the *in vitro/in vivo* correlation that was satisfactory for amphotericin B in a murine model infected with *A. terreus*, but bad for both *A. flavus* and *A. fumigatus* (Johnson et al., 2000; Mosquera et al., 2001).

In rodent models, another concern of variability is the size of *A. fumigatus* inoculum to be used for the experimental challenge. It was evidenced a dose-dependent correlation regarding the number of conidia in the inoculum with the severity of infection, regardless of the rodent model and the *A. fumigatus* strain (Dixon et al., 1989; Hector et al., 1990; Chiller et al., 2002; Clemons et al., 2000a, 2002), but our bibliographic analysis highlighted very large variations, ranging for instance from 1.0 × 10^2^ (Waldorf et al., 1984) to 1.0 × 10^9^ (Graybill et al., 1998) for mice infected by intranasal route. In addition, culture conditions and pre-infection technical steps are also great challenges for harmonization. Incubation temperature and humidity, timing of culture in plate, diluent to be used, and method for conidia counting in the suspension are variable parameters that change a lot depending on the protocols. Such various practices may have some consequences: for example, one could propose the consequences on the *A. fumigatus* virulence of a long time culture-period vs. a short time culture-period.

Best methods for fungal burden assessment and outcome evaluation in infected rodents still remains controversial (Bowman et al., 2001; Balloy et al., 2005a; Imai et al., 2005; Singh et al., 2005). Of course, the straightforward parameter for disease progression remains the overall mortality, but ethical committees currently encourage using alternative endpoints. Easy to implement for most of the labs, *in vitro* mycological culture is a seducing semi-quantitative approach. Practically, homogenized tissues and centrifuged fluids are serially diluted and spread on agar plates, and thereafter number of CFU is counted on each after a specified incubation time. Culture was shown to be roughly indicative of fungal burden (Graybill et al., 1983), and only of viable fungus. It does not scale linearly with hyphal burden in infected tissues. Importantly, CFU count cannot discriminate between persistent and active infective lesions in tissues. Likewise, grinding the *A. fumigatus* hyphae during tissue pre-processing can produce artificially-increased CFU count [(Bowman et al., 2001; Kirkpatrick et al., 2002); unlike yeasts (te Dorsthorst et al., 2005), a large fungal mass of tangled hyphae cannot be distinguished from single-cell conidial forms when cultivated (Latgé, 1999)]. At the opposite, disruption of the organs can kill viable fungus, and thus leads to underestimation (Graybill, 2000). Therefore, for an enhanced reproducibility, it appears critical to adjust the CFU count to gram body weight or fluid volume. This unit is likely more appropriate than CFU *per* organ or total CFU *per* animal. By the way, it was surprising to note that Hummel et al. and Fidan et al. attempted to perform blood cultures (Hummel et al., 2004; Fidan et al., 2008), whereas it is well-known that *Aspergillus* genus never grows in blood culture bottles. In order to study disease progression and diagnosis (Becker et al., 2000; Loeffler et al., 2002), detection of GM antigen and β-D-glucans is probably useful to make correlation of animal data with clinical results. Mitsutake et al. underlined that the elevation in levels of β-D-glucans increased in correlation with the elevation of GM antigen titres, and thus is reliably measurable during experimental aspergillosis (Mitsutake et al., 1995). Detection in blood may provide multiple endpoints of assessment when repeated samplings are performed, especially in bigger species (Kirkpatrick et al., 2012; White et al., 2016). GM antigen and β-D-glucans may also be tested in BAL fluids (Jambunathan et al., 2013). Nevertheless, GM and β-D-glucans measurement globally remains quite expensive, and its interpretation is still difficult in rodents, since the positive cut-off were validated only in human samples so far (Becker et al., 2000). Consequently, further studies are requested to rule on the pre-clinical use of these surrogate biomarkers, and to determine standardized interpretive values and how their diagnostic results would be best used. As an alternative indicator of fungal burden, the chitin assay was tested several times, but it is old and not indicative whether the organism present is still viable, as it allows quantification of an inactive component of the cell wall deposited within infected tissues (Lehmann and White, 1975; Bowman et al., 2001; Balloy et al., 2005a). This method is also more labor-intensive than GM and β-D-glucans testing. Very sensitive, qPCR using the 18S rRNA gene as target has been more recently applied to the determination of *A. fumigatus* burden in the tissues, but it requires specialized costly equipment and reagents that labs with limited resources can't afford. qPCR seems correlated to CFU count (Bowman et al., 2001; Loeffler et al., 2002; Singh et al., 2005; Lengerova et al., 2012), although some investigators reported that it is less suitable than the latter to confirm therapeutic effects of antifungal drugs. Indeed, qPCR is too much subtle to detect actual changes in fungal load, while mycological culture is able to indicate the presence of a limited amount of residual organism (Singh et al., 2005). Furthermore, qPCR cannot provide information about the viability of the fungal elements, especially because no one really knows the clearance timing of DNA from dead *A. fumigatus* (Vallor et al., 2008; Lengerova et al., 2012). In this context, histopathology is still considered as the reference standard to prove infection (Goldman and Osmani, 2007; Desoubeaux et al., 2014a). In addition to the observation of fungal elements, slides examination provides greatly detailed insights about the inflammation process and the extent of infection. Nonetheless, one should notice the overall lack of technical details that are provided in publications for the correct achievement of slides preparation and for a complete assessment of tissue invasion. For instance for histopathology in lungs, only a few authors thoroughly described how they processed: Becker et al. specified that every lung has to be cut at three levels ±1 mm apart. According to their recommendations, two adjacent sections were obtained at every level: one should be stained with haematoxylin and eosin and the other with Grocott-Gomori's methenamine silver (Becker et al., 2003). Baistrocchi et al. recommended to perform serial step sections of 5 μm, taken at 80 μm intervals and stained with Periodic acid-Schiff: a minimum of five sections of each lung has to be examined for all animals in each experiment in order to ensure 100 lesions were detected in the group displaying the highest level of infection (Baistrocchi et al., 2016). Additional information were also provided by Panepinto et al. when they explained how histopathological lesion areas were measured by using ScionImage® analysis software (Panepinto et al., 2003). Among the unusual miscellaneous methods for monitoring the course of infection, some investigators reported how useful could be *in vivo* imaging techniques based on bioluminescent *A. fumigatus* strain (Brock et al., 2008; Ibrahim-Granet et al., 2010; Fekkar et al., 2012; Jouvion et al., 2012), or antibody-guided positron emission tomography and magnetic resonance imaging (Rolle et al., 2016). The former requires luciferin as exogenous substrate, while the latter needs particular caution for radiation protection. Recently, the expanding availability of immunological reagents to monitor the recruitment and functional activation of immune cells informs immune-pathophysiology studies aiming to identify the molecular and cellular basis of antifungal immunity. ELISA assays measure the production of cytokines and other inflammatory mediators, while flow cytometry can quantify host leukocyte populations that reside in or are recruited to portals of infection. Interestingly, other authors made specific focus on clinical endpoint scales that are far less expensive to measure, and allow refinement and reducing euthanasia procedures: modifications of the respiratory function during pulmonary aspergillosis (Becker et al., 2006), body temperatures changes (Adamson et al., 2013), or behavior alteration and weight loss ≥20% baseline (Chandenier et al., 2009; Desoubeaux and Chandenier, 2012). All these surrogate endpoints mandated by ethic committees need to be clearly clarified and defined on quantitative terms whenever possible, to decide a correct timing for euthanasia (Morton and Griffiths, 1985; Carstens and Moberg, 2000). For example, a validated grid evaluates twice daily the discomfort level for each animal according to a scale which scores from 1 to 6 on the basis of appearance and physiological behavior changes (Becker et al., 2006; Chandenier et al., 2009; Desoubeaux and Chandenier, 2012), reaction to stimuli, and other readily-available parameters (Adamson et al., 2013): empirically as an example, score 1, no discomfort; score 2, minor discomfort; score 3, poor discomfort; score 4, serious discomfort; score 5, severe discomfort; score 6, death (Morton and Griffiths, 1985; Chandenier et al., 2009; Desoubeaux et al., 2014b).

**Figure 4:**
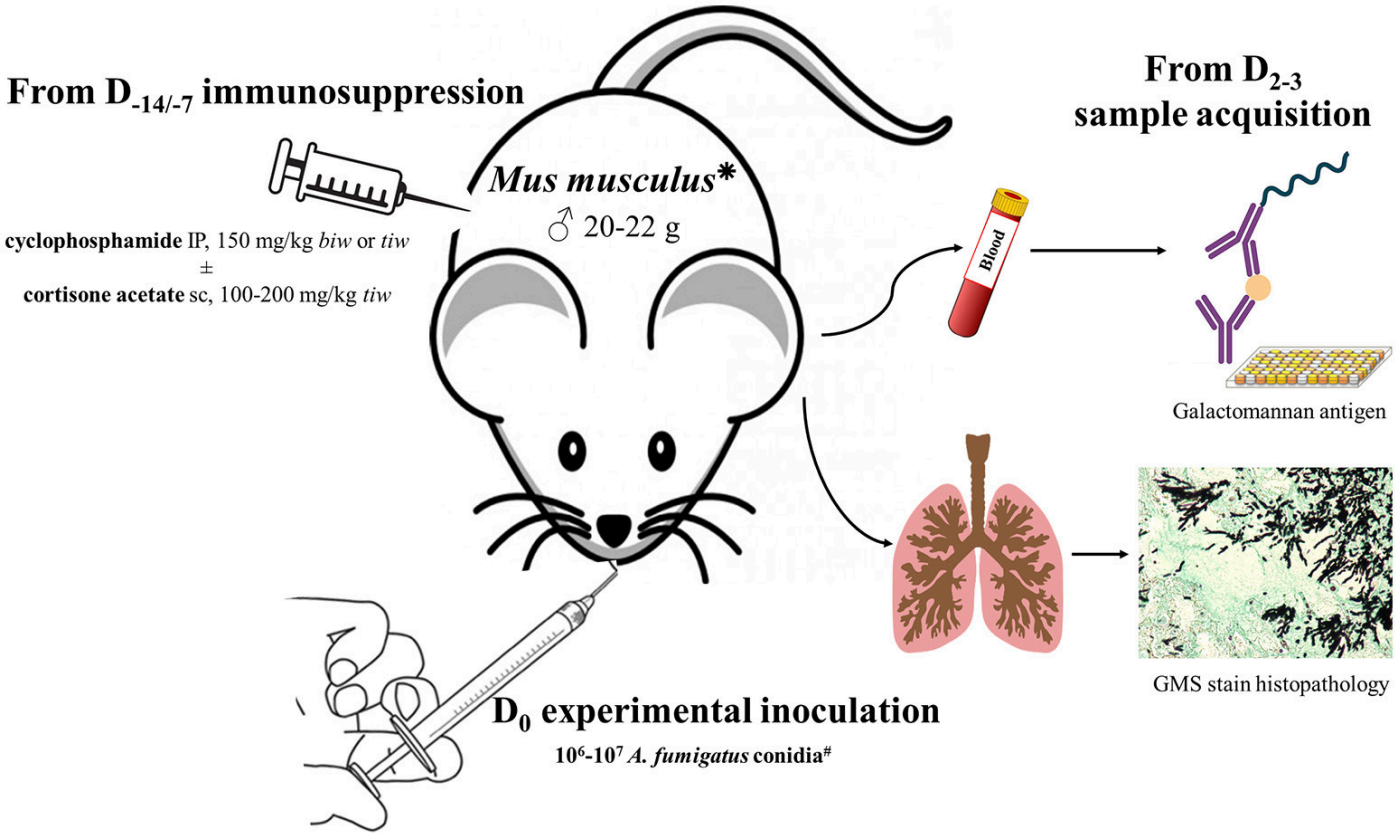
**Summary of recommendations for further studies involving animal models of invasive aspergillosis**. Suggestions for standardization are based on objective analysis of all the published literature faced with the authors' personal experience. In light of all the benefits due to their small size, their costs and the large availability of reagents dedicated to, mice should be privileged. Within a 1–2 week-long period, immunocompromised status is easily achieved by repeated injections of low-cost drugs, like alkylating agents or steroids. Thereafter, a tracheo-pulmonary challenge is recommended by non-invasive device, like MicroSprayer® aerosolizer, allowing accurate control of the fungal inoculum. In such a context, no more than 10^7^ *A. fumigatus* conidia are usually needed to yield 90–100% infection rates. Generally, onset of clinical signs occurs within 48–72 h after the experimental inoculation. After that time, the challenged animals start becoming moribund from aspergillosis (ruffled fur, decreased defecation, lethargy, anorexia, weight loss, ataxia, various pulmonary signs, gross bleeding …). Although death remains the major clinical outcome, primarily in therapeutic assays, alternative endpoints may be assessed to estimate the fungal load while refining the animal welfare: nowadays, galactomannan antigen determination in blood and histopathology in lungs appear reliable and largely validated, in comparison with other surrogate biomarkers. ^*^For therapeutic assays: outbred mouse strains like Albino Swiss Webster and CD-1; for pharmacology-pharmacokinetics and toxicology studies: outbred mouse strains like Swiss OF1 and NMRI; for immuno-pathophysiology: inbred mouse strains like C57BL/6, BALB/c, DBA2, 129/Sv, and CD2F1; for general purposes: C57BL/6 and BALB/c. ^#^Referenced *A. fumigatus* strains should be privileged for inoculation, especially those that have been widely used so far, like AF293 and Dal/CEA10. Otherwise, it makes sense to use strains that were initially isolated in a relevant context of invasive aspergillosis. Environmental strains or local unreferenced strains should be avoided, because they don't allow large-scale reliable comparison. *biw*, Twice a week; D, Day (D_0_ being the date of experimental challenge); GMS, Grocott-Gomori methenamine silver staining; IP, Intraperitoneal; ♂, Male; sc, Subcutanous; *tiw*, Thrice a week.

The richness of experience accumulated over time for rodent models of invasive aspergillosis has demonstrated its utility for reliably reflecting what happens clinically in humans, when for instance addressing its immuno-pathophysiology, predicting clinical efficacy, pharmacology and toxicity of antifungal drugs, and studying diagnostic predictive value of the biomarkers. Unfortunately, it appeared in this exhaustive review that there is obviously no consensus to develop the ideal model of aspergillosis. Indeed as it is also the case for other models of infectious diseases, no single model of invasive aspergillosis is currently able to answer all questions, and each has its own limitations. Besides, we have shown that distinct rodent models can provide different—and even contradictory—results, depending on the context for which they are developed. For this reason, substantial variability from one experiment to the next has to be largely minimized in a near future, firstly by defining the minimum criteria to ensure for a reliable and reproducible support. According to us, and as it is the most consensual, usage of male mice immunocompromised by both alkylating drug and steroids, and challenged through respiratory airways in inhalation chamber, by deposition into the nares, or by a non-invasive device, seems the most relevant (Figure 4). Rats remain good alternative for tracheal inoculation. This attempt at is a preliminary critical step to tend toward standardization. It aims to subsequently allow high-quality *in vivo* experiments and direct comparisons of data between centers, especially since the scientific community working on *A. fumigatus* infection continues to grow over time.

## Author contributions

GD designed and performed the bibliographic analysis. GD drafted the manuscript and the figures. CC reviewed the manuscript.

## Funding

This work was supported by none. The authors did not receive any specific research funding for this study.

### Conflict of interest statement

The authors declare that the research was conducted in the absence of any commercial or financial relationships that could be construed as a potential conflict of interest.
