# Supplementary material for: Rodent Models of Invasive Aspergillosis due to Aspergillus fumigatus: Still a Long Path toward Standardization
Source: Front Microbiol. 2017 May 16;8:841. doi: 10.3389/fmicb.2017.00841 (PMC5432554; doi:10.3389/fmicb.2017.00841)
Supplement: Supplementary file 1 [file Table1.docx]

**Supplementary material 1: Listing of the publications using animal models of invasive aspergillosis which address simultaneously several topics or that involve both males and females.** References of articles that address very specific questions, other than those listed below, are directly mentioned in the text.

| Diagnosis/imaging and pre-clinical therapy | (Arrese et al., 1994; Bowman et al., 2001; Brock et al., 2008; Cutsem et al., 1993; Galiger et al., 2013; Lass-Flörl et al., 2003; Overdijk et al., 1996; Petrik et al., 2012; Scotter and Chambers, 2005; Severin et al., 2015; Yang et al., 2009b) |
| --- | --- |
| Diagnosis/imaging and immuno-pathophysiology/virulence | (Hasenberg et al., 2011; Ibrahim-Granet et al., 2010; Jensen et al., 1993b, 1996a, 1996b; Jhingran et al., 2012; Jouvion et al., 2012; Lin et al., 2012; Lionakis et al., 2005; Losada et al., 2015; Overdijk et al., 1996, 1999; Raghuwanshi et al., 2005; Rammaert et al., 2015) |
| Immuno-pathophysiology/virulence and pre-clinical therapy | (Abad-Diaz-De-Cerio et al., 2013; Ahmad et al., 1995; Arendrup et al., 2008, 2008, 2010; Bellocchio et al., 2005; Ben-Ami et al., 2013; BitMansour et al., 2005; Chaturvedi et al., 2005; Clemons et al., 2014b, 2014a, Dannaoui et al., 1999, 2001, Denning et al., 1997a, 1997b; Dhuley, 1998; Gaziano et al., 2004; Graybill et al., 1998a, 1998b; Ito et al., 2006; Ito and Lyons, 2002; Khan et al., 2004, 2005; Lamoth et al., 2014a; Leal et al., 2013; Lewis et al., 2011b; Li et al., 2015b; Lo Giudice et al., 2010, 2012; Lohan et al., 2015; Manavathu et al., 2005; Marra et al., 2014; Moretti et al., 2012; Nagai et al., 1995; Naik et al., 2011; Nobre, 1977; Odds et al., 1998; Okamoto et al., 2004; Overdijk et al., 1996; Polak-Wyss, 1991; Quezada et al., 2008; Shibata et al., 2014; Singh et al., 2009; Tandon et al., 1988; Torosantucci et al., 2005; Van Epps et al., 2003; Verweij et al., 2008; Verwer et al., 2013) |
| Simultaneous usage of both male and female rodents | (Caffrey et al., 2015; Caretti et al., 2016; Carrion et al., 2013; Cenci et al., 1998, 2000; Clark et al., 2016; Cornish et al., 2008; Dabur et al., 2005; Defaveri et al., 1990; Dinauer et al., 2001; Espinosa et al., 2014; Grahl et al., 2011; Graybill et al., 1983; Graybill and Kaster, 1984; Grimm et al., 2013; Hartigan et al., 2010; Jhingran et al., 2012; Li et al., 2011; Lohan et al., 2015; Morton et al., 2012; Park et al., 2010; Petrik et al., 2012; Ramirez-Ortiz et al., 2011; Rivera et al., 2006; Röhm et al., 2014; Stojanovic et al., 2011; Taylor et al., 2014b; Van Cutsem et al., 1984; Vethanayagam et al., 2011; Wang et al., 2014a; Williams et al., 1981; Zhong et al., 2009). |

**Supplementary material 2: Listing of the publications which report usage of main rodent strains in animal models of invasive aspergillosis.** References of articles that address very specific questions about rodent species and strains are directly mentioned in the text.

|  | Mouse models | Rat models | Guinea pig models |
| --- | --- | --- | --- |
| Outbred strains | CD-1 (Amich et al., 2013; Armstrong-James et al., 2009; Arroyo et al., 1977; Aufauvre-Brown et al., 1997, 1998; Barchiesi et al., 2016; Beaulieu et al., 1993; Behnsen et al., 2010; Bergmann et al., 2009; Bertuzzi et al., 2014; Blatzer et al., 2011; Brown et al., 2000; Centeno-Lima et al., 2002; Chiller et al., 2002, 2003, Chowdhry et al., 1996, 1997; Chung et al., 2014; Clemons et al., 2002, 2005, 2006, 2012, 2014a; Clemons and Stevens, 2004; Cramer et al., 2008; de Gouvêa et al., 2008; Denning et al., 1995, 1997b, 1997a; Denning and Stevens, 1991; Denning and Warn, 1999; García et al., 2006; Grahl et al., 2011, 2012; Han et al., 2010; Hanson et al., 1995; Hartmann et al., 2011; Hensel et al., 1998; High and Washburn, 1997; Howard et al., 2011; Hu et al., 2007; Huber and Bignell, 2014; Ichikawa et al., 2001; Imai et al., 2004, 2005; Italia et al., 2011; Jiang et al., 2008; Johnson et al., 2000; Juvvadi et al., 2013; Keller et al., 2011; Kotz et al., 2010; Krappmann et al., 2004; Lamoth et al., 2014a, 2014b; Li et al., 2011; Liu et al., 2011, 2012; Luque et al., 2003; Macheleidt et al., 2015; Martinez et al., 2000; Mavridou et al., 2010b, 2010a; Mazzolla et al., 1991; McDonagh et al., 2008; Melchers et al., 1994; Mellado et al., 1996; Morton et al., 2011; Mouton et al., 2009; Mouyna et al., 2010; Muszkieta et al., 2014a; Oakley et al., 1997; O’Hanlon et al., 2011; Ohtsuka et al., 1997; Olivas et al., 2008; Oliver et al., 2012; Paisley et al., 2005; Pinchai et al., 2009, 2010; Puttikamonkul et al., 2010; Rajendran et al., 2011; Renshaw et al., 2016; Ruiz-Cabello et al., 2002; Sasse et al., 2008; Schöbel et al., 2010; Schrettl et al., 2004, 2007, Seyedmousavi et al., 2013a, 2013c, 2013b, 2014, 2015b, 2015a; Shepardson et al., 2014; Sirivoranankul et al., 2014; Slesiona et al., 2012; Smith et al., 1993, 1994; Spreadbury et al., 1993; Spreghini et al., 2009; Steinbach et al., 2004, 2006; Takasuka et al., 1999; Tanaka et al., 2015; Tang et al., 1993; te Dorsthorst et al., 2005; Vargas-Muñiz et al., 2015; Verweij et al., 1998, 2008; Wagener et al., 2008; Waldorf et al., 1984; Waldorf and Diamond, 1985; Warn et al., 2003, 2006, 2010; Wartenberg et al., 2011; Willger et al., 2008, 2012; Wong Sak Hoi et al., 2011; Yasmin et al., 2012);  Albino Swiss Webster (Baisakh et al., 1975; Beauvais et al., 2005, 2007; Clark et al., 1991; Desai and Naik, 2009; Dixon, 1987; Dixon et al., 1989; Ghosh et al., 1977; Hector et al., 1990; Jahn et al., 1997; Langfelder et al., 1998; Lehmann and White, 1975, 1976, 1978, Lewis et al., 2002, 2005; Lionakis et al., 2005; Lohan et al., 2015; Lupetti et al., 2002; Maheshwari et al., 1988; Maubon et al., 2006; Mouyna et al., 2010; Odds et al., 1998; Olson et al., 2010, 2015; Paris et al., 1993; Paulussen et al., 2015; Polak, 1982, 1987; Polak et al., 1982; Polak-Wyss, 1991; Quezada et al., 2008; Raghuwanshi et al., 2005; Saeed and Hay, 1981; Sandhu et al., 1970, 1976; Sarfati et al., 2002; Siaens et al., 2004; Singh et al., 2014; Smith, 1972, 1973; Tandon et al., 1988; Thau et al., 1994; Van Cutsem et al., 1984, 1987; White, 1977; Wiederhold et al., 2004, 2006);  Swiss OF1 (Dannaoui et al., 1999, 2001; D’Enfert et al., 1996; Dubourdeau et al., 2006; Fekkar et al., 2012; Graybill et al., 1998a; Ibrahim-Granet et al., 2008; Jaton-Ogay et al., 1994; Lamarre et al., 2007; Lambou et al., 2010; Langfelder et al., 2001; Le Conte et al., 1992; Mondon et al., 1996; Monod et al., 1993; Moutaouakil et al., 1993; Muszkieta et al., 2014a; Philippe et al., 2003; Rammaert et al., 2015; Salas et al., 2013; Schöbel et al., 2007; Schrettl et al., 2010b);  NMRI (Arendrup et al., 2008, 2010, 2010; Reichard et al., 1997; Schaffner and Frick, 1985; Schaude et al., 1990; Walzl et al., 1987) | RP-strain (Becker et al., 2000, 2002a, 2002b, 2003, 2006; Dams et al., 1999; Leenders et al., 1996; Ruijgrok et al., 2001, 2005, 2006, van de Sande et al., 2008, 2009; Van Etten et al., 2000; van Vianen et al., 2006; Verwer et al., 2013);  Lewis (Petrik et al., 2010, 2012, 2014);  Dark Agouti (El-Muzghi et al., 2013; Mirkov et al., 2014, 2015);  Albino Oxford (Mirkov et al., 2014, 2015); | Pirbright white Dunkin-Hartley (Arrese et al., 1994; Chandrasekar et al., 2004; Cutsem et al., 1993; Dufresne et al., 2012; Hooper et al., 2012; Jambunathan et al., 2013; Kirkpatrick et al., 2000, 2002b, 2002a, 2006, 2012; Lengerova et al., 2012; Martin et al., 1997; Odds et al., 1998, 2000; Vallor et al., 2008; Van Cutsem et al., 1989, 1990; White et al., 2016; Wiederhold et al., 2009, 2013, 2015) |
| Inbred strains | BALB/c (Abad-Diaz-De-Cerio et al., 2013; Ahmad et al., 1989b, 1989a, 1990a, 1990b, 1995; Al-Bader et al., 2010; Allendoerfer et al., 1995; Alvarez et al., 2007; Amarsaikhan et al., 2014; Amich et al., 2013, 2016; Arber et al., 2005; Baistrocchi et al., 2016; Bellocchio et al., 2005; Ben-Ami et al., 2009, 2010b, 2010a, 2013; Ben-Ami and Kontoyiannis, 2012; Bhatia et al., 2011, 20; Bom et al., 2015; Bonnett et al., 2006; Bozza et al., 2002a, 2002b, 2003, 2009; Brock et al., 2008; Buskirk et al., 2014; Cenci et al., 1997, 1999, 2000, Chakraborty and Naik, 2000, 2003; Chang et al., 2004; Chaturvedi et al., 2005; Chiang et al., 2006, 2008; Cunha et al., 2010; da Silva Ferreira et al., 2006, 2007; Dabur et al., 2005; Davies and Pope, 1978; de Castro et al., 2014b, 2014a; de Repentigny et al., 1993; Defaveri et al., 1990; Delmas et al., 2002; Dhuley, 1998; Dinamarco et al., 2012b, 2012a; Dirr et al., 2010; Donat et al., 2012; Du et al., 2002; Duong et al., 1998; Durrant et al., 2011; Ejzykowicz et al., 2009, 2010, Evans et al., 2010a, 2010b; Falk et al., 2004; Faro-Trindade et al., 2012; Fei et al., 2011; Fidan et al., 2008; Fleck and Brock, 2010; Frosco et al., 1994; Galiger et al., 2013; Gaziano et al., 2004; Gehrke et al., 2010; Gravelat et al., 2008, 2010, 2013; Graybill et al., 1983; Graybill and Kaster, 1984; Gresnigt et al., 2014; Harindran et al., 1999; Heesemann et al., 2011; Hein et al., 2015; Herbst et al., 2013; Hissen et al., 2005; Hood et al., 2010; Hummel et al., 2004; Ibrahim et al., 2010, 2011; Ibrahim-Granet et al., 2010; Jensen et al., 1993a, 1996b, Jensen and Hau, 1990a, 1990b, Jensen and Schønheyder, 1989, 1993; Jiang et al., 2014; Johnson et al., 2005; Jouvion et al., 2012; Kaur et al., 2007; Keller et al., 2011; Khalaj et al., 2012; Khan et al., 2004, 2005, 2006; Kolattukudy et al., 1993; Kretschmar et al., 2001; Kupfahl et al., 2006; Lass-Flörl et al., 2003; Lee and Kolattukudy, 1995; Leleu et al., 2013a, 2013b, 201; Lessing et al., 2007; Lewis et al., 2007, 2008b, 2011a, 2011b, 2014, Li et al., 2007, 2008, 2012, 20, 2015b, Liebmann et al., 2004b, 2004a; Lilly et al., 2014; Lin et al., 2010, 2012, Liu et al., 2010, 2011; Loeffler et al., 2002; Losada et al., 2015; Ma et al., 2008; Madan et al., 2001; Martinez et al., 2013; Mehrad et al., 1999a; Mirkov et al., 2011, 2013; Monroy and Sheppard, 2005; Montagnoli et al., 2003, 2006, Moonis et al., 1992, 1993b, 1993a, 1994, Moretti et al., 2008, 2012; Morrison et al., 2003; Mota Júnior et al., 2008; Nagai et al., 1995; Naik et al., 2011; Nawada et al., 1996; O’Dea et al., 2014; Otsubo et al., 1998; Owais et al., 1993, 199; Poelmans et al., 2016; Pongpom et al., 2015; Pope and Davies, 1979; Qiao et al., 2008; Ramaprakash et al., 2009; Rebong et al., 2011; Ren et al., 2010; Rieber et al., 2015; Rieg et al., 2006; Romani et al., 2004, 2006; Savers et al., 2016; Saxena et al., 1998; Saxena and Ghosh, 2000; Schaffner et al., 1982; Schmalhorst et al., 2008; Schrettl et al., 2010a; Shao et al., 2005b, 2005a, Sheppard et al., 2004, 2006b, 2006a; Shirkhani et al., 2015; Singh et al., 2009; Slesiona et al., 2012; Sohrabi et al., 2010; Speth et al., 2013; Stephens-Romero et al., 2005; Stuehler et al., 2011; Sugareva et al., 2006; Sugui et al., 2007b, 2011, 2014; Svirshchevskaya et al., 2009; Templeton et al., 2011; Valiante et al., 2008; Wang et al., 2013, 2014b, 2014a, 2015; Williams et al., 1981; Winkelströter et al., 2015; Xu et al., 2009; Yang et al., 2009a, 2009b, 2010; Zaas et al., 2008; Zelante et al., 2007, 2009, Zhang et al., 2005, 2008a, 2008b, 2013a; Zhong et al., 2009; Zhou et al., 2007);  C57BL/6 (Aimanianda et al., 2009; Aratani et al., 2000; Arber et al., 2005; Balloy et al., 2005b; Bedke et al., 2014; Bellocchio et al., 2004a, 2004b; Benjamim et al., 2003; BitMansour et al., 2002, 2005; BitMansour and Brown, 2002; Bonifazi et al., 2010; Bonnett et al., 2006; Bozza et al., 2003, 2008, 2009, 2014; Bretz et al., 2008; Bruns et al., 2010; Caffrey et al., 2015; Caretti et al., 2016; Carpenter and Hogaboam, 2005; Carrion et al., 2013; Carvalho et al., 2012; Cenci et al., 1997, 2001; Chai et al., 2011; Chaudhary et al., 2012; Chiller et al., 2002; Clark et al., 2016; Clemons et al., 2000a, 2010, 2014b; Cohen et al., 2011; Cunha et al., 2010; de Luca et al., 2010; De Luca et al., 2012; Dubourdeau et al., 2006; Dufresne et al., 2012; Durrant et al., 2011; Espinosa et al., 2014; Fontaine et al., 2011; Garlanda et al., 2002; Gessner et al., 2012, 2013; Hartigan et al., 2010; Hasenberg et al., 2011; Herbst et al., 2013, 2015; Hohl et al., 2005; Hsu et al., 2013; Huang et al., 2014; Iannitti et al., 2013; Jhingran et al., 2012, 2015; Jiang et al., 2015; Kapp et al., 2014; Karki et al., 2015; Kasahara et al., 2016; Kerr et al., 2016, 201; Leal et al., 2010, 2012, 2013; Li et al., 2014; Liu et al., 2011; Loussert et al., 2010; Madan et al., 2010; Marples et al., 2011; Mattila et al., 2008; Mazaki et al., 2006; McCormick et al., 2012; Mehrad et al., 1999a, 2000; Mircescu et al., 2009; Mirkov et al., 2010, 2011, 2012, 2013, Moretti et al., 2008, 2012, 2014a; Morton et al., 2012; Pardo et al., 2006; Park et al., 2009, 2010; Phadke et al., 2007; Ramirez-Ortiz et al., 2011; Ren et al., 2010; Richie et al., 2007a, 2009, Rivera et al., 2006, 2009, 2011; Rizzetto et al., 2013; Rodriguez et al., 2007; Rolle et al., 2016; Romani et al., 2004, 2006; Rubino et al., 2012; Savers et al., 2016; Schlitzer et al., 2013; Schütte et al., 2009; Severin et al., 2015; Shepardson et al., 2013; Shibata et al., 2014; Shirkhani et al., 2015; Steele et al., 2005; Stephens-Romero et al., 2005; Stojanovic et al., 2011; Stuehler et al., 2011; Sun et al., 2012; Svirshchevskaya et al., 2009; Taylor et al., 2014b, 2014a; Vallon-Eberhard et al., 2008; Van Epps et al., 2003; Wagener et al., 2008; Werner et al., 2011; Wharton et al., 2015; Zaas et al., 2008; Zelante et al., 2007, 2009, 2012, 2015)(Beauvais et al., 2013; Björgvinsdóttir et al., 1997; Bonnett et al., 2006; Cornish et al., 2008; Cruz et al., 2013; D’Angelo et al., 2009; De Luca et al., 2012; de Luca et al., 2014; Dennis et al., 2006; Dinauer et al., 2001; Espinosa et al., 2014; Grimm et al., 2013; Kasahara et al., 2016; Leal et al., 2013; Liu et al., 2013; Morgenstern et al., 1997; Morton et al., 2010; Petersen et al., 2002; Philippe et al., 2003; Pollock et al., 1995; Prüfer et al., 2014; Ren et al., 2010; Röhm et al., 2014; Stein et al., 2013; Vethanayagam et al., 2011; Wharton et al., 2015);  ICR/HaJ (Abruzzo et al., 2000; Allen et al., 1994; Appel et al., 2010; Arnusch et al., 2012; Bok et al., 2005, 2006; Cramer et al., 2006; Dagenais et al., 2008; Fujie et al., 2000b, 2000a; Furumai et al., 1993, 199; Graybill et al., 1998a, 2003; Graybill and Ahrens, 1985; Gyotoku et al., 2012; Hanazawa et al., 2000; Hata et al., 1996b, 1996a; Ikeda et al., 2000; Jung et al., 2009; Krishnan Natesan et al., 2012; Lee et al., 2009; Lepak et al., 2013b, 2013a, 2013c; Levdansky et al., 2010; Matsumoto et al., 2000; Mellado et al., 2005, 2011, Mitsuyama et al., 2003, 2008; Nagasaki et al., 2009; Okazaki et al., 2009; Oki et al., 1992; Romano et al., 2006; Schaffner and Böhler, 1993; Sekonyela et al., 2013; Shadkchan et al., 2004; Sharon et al., 2009; Sionov et al., 2005, 2006, Sionov and Segal, 2003, 2004; Steinbach et al., 2006; Takazono et al., 2009; Tansho et al., 2006; Tolman et al., 2009; Tsitsigiannis et al., 2005; Vaknin et al., 2014; Wiederhold et al., 2008; Yamada et al., 1993, 1; Yamakami et al., 1996; Yonezawa et al., 2000; Zhang et al., 2015);  DBA/2J (Abruzzo et al., 1995, 1997; Bowman et al., 2001; Cenci et al., 1997, 1998, 1999, 2002; Chiller et al., 2002; Clemons et al., 2000b, 2011; Clemons and Stevens, 2006; Del Sero et al., 1999; Durrant et al., 2011; Hata et al., 2011; Hector et al., 1990; Hector and Yee, 1990; Kai et al., 2013; Kakeya et al., 2008; Liu et al., 2011; Manavathu et al., 1998, 2005, Olson et al., 2006, 2008; Steinbach et al., 2006; Stephens-Romero et al., 2005; Svirshchevskaya et al., 2009; Zaas et al., 2008);  CF-1 (Adamson et al., 2013; Bennett et al., 2006; Brieland et al., 2001; Cacciapuoti et al., 2000, 2006; Feng et al., 2011; Ford and Friedman, 1967; Fortwendel et al., 2005; Fuller et al., 2011; Ito et al., 2006; Ito and Lyons, 2002; Kothary et al., 1984, 1; Krishnan et al., 2013; Panepinto et al., 2003; Patera et al., 2004; Plempel, 1984; Powers-Fletcher et al., 2011; Richie et al., 2007b, 2009, 2011; Schaffner et al., 1982; Wallace et al., 1997; Zhao et al., 2006);  Albino DDY (Adamson et al., 2013; Bennett et al., 2006; Brieland et al., 2001; Cacciapuoti et al., 2000, 2006; Feng et al., 2011; Ford and Friedman, 1967; Fortwendel et al., 2005; Fuller et al., 2011; Ito et al., 2006; Ito and Lyons, 2002; Kothary et al., 1984, 1; Krishnan et al., 2013; Panepinto et al., 2003; Patera et al., 2004; Plempel, 1984; Powers-Fletcher et al., 2011; Richie et al., 2007b, 2009, 2011; Schaffner et al., 1982; Wallace et al., 1997; Zhao et al., 2006);  129/Sv (Björgvinsdóttir et al., 1997; Dinauer et al., 2001; Faro-Trindade et al., 2012; Gao et al., 1997; Garlanda et al., 2002; Gessner et al., 2012; Losada et al., 2015; Pardo et al., 2006; Sugui et al., 2007a, 2007b; Troke et al., 1987; Werner et al., 2009; Zaas et al., 2008); | Sprague-Dawley (Alsaadi et al., 2012; Bartroli et al., 1998a, 1998b; Chandenier et al., 2009; Cicogna et al., 1997; Desoubeaux et al., 2014; Desoubeaux and Chandenier, 2012; García et al., 2002; Habicht et al., 2002; Hachem et al., 2006; Hanazawa et al., 2000; Hashiguchi et al., 1994; Hashimoto et al., 1998; Kurtz et al., 1995; Lin et al., 2014; Lo Giudice et al., 2010; Marra et al., 2014; Mitsutake et al., 1995; Miyazaki et al., 1993; Morisse et al., 2012, 2013; Murphy et al., 1997; Najvar et al., 1995; Niki et al., 1991; Niwano et al., 1999; Otsubo et al., 1999; Paris et al., 2003; Risovic et al., 2007; Schmitt et al., 1988, 1990, 1991, 1992; Scotter and Chambers, 2005; Shibuya et al., 1999; Sivak et al., 2004b, 2004a, Wasan et al., 2007, 2009; Wong et al., 1989; Yan et al., 2014; Yu et al., 1990; Zhang et al., 2014; Zhao et al., 2010; Zhao and Perlin, 2013); albino Wistar (Ahmad et al., 2014; Gavaldà et al., 2005; Guo et al., 2012; Jie Zhao et al., 2009; Khan et al., 2008; Land et al., 1989; Li et al., 2015a; Martín et al., 2003; Turner et al., 1975a, 1976; Xu et al., 2015; Zhang et al., 2013b; Zimmerli et al., 2007); albino-CD (Ullmann et al., 2007) |  |
| Hybrid strains | CD2F1 (Cenci et al., 1997; Hayashi et al., 2002; Torosantucci et al., 2005; Tsuchimori et al., 2002; Vecchiarelli et al., 1988);  B6.129S2 (Bozza et al., 2008; Cenci et al., 1997; Dennis et al., 2006; Grahl et al., 2011, 2012; Hayashi et al., 2002; Jiang et al., 2013; Leal et al., 2012; Sugui et al., 2010, 2011, 2012, 2014; Torosantucci et al., 2005; Tsuchimori et al., 2002; Vecchiarelli et al., 1988; Werner et al., 2011; Willger et al., 2008) |  |  |

**Supplementary material 3: Listing of the publications which reports usage of main alternative immunosuppressive regimens in animal models of invasive aspergillosis.** References that focus on very specific immunosuppressive regimens are cited in the text.

| anti-neutrophil Ly6 (Gr-1) antibody | (Adamson et al., 2013; Amarsaikhan et al., 2014; Beauvais et al., 2013; Bedke et al., 2014; Bonifazi et al., 2010; Bruns et al., 2010; Carpenter and Hogaboam, 2005; Carvalho et al., 2012; Cenci et al., 1998, 1999, 2002; Corbel and Eades, 1977; Cruz et al., 2013; de Luca et al., 2010; Del Sero et al., 1999; Fei et al., 2011; Hartigan et al., 2009, 2010; Iannitti et al., 2016; Ibrahim-Granet et al., 2010; Kapp et al., 2014; Leal et al., 2012; Maheshwari et al., 1988; Mehrad et al., 1999a, 2000, 2002; Montagnoli et al., 2006; Morrison et al., 2003; Nagai et al., 1995; Park et al., 2009, 2010; Phadke et al., 2007; Ramaprakash et al., 2009; Richie et al., 2007a, 2009; Rivera et al., 2005; Rodriguez et al., 2007; Rolle et al., 2016; Severin et al., 2015; Shibata et al., 2014; Stephens-Romero et al., 2005; Tandon et al., 1988; Wharton et al., 2015; Zelante et al., 2007) |
| --- | --- |
| Body irradiation | (Arber et al., 2005; Beaulieu et al., 1993; Bellocchio et al., 2005; BitMansour et al., 2002; BitMansour and Brown, 2002; Björgvinsdóttir et al., 1997; Bozza et al., 2003, 2009; Clark et al., 2016; Dinauer et al., 2001; Gaziano et al., 2004; Hartigan et al., 2010; Jhingran et al., 2012; Kolattukudy et al., 1993; Lee and Kolattukudy, 1995; Markaryan et al., 1994; Moretti et al., 2012, 2014a; Philippe et al., 2003; Schlitzer et al., 2013; Sutton et al., 1996) |
| Genetic mutation | cytokines, receptors, proteins, enzymes associated with immune response (Aratani et al., 2000; Balloy et al., 2005b; Bellocchio et al., 2004a, 2004b; Bonifazi et al., 2010; Bonnett et al., 2006; Bozza et al., 2008, 2014; Bretz et al., 2008; Caffrey et al., 2015; Carpenter and Hogaboam, 2005; Carrion et al., 2013; Carvalho et al., 2012; Cenci et al., 2001; Clemons et al., 2000a, 2010; Cohen et al., 2011; Cunha et al., 2010; de Luca et al., 2010; De Luca et al., 2012; Dubourdeau et al., 2006; Espinosa et al., 2014; Garlanda et al., 2002; Gessner et al., 2012; Herbst et al., 2013, 2015; Hohl et al., 2005; Jhingran et al., 2012, 2015; Karki et al., 2015; Kasahara et al., 2016; Leal et al., 2010, 2012, 2013; Mazaki et al., 2006; Mirkov et al., 2012; Moretti et al., 2008, 2012, 2014a; Morton et al., 2012; Park et al., 2009; Phadke et al., 2007; Ramirez-Ortiz et al., 2011; Rivera et al., 2006, 2009, 2011, Romani et al., 2004, 2006; Rubino et al., 2012; Savers et al., 2016; Shepardson et al., 2013; Steele et al., 2005; Stojanovic et al., 2011; Stuehler et al., 2011; Taylor et al., 2014b; Werner et al., 2011; Wharton et al., 2015; Zelante et al., 2007, 2009, 2012, 2015)(Beauvais et al., 2013; Björgvinsdóttir et al., 1997; Bonnett et al., 2006; Cornish et al., 2008; Cruz et al., 2013; D’Angelo et al., 2009; De Luca et al., 2012; de Luca et al., 2014; Dennis et al., 2006; Dinauer et al., 2001; Espinosa et al., 2014; Grimm et al., 2013; Kasahara et al., 2016; Leal et al., 2013; Liu et al., 2013; Morgenstern et al., 1997; Morton et al., 2010; Petersen et al., 2002; Philippe et al., 2003; Pollock et al., 1995; Prüfer et al., 2014; Ren et al., 2010; Röhm et al., 2014; Stein et al., 2013; Vethanayagam et al., 2011; Wharton et al., 2015). |

**Supplementary material 4: Listing of the publications which report usage of main antibiotics to prevent bacterial superinfection in animal models of invasive aspergillosis.** References of articles that report usage of other antibiotics than those below are directly inserted in the text.

| Cyclins | (Alcazar-Fuoli et al., 2015; Armstrong-James et al., 2009; Aufauvre-Brown et al., 1997, 1998; Balloy et al., 2005b; Ben-Ami et al., 2010a, 2013; Ben-Ami and Kontoyiannis, 2012; Bergmann et al., 2009; Bhatti et al., 2011; Björgvinsdóttir et al., 1997; Brown et al., 2000; Chandenier et al., 2009; Cicogna et al., 1997; da Silva Ferreira et al., 2006; Desoubeaux et al., 2014; Desoubeaux and Chandenier, 2012; Dinauer et al., 2001; Ejzykowicz et al., 2009; Hartmann et al., 2011; Herbst et al., 2013, 2015; Hissen et al., 2005; Hood et al., 2010; Huber and Bignell, 2014; Jiang et al., 2014; Kakeya et al., 2008; Kolattukudy et al., 1993; Kothary et al., 1984; Krappmann et al., 2004; Kurtz et al., 1995; Leleu et al., 2013a, 2013b, Lewis et al., 2007, 2011b, 2011a, Li et al., 2007, 2008, 2012; Ma et al., 2008; Martin et al., 1997; McDonagh et al., 2008; Mellado et al., 1996, 2005; Mitsutake et al., 1995; Mitsuyama et al., 2003; Miyazaki et al., 1993; Morgenstern et al., 1997; Morisse et al., 2012, 2013; Murphy et al., 1997; Nawada et al., 1996; Niki et al., 1991; Niwano et al., 1999; O’Hanlon et al., 2011; Olivas et al., 2008; Otsubo et al., 1998, 1999; Panepinto et al., 2003; Qiao et al., 2008; Raghuwanshi et al., 2005; Sasse et al., 2008; Schmitt et al., 1988, 1990, 1991, 1992; Smith et al., 1994; Steinbach et al., 2004; Sugareva et al., 2006; Tang et al., 1993; Ullmann et al., 2007; Wang et al., 2014a, 2014b, 2015, Yang et al., 2009a, 2010; Yasmin et al., 2012; Yonezawa et al., 2000; Yu et al., 1990; Zhang et al., 2005, 2008b, 2013a, 2015; Zhao et al., 2006; Zhou et al., 2007). |
| --- | --- |
| Quinolones | (Aufauvre-Brown et al., 1997; Baistrocchi et al., 2016; Balloy et al., 2005b; Becker et al., 2000, 2002a, 2003, 2006; Brown et al., 2000; Clark et al., 2016; Dams et al., 1999; Gravelat et al., 2013; Hachem et al., 2006; Herbst et al., 2013, 2015; Huber and Bignell, 2014; Ito et al., 2006; Ito and Lyons, 2002; Jambunathan et al., 2013; Krappmann et al., 2004; Leenders et al., 1996; Lin et al., 2014; McCulloch et al., 2009, 2012; McDonagh et al., 2008; Petrik et al., 2010, 2012, 2014; Philippe et al., 2003; Rebong et al., 2011; Ren et al., 2010; Ruijgrok et al., 2001, 2005, 2006; Scotter and Chambers, 2005; Van Etten et al., 2000; van Vianen et al., 2006; Verwer et al., 2013; Wiederhold et al., 2013, 2015; Xu et al., 2015; Zhang et al., 2014; Zhao et al., 2010; Zhao and Perlin, 2013; Zhong et al., 2009) |
| Cephalosporins | (Björgvinsdóttir et al., 1997; Chiang et al., 2006, 2008; Cramer et al., 2006; Dinauer et al., 2001; Ejzykowicz et al., 2009, 2010; Gravelat et al., 2008; Hooper et al., 2012, 20; Kirkpatrick et al., 2000, 2002a, 2002b, 2006, 2012; Lengerova et al., 2012; Lepak et al., 2013b, 2013a, 2013c, Lin et al., 2010, 2012; Morgenstern et al., 1997; Niki et al., 1991; Pollock et al., 1995; Rieg et al., 2006; Sheppard et al., 2004, 2006a; Singh et al., 2014; Stein et al., 2013; Steinbach et al., 2004; Vallor et al., 2008; White et al., 2016; Wiederhold et al., 2008) |
| Aminosides | (Amich et al., 2013, 2016, Becker et al., 2000, 2002b, 2002a, 2003, 2006; BitMansour et al., 2002; BitMansour and Brown, 2002; Cramer et al., 2008; Dams et al., 1999; Grahl et al., 2011; Hashimoto et al., 1998; Kirkpatrick et al., 2012; Kolattukudy et al., 1993; Leenders et al., 1996; Martinez et al., 2013; Pinchai et al., 2009; Rivera et al., 2009; Ruijgrok et al., 2001, 2005, 2006; Scotter and Chambers, 2005; Steinbach et al., 2006; Van Etten et al., 2000) |

**Supplementary material 5: Listing of the publications which report usage of main *Aspergillus fumigatus* strains for infectious challenge.** Articles that describe alternative fungal strains to those below are directly referenced in the text.

| AF293 | (Abad-Diaz-De-Cerio et al., 2013; Al-Bader et al., 2010; Alvarez et al., 2007; Amarsaikhan et al., 2014; Appel et al., 2010; Arnusch et al., 2012; Baistrocchi et al., 2016; Bedke et al., 2014; Bellocchio et al., 2005; Ben-Ami et al., 2009, 2010b, 2010a, 2013; Ben-Ami and Kontoyiannis, 2012; Bok et al., 2005, 2006; Bozza et al., 2014; Bretz et al., 2008; Caffrey et al., 2015; Caretti et al., 2016; Carvalho et al., 2012; Chaudhary et al., 2012; Chiang et al., 2006, 2008; Clark et al., 2016; Cramer et al., 2006, 2008; Dagenais et al., 2008; De Luca et al., 2012; de Luca et al., 2014; Dinamarco et al., 2012b; Durrant et al., 2011; Ejzykowicz et al., 2009, 2010; Espinosa et al., 2014; Evans et al., 2010b, 2010a; Gaziano et al., 2004; Gravelat et al., 2008, 2013; Gresnigt et al., 2014; Hachem et al., 2006; Herbst et al., 2013; Hohl et al., 2005, 2009; Hooper et al., 2012; Iannitti et al., 2013, 2016, Ibrahim et al., 2010, 2011; Jambunathan et al., 2013; Jhingran et al., 2015; Juvvadi et al., 2013; Karki et al., 2015; Kasahara et al., 2016; Kerr et al., 2016; Khalaj et al., 2012; Kirkpatrick et al., 2012; Kumaresan et al., 2014; Leal et al., 2012; Lee et al., 2009; Leleu et al., 2013a, 2013b; Lengerova et al., 2012; Lepak et al., 2013b, 2013a, 2013c; Levdansky et al., 2010; Lewis et al., 2002, 2007, 2008b, 2008a, 2011a, 2011b, 2014; Li et al., 2011; Lin et al., 2010, 2012; Lionakis et al., 2005; Liu et al., 2010; Lo Giudice et al., 2010; Ma et al., 2008; Marples et al., 2011; Martinez et al., 2015; McDonagh et al., 2008; Mircescu et al., 2009; Monroy and Sheppard, 2005; Moretti et al., 2014b, 2014a; Morton et al., 2012; O’Dea et al., 2014; Olivas et al., 2008; Paisley et al., 2005; Pinchai et al., 2009; Pongpom et al., 2015; Qiao et al., 2008; Quezada et al., 2008; Ramirez-Ortiz et al., 2011; Rammaert et al., 2015; Rebong et al., 2011; Rieg et al., 2006; Rivera et al., 2005, 2006, 2009, 2011; Rizzetto et al., 2013; Röhm et al., 2014; Romano et al., 2006; Schlitzer et al., 2013; Sekonyela et al., 2013; Shadkchan et al., 2004; Sharon et al., 2009; Sheppard et al., 2004; Steinbach et al., 2004; Templeton et al., 2011; Tolman et al., 2009; Tsitsigiannis et al., 2005; Vallor et al., 2008; Van Epps et al., 2003; Warn et al., 2006, 2010; White et al., 2016; Wiederhold et al., 2004, 2008, 2009, 2013, 2015; Wong Sak Hoi et al., 2011; Yang et al., 2009b; Zaas et al., 2008; Zelante et al., 2012, 2015, Zhang et al., 2013b, 2015) |
| --- | --- |
| Dal/CEA10 | (Aimanianda et al., 2009; Alsaadi et al., 2012; Appel et al., 2010; Balloy et al., 2005b, 2005a; Beauvais et al., 2005; Bertuzzi et al., 2014; Caffrey et al., 2015; Chai et al., 2011; Chung et al., 2014; D’Enfert et al., 1996; Dubourdeau et al., 2006; Fleck and Brock, 2010; Fontaine et al., 2011; Gehrke et al., 2010; Grahl et al., 2011, 2012, Herbst et al., 2013, 2015; Hu et al., 2007; Ibrahim-Granet et al., 2008; Italia et al., 2011; Jambunathan et al., 2013; Jaton-Ogay et al., 1994; Kasahara et al., 2016; Lamarre et al., 2007; Leal et al., 2012; Loussert et al., 2010; Macheleidt et al., 2015; Monod et al., 1993; Moutaouakil et al., 1993; Mouyna et al., 2010; Oliver et al., 2012; Philippe et al., 2003; Puttikamonkul et al., 2010; Rajendran et al., 2011; Rizzetto et al., 2013; Rubino et al., 2012; Sasse et al., 2008; Schmalhorst et al., 2008; Schrettl et al., 2004; Shepardson et al., 2013, 2014; Shirkhani et al., 2015; Slesiona et al., 2012; Svirshchevskaya et al., 2009; Thau et al., 1994; Vallon-Eberhard et al., 2008; Warn et al., 2006, 2010; Wiederhold et al., 2008; Willger et al., 2008, 2012; Wong Sak Hoi et al., 2011; Zhao et al., 2010; Zhao and Perlin, 2013) |
| Ku80 | (Ben-Ami et al., 2010a; Ben-Ami and Kontoyiannis, 2012; Blatzer et al., 2011; Bom et al., 2015; Chung et al., 2014; da Silva Ferreira et al., 2006, 2007, de Castro et al., 2014a, 2014b; de Gouvêa et al., 2008; D’Enfert et al., 1996; Dinamarco et al., 2012b, 2012a; Gehrke et al., 2010; Grahl et al., 2011; Hu et al., 2007; Kupfahl et al., 2006; Leal et al., 2012; Liebmann et al., 2004b; Mota Júnior et al., 2008; Mouyna et al., 2010; Muszkieta et al., 2014a, 2014b; Puttikamonkul et al., 2010; Schrettl et al., 2004; Valiante et al., 2008; Wartenberg et al., 2011; Willger et al., 2008, 2012; Winkelströter et al., 2015; Wong Sak Hoi et al., 2011) |
| H11-20 | (Allendoerfer et al., 1995; Cicogna et al., 1997; Hashiguchi et al., 1994; Kurtz et al., 1995; Murphy et al., 1997; Niki et al., 1991; Schmitt et al., 1988, 1990, 1991, 1992, Takemoto et al., 2004, 2006, 2009; Ullmann et al., 2007; Yu et al., 1990) |
| Unreferenced | (Arber et al., 2005; Arendrup et al., 2010; Arroyo et al., 1977; Aufauvre-Brown et al., 1998; Bartroli et al., 1998a; Becker et al., 2000, 2002a, 2002b, 2003, 2006, Bellocchio et al., 2004b, 2004a; Benjamim et al., 2005; BitMansour et al., 2002, 2005; BitMansour and Brown, 2002; Bonifazi et al., 2010; Bozza et al., 2002a, 2002b, 2003, 2008, 2009; Carvalho et al., 2012; Casadio et al., 1973; Cenci et al., 1997, 1998, 1999, 2000, 2001, 2002; Centeno-Lima et al., 2002; Chandenier et al., 2009; Chaturvedi et al., 2005; Clark et al., 1991; Cunha et al., 2010; Dabur et al., 2005; Dams et al., 1999; D’Angelo et al., 2009; de Luca et al., 2010; Del Sero et al., 1999; Dennis et al., 2006; Desai and Naik, 2009; Desoubeaux and Chandenier, 2012; El-Muzghi et al., 2013; Fahmy et al., 2014; Fei et al., 2011; Fortwendel et al., 2005; García et al., 2006; Garlanda et al., 2002; Graybill et al., 1983; Graybill and Kaster, 1984; Habicht et al., 2002; Ito and Lyons, 2002; Khan et al., 2006; Khosravi et al., 2012, 201; Kolattukudy et al., 1993; Kothary et al., 1984; Land et al., 1989; Lee and Kolattukudy, 1995; Leenders et al., 1996; Li et al., 2014; Lin et al., 2014; Lupetti et al., 2002; Markaryan et al., 1994; Martín et al., 2003; Mazaki et al., 2006; Mazzolla et al., 1991; Mirkov et al., 2010, 2011, 2012, 2013, 2014, 2015; Moalli et al., 2010; Mondon et al., 1996; Monga, 1983; Montagnoli et al., 2003, 2006; Moretti et al., 2008; Morisse et al., 2012, 2013; Morton et al., 2010; Najvar et al., 1995; Nobre, 1977; Odds et al., 1998; Okawa et al., 2002; Plempel, 1984; Pollock et al., 1995; Pope and Davies, 1979; Risovic et al., 2007; Rizzetto et al., 2013; Romani et al., 2004, 2008, Ruijgrok et al., 2001, 2005, 2006, Sandhu et al., 1970, 1976; Saxena et al., 1998; Schaffner et al., 1982; Schaffner and Böhler, 1993; Schaffner and Frick, 1985; Scotter and Chambers, 2005; Seyedmousavi et al., 2014; Shao et al., 2005a, 2005b; Shibata et al., 2014; Singh et al., 2014; Smith, 1972, 1977; Sorci et al., 2011; Spreghini et al., 2009; Stojanovic et al., 2011; Sutton et al., 1996; Tanio et al., 1990; Tkalcevic et al., 2000; Turner et al., 1975a, 1975b, 1976, van de Sande et al., 2008, 2009; Van Etten et al., 2000; van Vianen et al., 2006; Vecchiarelli et al., 1988; Verwer et al., 2013; Vethanayagam et al., 2011; Waldorf et al., 1984; Waldorf and Diamond, 1985; Wallace et al., 1997; Wang et al., 2014a; Wasan et al., 2007, 2009; Williams et al., 1981; Xu et al., 2009; Zelante et al., 2007, 2009, Zhang et al., 2008a, 2013a, 2014; Zimmerli et al., 2007) |

**Supplementary material 6: Listing of the publications which report usage of main routes of infection as alternatives to intranasal deposition and intravenous challenge.** References that deal with other routes of infection than those listed below are directly inserted in the text.

| Passive inhalation | (Ahmad et al., 2014; Al-Bader et al., 2010; Alsaadi et al., 2012; Alvarez et al., 2007; Baisakh et al., 1975; Bennett et al., 2006; Bretz et al., 2008; Brieland et al., 2001; Buskirk et al., 2014; Cacciapuoti et al., 2000; Chaudhary et al., 2012; Chiang et al., 2006, 2008, Cramer et al., 2006, 2008; Dufresne et al., 2012; Ejzykowicz et al., 2010; Evans et al., 2010a, 2010b; Ford and Friedman, 1967; Grahl et al., 2011; Gravelat et al., 2008, 2010, 2013; Hooper et al., 2012; Howard et al., 2011; Ibrahim et al., 2010, 2011; Italia et al., 2011; Jambunathan et al., 2013; Kakeya et al., 2008; Kirkpatrick et al., 2012; Kothary et al., 1984; Lamoth et al., 2014b, 2014a; Le Conte et al., 1992; Leleu et al., 2013a, 2013b; Lengerova et al., 2012; Li et al., 2011; Lin et al., 2012; Liu et al., 2010; McCulloch et al., 2009, 2012; Oliver et al., 2012; Patera et al., 2004; Pinchai et al., 2009, 2010; Pongpom et al., 2015; Rieg et al., 2006; Schaffner et al., 1982; Shepardson et al., 2013; Sheppard et al., 2004, 2006b, 2006a; Steinbach et al., 2004; Stephens-Romero et al., 2005; Tolman et al., 2009; Vallor et al., 2008; White et al., 2016; Wiederhold et al., 2008, 2009, 2013, 2015; Zaas et al., 2008; Zhao et al., 2010; Zhao and Perlin, 2013) |
| --- | --- |
| Bronchio-tracheal instillation | (Baistrocchi et al., 2016; Becker et al., 2000, 2002b, 2002a, 2003, 2006; Chandenier et al., 2009; Cicogna et al., 1997; Dams et al., 1999; Desoubeaux et al., 2014; Desoubeaux and Chandenier, 2012; El-Muzghi et al., 2013; Habicht et al., 2002; Hanazawa et al., 2000; Hashiguchi et al., 1994; Hashimoto et al., 1998; Khan et al., 2008; Kurtz et al., 1995; Land et al., 1989; Leenders et al., 1996; Lin et al., 2014; Lo Giudice et al., 2010, 2012; Marra et al., 2014; Martín et al., 2003; Mirkov et al., 2014, 2015; Mitsutake et al., 1995; Miyazaki et al., 1993; Murphy et al., 1997; Najvar et al., 1995; Niki et al., 1991; Niwano et al., 1999; Otsubo et al., 1999; Paris et al., 2003; Petrik et al., 2010, 2012, 2014, Ruijgrok et al., 2001, 2005, 2006, Schmitt et al., 1988, 1990, 1991; Shibuya et al., 1999; Turner et al., 1976; Ullmann et al., 2007; van de Sande et al., 2008, 2009; Van Etten et al., 2000; van Vianen et al., 2006; Verwer et al., 2013; Yu et al., 1990; Zhang et al., 2014) |
| Intra-ocular administration | (Carrion et al., 2013; Clark et al., 2016; Gresnigt et al., 2014; Guo et al., 2012; Huang et al., 2014; Jiang et al., 2015; Jie Zhao et al., 2009; Leal et al., 2010, 2012, 2013; Li et al., 2015a; Rebong et al., 2011; Ren et al., 2010; Taylor et al., 2014a, 2014b; Xu et al., 2015; Zhao et al., 2015; Zhong et al., 2009) |
| Intracerebral injection | (Chiller et al., 2002, 2003, Clemons et al., 2005, 2006, 2012, Imai et al., 2004, 2005; Mazzolla et al., 1991; Morton et al., 2011; Singh et al., 2005; Zimmerli et al., 2007) |

**Supplementary material 7: Listing of the publications which report usage of main biomarkers and surrogate endpoints for validation of correct infection and assessment of fungal load.** References that focus on other means for diagnosis and fungal burden assessment than those below are directly inserted in the text.

| Phenotypic methods  (histopathology – mycological culture) | (Abad-Diaz-De-Cerio et al., 2013; Ahmad et al., 2014; Alcazar-Fuoli et al., 2015; Allendoerfer et al., 1995; Alvarez et al., 2007; Appel et al., 2010; Arber et al., 2005; Arrese et al., 1994; Arroyo et al., 1977; Aufauvre-Brown et al., 1998; Baisakh et al., 1975; Barchiesi et al., 2016; Beauvais et al., 2005, 2013, Becker et al., 2002a, 2002b, 2003; Bedke et al., 2014; Bellocchio et al., 2004a; Ben-Ami et al., 2010a; Ben-Ami and Kontoyiannis, 2012; Bhabhra et al., 2004; BitMansour et al., 2002, 2005; Bonifazi et al., 2010; Bonnett et al., 2006; Bozza et al., 2009, 2014; Bretz et al., 2008; Brown et al., 2000; Carrion et al., 2013; Carvalho et al., 2012; Chiller et al., 2002, 2003; Clark et al., 2016; Clemons et al., 2000a, 2011, 2012; Cohen et al., 2011; Corbel and Eades, 1977; Cunha et al., 2010; Cutsem et al., 1993; Dams et al., 1999; de Luca et al., 2010; De Luca et al., 2012; de Luca et al., 2014; de Repentigny et al., 1993; Defaveri et al., 1990; Del Sero et al., 1999; Denning and Stevens, 1991; Desoubeaux et al., 2014; Duong et al., 1998; El-Muzghi et al., 2013; Espinosa et al., 2014; Fahmy et al., 2014; Fei et al., 2011; Feng et al., 2011; Fontaine et al., 2011; Fortwendel et al., 2005; Gaziano et al., 2004; Grahl et al., 2011, 2012, Graybill et al., 1983, 1998a; Graybill and Kaster, 1984; Gresnigt et al., 2014; Hachem et al., 2006; Hanson et al., 1995; Hashimoto et al., 1998; Herbst et al., 2015; Hood et al., 2010; Hooper et al., 2012; Hsu et al., 2013; Iannitti et al., 2016; Ibrahim-Granet et al., 2010; Jaton-Ogay et al., 1994; Jhingran et al., 2012; Kasahara et al., 2016; Kaur et al., 2007; Khosravi et al., 2012, 201; Kolattukudy et al., 1993; Kothary et al., 1984; Krappmann et al., 2004; Kretschmar et al., 2001; Kwon-Chung, 1975; Land et al., 1989; Lass-Flörl et al., 2003; Le Conte et al., 1992; Leal et al., 2010, 2012; Leenders et al., 1996; Leleu et al., 2013a, 2013b; Lewis et al., 2002; Li et al., 2012, 2014, Lin et al., 2010, 2012, 2014; Liu et al., 2013; Madan et al., 2001, 2010; Marples et al., 2011; Martinez et al., 2015; Mazaki et al., 2006; Melchers et al., 1994; Mirkov et al., 2010, 2011, 2012, 2013, 2014, 2015; Mitsutake et al., 1995; Mitsuyama et al., 2003; Moalli et al., 2010; Mondon et al., 1996; Monod et al., 1993; Moretti et al., 2012, 2014a; Morgenstern et al., 1997; Morisse et al., 2012, 2013; Morrison et al., 2003; Morton et al., 2011, 2012; Muszkieta et al., 2014b; Nagai et al., 1995; Nagasaki et al., 2009; Nawada et al., 1996; Niki et al., 1991; Okawa et al., 2002; Olson et al., 2006, 2015; Otsubo et al., 1998; Paris et al., 1993; Petrik et al., 2010; Poelmans et al., 2016; Pollock et al., 1995; Prüfer et al., 2014; Qiao et al., 2008; Reichard et al., 1997; Rieg et al., 2006; Rivera et al., 2011; Rizzetto et al., 2013; Rüchel et al., 2000; Ruijgrok et al., 2001, 2005, 2006, Sandhu et al., 1970, 1976; Schaffner and Frick, 1985; Scotter and Chambers, 2005; Shao et al., 2005b; Sheppard et al., 2004; Shirkhani et al., 2015; Singh et al., 2014; Sionov et al., 2005, 2006, Sionov and Segal, 2003, 2004; Smith et al., 1993; Spreghini et al., 2009; Steinbach et al., 2006; Stephens-Romero et al., 2005; Stuehler et al., 2011; Sun et al., 2012; Takazono et al., 2009; Tang et al., 1993; Tansho et al., 2006; Tkalcevic et al., 2000; Tolman et al., 2009; Ullmann et al., 2007; Van Cutsem et al., 1990; Van Etten et al., 2000; Verwer et al., 2013; Vethanayagam et al., 2011; Wallace et al., 1997; Wang et al., 2014b, 2014a, Wiederhold et al., 2013, 2015; Williams et al., 1981; Wong et al., 1989; Xu et al., 2009; Yamakami et al., 1996; Yang et al., 2010; Yonezawa et al., 2000; Yu et al., 1990; Zelante et al., 2012; Zhang et al., 2013a, 2013b, 2014, 2015; Zhao et al., 2015; Zimmerli et al., 2007) |
| --- | --- |
| Galactomannan antigen detection | (Ahmad et al., 2014; Al-Bader et al., 2010; Arendrup et al., 2008, 2008; Arrese et al., 1994; Balloy et al., 2005a; Beauvais et al., 2005; Becker et al., 2000, 2002a, 2002b, 2003; Ben-Ami et al., 2010a; Ben-Ami and Kontoyiannis, 2012; Chai et al., 2011; Chandenier et al., 2009; Chiang et al., 2008; Cutsem et al., 1993; Dennis et al., 2006; Desoubeaux et al., 2014; Desoubeaux and Chandenier, 2012; Donat et al., 2012; Dufresne et al., 2012; Ejzykowicz et al., 2009, 2010; Evans et al., 2010a; Gravelat et al., 2010, 2013; Hashimoto et al., 1998; Howard et al., 2011; Jambunathan et al., 2013; Jensen et al., 1996b; Khan et al., 2008; Kirkpatrick et al., 2012; Kretschmar et al., 2001; Leleu et al., 2013a, 2013b; Lengerova et al., 2012; Lin et al., 2014; Liu et al., 2010; Lo Giudice et al., 2010, 2012, 201; Marra et al., 2014; Maubon et al., 2006, 2006; Mitsutake et al., 1995; Morton et al., 2011; Nagasaki et al., 2009; Olson et al., 2010; Poelmans et al., 2016; Risovic et al., 2007; Salas et al., 2013; Scotter and Chambers, 2005; Sheppard et al., 2006b; Shibuya et al., 1999; Sionov et al., 2005, 2006; Vallor et al., 2008; Van Cutsem et al., 1990; van de Sande et al., 2008, 2009; van Vianen et al., 2006; Verwer et al., 2013; Vethanayagam et al., 2011; Wasan et al., 2007; White et al., 2016; Wiederhold et al., 2009, 2013, 2015; Yamakami et al., 1996; Zhang et al., 2014; Zimmerli et al., 2007) |
| β-D-glucans measurement | (Ahmad et al., 2014; Gravelat et al., 2013; Hashimoto et al., 1998; Hayashi et al., 2002; Jambunathan et al., 2013; Khan et al., 2008; Lengerova et al., 2012; Lewis et al., 2007, 2011b; McCulloch et al., 2012; Mitsutake et al., 1995; White et al., 2016; Wiederhold et al., 2008, 2009, 2013, 2015) |
| Polymerase chain reaction | (Abad-Diaz-De-Cerio et al., 2013; Ahmad et al., 2014; Alcazar-Fuoli et al., 2015; Alsaadi et al., 2012; Alvarez et al., 2007; Amarsaikhan et al., 2014; Arendrup et al., 2010; Beauvais et al., 2005; Becker et al., 2000; Ben-Ami et al., 2010b, 2010a, 2013; Ben-Ami and Kontoyiannis, 2012; Bergmann et al., 2009; Bhatia et al., 2011; Bhatti et al., 2011; Bom et al., 2015; Bowman et al., 2001; Clemons et al., 2002; de Castro et al., 2014a, 2014b; Dennis et al., 2006; Dinamarco et al., 2012b, 2012a; Evans et al., 2010a; Fortwendel et al., 2005; García et al., 2002; Gessner et al., 2012, 2013, Grahl et al., 2011, 2012; Hartmann et al., 2011; Hashimoto et al., 1998; Herbst et al., 2013; Hooper et al., 2012; Hsu et al., 2013; Hummel et al., 2004; Ibrahim et al., 2010, 2011; Ibrahim-Granet et al., 2010; Jhingran et al., 2015; Kerr et al., 2016; Khan et al., 2008; Kirkpatrick et al., 2012; Kumaresan et al., 2014; Lass-Flörl et al., 2003; Leal et al., 2013; Lengerova et al., 2012; Lepak et al., 2013b, 2013a, 2013c, Lewis et al., 2005, 2007, 2008a, 2008b, 2011a, 2011b, 2014, Li et al., 2011, 2014; Lin et al., 2014; Loeffler et al., 2002; MacCallum et al., 2005; Maubon et al., 2006; McCulloch et al., 2009, 2012; Melchers et al., 1994; Mondon et al., 1996; Moretti et al., 2012; Morton et al., 2010, 2011, 2012; Mouyna et al., 2010; Muszkieta et al., 2014b; O’Dea et al., 2014; O’Hanlon et al., 2011; Quezada et al., 2008; Rubino et al., 2012; Schmalhorst et al., 2008; Scotter and Chambers, 2005; Seyedmousavi et al., 2015a; Shepardson et al., 2014; Sheppard et al., 2006b; Shirkhani et al., 2015; Singh et al., 2005; Spreadbury et al., 1993; Spreghini et al., 2009; Steinbach et al., 2004; Sun et al., 2012; Takemoto et al., 2009; Tanaka et al., 2015; Tolman et al., 2009; Vallor et al., 2008; van de Sande et al., 2008; van Vianen et al., 2006; Wang et al., 2014a; Werner et al., 2009; Wharton et al., 2015; White et al., 2016; Wiederhold et al., 2004, 2006, 2008, 2015; Winkelströter et al., 2015; Yamakami et al., 1996; Yang et al., 2010; Zaas et al., 2008; Zelante et al., 2015; Zhang et al., 2015; Zhao et al., 2010; Zhao and Perlin, 2013) |
| Chitin assay | (Balloy et al., 2005b, 2005a, Becker et al., 2002b, 2003; Bellocchio et al., 2005; Bozza et al., 2002a; Carpenter and Hogaboam, 2005; Cenci et al., 1997, 1998, 1999, 2000, 2001, 2002; D’Angelo et al., 2009; de Repentigny et al., 1993; Del Sero et al., 1999; Gavaldà et al., 2005; Hartigan et al., 2009; Hayashi et al., 2002; Hood et al., 2010; Lehmann and White, 1975, 1976; Lewis et al., 2002; Martín et al., 2003; Mehrad et al., 1999a, 1999b, 2000, 2002; Morrison et al., 2003; Overdijk et al., 1996; Park et al., 2009, 2010; Phadke et al., 2007; Romani et al., 2004, 2006; Singh et al., 2009; Stephens-Romero et al., 2005; Zelante et al., 2007, 2009) |

1. **References**

Abad-Diaz-De-Cerio, A., Fernandez-Molina, J. V., Ramirez-Garcia, A., Sendino, J., Hernando, F. L., Pemán, J., et al. (2013). The aspHS gene as a new target for detecting Aspergillus fumigatus during infections by quantitative real-time PCR. *Med. Mycol.* 51, 545–554. doi:10.3109/13693786.2012.756989.

Abruzzo, G. K., Flattery, A. M., Gill, C. J., Kong, L., Smith, J. G., Krupa, D., et al. (1995). Evaluation of water-soluble pneumocandin analogs L-733560, L-705589, and L-731373 with mouse models of disseminated aspergillosis, candidiasis, and cryptococcosis. *Antimicrob. Agents Chemother.* 39, 1077–1081.

Abruzzo, G. K., Flattery, A. M., Gill, C. J., Kong, L., Smith, J. G., Pikounis, V. B., et al. (1997). Evaluation of the echinocandin antifungal MK-0991 (L-743,872): efficacies in mouse models of disseminated aspergillosis, candidiasis, and cryptococcosis. *Antimicrob. Agents Chemother.* 41, 2333–2338.

Abruzzo, G. K., Gill, C. J., Flattery, A. M., Kong, L., Leighton, C., Smith, J. G., et al. (2000). Efficacy of the echinocandin caspofungin against disseminated aspergillosis and candidiasis in cyclophosphamide-induced immunosuppressed mice. *Antimicrob. Agents Chemother.* 44, 2310–2318.

Adamson, T. W., Diaz-Arevalo, D., Gonzalez, T. M., Liu, X., and Kalkum, M. (2013). Hypothermic endpoint for an intranasal invasive pulmonary aspergillosis mouse model. *Comp. Med.* 63, 477–481.

Ahmad, I., Perkins, W. R., Lupan, D. M., Selsted, M. E., and Janoff, A. S. (1995). Liposomal entrapment of the neutrophil-derived peptide indolicidin endows it with in vivo antifungal activity. *Biochim. Biophys. Acta* 1237, 109–114.

Ahmad, I., Sarkar, A. K., and Bachhawat, B. K. (1989a). Design of liposomes to improve delivery of amphotericin-B in the treatment of aspergillosis. *Mol. Cell. Biochem.* 91, 85–90.

Ahmad, I., Sarkar, A. K., and Bachhawat, B. K. (1989b). Liposomal amphotericin-B in the control of experimental aspergillosis in mice: Part I--Relative therapeutic efficacy of free and liposomal amphotericin-B. *Indian J. Biochem. Biophys.* 26, 351–356.

Ahmad, I., Sarkar, A. K., and Bachhawat, B. K. (1990a). Effect of cholesterol in various liposomal compositions on the in vivo toxicity, therapeutic efficacy, and tissue distribution of amphotericin B. *Biotechnol. Appl. Biochem.* 12, 550–556.

Ahmad, I., Sarkar, A. K., and Bachhawat, B. K. (1990b). Liposomal amphotericin-B as a therapeutic measure to control experimental aspergillosis in BALB/c mice. *Indian J. Biochem. Biophys.* 27, 370–374.

Ahmad, S., Al-Shaikh, A. A., and Khan, Z. (2014). Development of a novel inhalational model of invasive pulmonary aspergillosis in rats and comparative evaluation of three biomarkers for its diagnosis. *PloS One* 9, e100524. doi:10.1371/journal.pone.0100524.

Aimanianda, V., Bayry, J., Bozza, S., Kniemeyer, O., Perruccio, K., Elluru, S. R., et al. (2009). Surface hydrophobin prevents immune recognition of airborne fungal spores. *Nature* 460, 1117–1121. doi:10.1038/nature08264.

Al-Bader, N., Vanier, G., Liu, H., Gravelat, F. N., Urb, M., Hoareau, C. M.-Q., et al. (2010). Role of trehalose biosynthesis in Aspergillus fumigatus development, stress response, and virulence. *Infect. Immun.* 78, 3007–3018. doi:10.1128/IAI.00813-09.

Alcazar-Fuoli, L., Buitrago, M., Gomez-Lopez, A., and Mellado, E. (2015). An alternative host model of a mixed fungal infection by azole susceptible and resistant Aspergillus spp strains. *Virulence* 6, 376–384. doi:10.1080/21505594.2015.1025192.

Allen, S. D., Sorensen, K. N., Nejdl, M. J., Durrant, C., and Proffit, R. T. (1994). Prophylactic efficacy of aerosolized liposomal (AmBisome) and non-liposomal (Fungizone) amphotericin B in murine pulmonary aspergillosis. *J. Antimicrob. Chemother.* 34, 1001–1013.

Allendoerfer, R., Loebenberg, D., Rinaldi, M. G., and Graybill, J. R. (1995). Evaluation of SCH51048 in an experimental model of pulmonary aspergillosis. *Antimicrob. Agents Chemother.* 39, 1345–1348.

Alsaadi, M., Italia, J. L., Mullen, A. B., Ravi Kumar, M. N. V., Candlish, A. A., Williams, R. a. M., et al. (2012). The efficacy of aerosol treatment with non-ionic surfactant vesicles containing amphotericin B in rodent models of leishmaniasis and pulmonary aspergillosis infection. *J. Control. Release Off. J. Control. Release Soc.* 160, 685–691. doi:10.1016/j.jconrel.2012.04.004.

Alvarez, C. A., Wiederhold, N. P., McConville, J. T., Peters, J. I., Najvar, L. K., Graybill, J. R., et al. (2007). Aerosolized nanostructured itraconazole as prophylaxis against invasive pulmonary aspergillosis. *J. Infect.* 55, 68–74. doi:10.1016/j.jinf.2007.01.014.

Amarsaikhan, N., O’Dea, E. M., Tsoggerel, A., Owegi, H., Gillenwater, J., and Templeton, S. P. (2014). Isolate-dependent growth, virulence, and cell wall composition in the human pathogen Aspergillus fumigatus. *PloS One* 9, e100430. doi:10.1371/journal.pone.0100430.

Amich, J., Dümig, M., O’Keeffe, G., Binder, J., Doyle, S., Beilhack, A., et al. (2016). Exploration of Sulfur Assimilation of Aspergillus fumigatus Reveals Biosynthesis of Sulfur-Containing Amino Acids as a Virulence Determinant. *Infect. Immun.* 84, 917–929. doi:10.1128/IAI.01124-15.

Amich, J., Schafferer, L., Haas, H., and Krappmann, S. (2013). Regulation of sulphur assimilation is essential for virulence and affects iron homeostasis of the human-pathogenic mould Aspergillus fumigatus. *PLoS Pathog.* 9, e1003573. doi:10.1371/journal.ppat.1003573.

Appel, E., Vallon-Eberhard, A., Rabinkov, A., Brenner, O., Shin, I., Sasson, K., et al. (2010). Therapy of murine pulmonary aspergillosis with antibody-alliinase conjugates and alliin. *Antimicrob. Agents Chemother.* 54, 898–906. doi:10.1128/AAC.01267-09.

Aratani, Y., Kura, F., Watanabe, H., Akagawa, H., Takano, Y., Suzuki, K., et al. (2000). Differential host susceptibility to pulmonary infections with bacteria and fungi in mice deficient in myeloperoxidase. *J. Infect. Dis.* 182, 1276–1279. doi:10.1086/315843.

Arber, C., Bitmansour, A., Shashidhar, S., Wang, S., Tseng, B., and Brown, J. M. Y. (2005). Protection against lethal Aspergillus fumigatus infection in mice by allogeneic myeloid progenitors is not major histocompatibility complex restricted. *J. Infect. Dis.* 192, 1666–1671. doi:10.1086/491743.

Arendrup, M. C., Mavridou, E., Mortensen, K. L., Snelders, E., Frimodt-Møller, N., Khan, H., et al. (2010). Development of azole resistance in Aspergillus fumigatus during azole therapy associated with change in virulence. *PloS One* 5, e10080. doi:10.1371/journal.pone.0010080.

Arendrup, M. C., Perkhofer, S., Howard, S. J., Garcia-Effron, G., Vishukumar, A., Perlin, D., et al. (2008). Establishing in vitro-in vivo correlations for Aspergillus fumigatus: the challenge of azoles versus echinocandins. *Antimicrob. Agents Chemother.* 52, 3504–3511. doi:10.1128/AAC.00190-08.

Armstrong-James, D. P. H., Turnbull, S. A., Teo, I., Stark, J., Rogers, N. J., Rogers, T. R. F., et al. (2009). Impaired interferon-gamma responses, increased interleukin-17 expression, and a tumor necrosis factor-alpha transcriptional program in invasive aspergillosis. *J. Infect. Dis.* 200, 1341–1351. doi:10.1086/605931.

Arnusch, C. J., Albada, H. B., van Vaardegem, M., Liskamp, R. M. J., Sahl, H.-G., Shadkchan, Y., et al. (2012). Trivalent ultrashort lipopeptides are potent pH dependent antifungal agents. *J. Med. Chem.* 55, 1296–1302. doi:10.1021/jm2014474.

Arrese, J. E., Delvenne, P., Van Cutsem, J., Piérard-Franchimont, C., and Piérard, G. E. (1994). Experimental aspergillosis in guinea pigs: influence of itraconazole on fungaemia and invasive fungal growth. *Mycoses* 37, 117–122.

Arroyo, J., Medoff, G., and Kobayashi, G. S. (1977). Therapy of murine aspergillosis with amphotericin B in combination with rifampin of 5-fluorocytosine. *Antimicrob. Agents Chemother.* 11, 21–25.

Aufauvre-Brown, A., Brown, J. S., and Holden, D. W. (1998). Comparison of virulence between clinical and environmental isolates of Aspergillus fumigatus. *Eur. J. Clin. Microbiol. Infect. Dis. Off. Publ. Eur. Soc. Clin. Microbiol.* 17, 778–780.

Aufauvre-Brown, A., Mellado, E., Gow, N. A., and Holden, D. W. (1997). Aspergillus fumigatus chsE: a gene related to CHS3 of Saccharomyces cerevisiae and important for hyphal growth and conidiophore development but not pathogenicity. *Fungal Genet. Biol. FG B* 21, 141–152.

Baisakh, K. M., Agarwal, D. S., Iyengar, B., and Bhatia, V. N. (1975). Experimental aspergillosis and phycomycosis in mice. *Indian J. Med. Res.* 63, 1716–1731.

Baistrocchi, S. R., Lee, M. J., Lehoux, M., Ralph, B., Snarr, B. D., Robitaille, R., et al. (2016). Posaconazole-loaded leukocytes as a novel treatment strategy targeting invasive pulmonary aspergillosis. *J. Infect. Dis.* doi:10.1093/infdis/jiw513.

Balloy, V., Huerre, M., Latgé, J.-P., and Chignard, M. (2005a). Differences in patterns of infection and inflammation for corticosteroid treatment and chemotherapy in experimental invasive pulmonary aspergillosis. *Infect. Immun.* 73, 494–503. doi:10.1128/IAI.73.1.494-503.2005.

Balloy, V., Si-Tahar, M., Takeuchi, O., Philippe, B., Nahori, M.-A., Tanguy, M., et al. (2005b). Involvement of toll-like receptor 2 in experimental invasive pulmonary aspergillosis. *Infect. Immun.* 73, 5420–5425. doi:10.1128/IAI.73.9.5420-5425.2005.

Barchiesi, F., Santinelli, A., Biscotti, T., Greganti, G., Giannini, D., and Manso, E. (2016). Delay of antifungal therapy influences the outcome of invasive aspergillosis in experimental models of infection. *J. Antimicrob. Chemother.* 71, 2230–2233. doi:10.1093/jac/dkw111.

Bartroli, J., Turmo, E., Algueró, M., Boncompte, E., Vericat, M. L., Conte, L., et al. (1998a). New azole antifungals. 2. Synthesis and antifungal activity of heterocyclecarboxamide derivatives of 3-amino-2-aryl-1-azolyl-2-butanol. *J. Med. Chem.* 41, 1855–1868. doi:10.1021/jm970726e.

Bartroli, J., Turmo, E., Algueró, M., Boncompte, E., Vericat, M. L., Conte, L., et al. (1998b). New azole antifungals. 3. Synthesis and antifungal activity of 3-substituted-4(3H)-quinazolinones. *J. Med. Chem.* 41, 1869–1882. doi:10.1021/jm9707277.

Beaulieu, D., Tang, J., Zeckner, D. J., and Parr, T. R. (1993). Correlation of cilofungin in vivo efficacy with its activity against Aspergillus fumigatus (1,3)-beta-D-glucan synthase. *FEMS Microbiol. Lett.* 108, 133–137.

Beauvais, A., Bozza, S., Kniemeyer, O., Formosa, C., Formosa, C., Balloy, V., et al. (2013). Deletion of the α-(1,3)-glucan synthase genes induces a restructuring of the conidial cell wall responsible for the avirulence of Aspergillus fumigatus. *PLoS Pathog.* 9, e1003716. doi:10.1371/journal.ppat.1003716.

Beauvais, A., Maubon, D., Park, S., Morelle, W., Tanguy, M., Huerre, M., et al. (2005). Two alpha(1-3) glucan synthases with different functions in Aspergillus fumigatus. *Appl. Environ. Microbiol.* 71, 1531–1538. doi:10.1128/AEM.71.3.1531-1538.2005.

Beauvais, A., Schmidt, C., Guadagnini, S., Roux, P., Perret, E., Henry, C., et al. (2007). An extracellular matrix glues together the aerial-grown hyphae of Aspergillus fumigatus. *Cell. Microbiol.* 9, 1588–1600. doi:10.1111/j.1462-5822.2007.00895.x.

Becker, M. J., Dams, E. T. M., de Marie, S., Oyen, W. J. G., Boerman, O. C., Fens, M. H. A. M., et al. (2002a). Scintigraphic imaging using 99mTc-labeled PEG liposomes allows early detection of experimental invasive pulmonary aspergillosis in neutropenic rats. *Nucl. Med. Biol.* 29, 177–184.

Becker, M. J., De Marie, S., Fens, M. H. A. M., Haitsma, J. J., Verbrugh, H. A., Lachmann, B., et al. (2006). Pathophysiology of unilateral pulmonary aspergillosis in an experimental rat model. *Med. Mycol. Off. Publ. Int. Soc. Hum. Anim. Mycol.* 44, 133–139.

Becker, M. J., de Marie, S., Fens, M. H. A. M., Hop, W. C. J., Verbrugh, H. A., and Bakker-Woudenberg, I. A. J. M. (2002b). Enhanced antifungal efficacy in experimental invasive pulmonary aspergillosis by combination of AmBisome with Fungizone as assessed by several parameters of antifungal response. *J. Antimicrob. Chemother.* 49, 813–820.

Becker, M. J., de Marie, S., Fens, M. H. A. M., Verbrugh, H. A., and Bakker-Woudenberg, I. A. J. M. (2003). Effect of amphotericin B treatment on kinetics of cytokines and parameters of fungal load in neutropenic rats with invasive pulmonary aspergillosis. *J. Antimicrob. Chemother.* 52, 428–434. doi:10.1093/jac/dkg367.

Becker, M. J., de Marie, S., Willemse, D., Verbrugh, H. A., and Bakker-Woudenberg, I. A. (2000). Quantitative galactomannan detection is superior to PCR in diagnosing and monitoring invasive pulmonary aspergillosis in an experimental rat model. *J. Clin. Microbiol.* 38, 1434–1438.

Bedke, T., Iannitti, R. G., De Luca, A., Giovannini, G., Fallarino, F., Berges, C., et al. (2014). Distinct and complementary roles for Aspergillus fumigatus-specific Tr1 and Foxp3+ regulatory T cells in humans and mice. *Immunol. Cell Biol.* 92, 659–670. doi:10.1038/icb.2014.34.

Behnsen, J., Lessing, F., Schindler, S., Wartenberg, D., Jacobsen, I. D., Thoen, M., et al. (2010). Secreted Aspergillus fumigatus protease Alp1 degrades human complement proteins C3, C4, and C5. *Infect. Immun.* 78, 3585–3594. doi:10.1128/IAI.01353-09.

Bellocchio, S., Gaziano, R., Bozza, S., Rossi, G., Montagnoli, C., Perruccio, K., et al. (2005). Liposomal amphotericin B activates antifungal resistance with reduced toxicity by diverting Toll-like receptor signalling from TLR-2 to TLR-4. *J. Antimicrob. Chemother.* 55, 214–222. doi:10.1093/jac/dkh542.

Bellocchio, S., Montagnoli, C., Bozza, S., Gaziano, R., Rossi, G., Mambula, S. S., et al. (2004a). The contribution of the Toll-like/IL-1 receptor superfamily to innate and adaptive immunity to fungal pathogens in vivo. *J. Immunol. Baltim. Md 1950* 172, 3059–3069.

Bellocchio, S., Moretti, S., Perruccio, K., Fallarino, F., Bozza, S., Montagnoli, C., et al. (2004b). TLRs govern neutrophil activity in aspergillosis. *J. Immunol. Baltim. Md 1950* 173, 7406–7415.

Ben-Ami, R., Albert, N. D., Lewis, R. E., and Kontoyiannis, D. P. (2013). Proangiogenic growth factors potentiate in situ angiogenesis and enhance antifungal drug activity in murine invasive aspergillosis. *J. Infect. Dis.* 207, 1066–1074. doi:10.1093/infdis/jis940.

Ben-Ami, R., and Kontoyiannis, D. P. (2012). A nonlethal murine cutaneous model of invasive aspergillosis. *Methods Mol. Biol. Clifton NJ* 845, 569–582. doi:10.1007/978-1-61779-539-8_42.

Ben-Ami, R., Lewis, R. E., Leventakos, K., and Kontoyiannis, D. P. (2009). Aspergillus fumigatus inhibits angiogenesis through the production of gliotoxin and other secondary metabolites. *Blood* 114, 5393–5399. doi:10.1182/blood-2009-07-231209.

Ben-Ami, R., Lewis, R. E., Leventakos, K., Latgé, J.-P., and Kontoyiannis, D. P. (2010a). Cutaneous model of invasive aspergillosis. *Antimicrob. Agents Chemother.* 54, 1848–1854. doi:10.1128/AAC.01504-09.

Ben-Ami, R., Varga, V., Lewis, R. E., May, G. S., Nierman, W. C., and Kontoyiannis, D. P. (2010b). Characterization of a 5-azacytidine-induced developmental Aspergillus fumigatus variant. *Virulence* 1, 164–173. doi:10.4161/viru.1.3.11750.

Benjamim, C. F., Hogaboam, C. M., Lukacs, N. W., and Kunkel, S. L. (2003). Septic mice are susceptible to pulmonary aspergillosis. *Am. J. Pathol.* 163, 2605–2617. doi:10.1016/S0002-9440(10)63615-2.

Benjamim, C. F., Lundy, S. K., Lukacs, N. W., Hogaboam, C. M., and Kunkel, S. L. (2005). Reversal of long-term sepsis-induced immunosuppression by dendritic cells. *Blood* 105, 3588–3595. doi:10.1182/blood-2004-08-3251.

Bennett, F., Saksena, A. K., Lovey, R. G., Liu, Y.-T., Patel, N. M., Pinto, P., et al. (2006). Hydroxylated analogues of the orally active broad spectrum antifungal, Sch 51048 (1), and the discovery of posaconazole [Sch 56592; 2 or (S,S)-5]. *Bioorg. Med. Chem. Lett.* 16, 186–190. doi:10.1016/j.bmcl.2005.09.031.

Bergmann, A., Hartmann, T., Cairns, T., Bignell, E. M., and Krappmann, S. (2009). A regulator of Aspergillus fumigatus extracellular proteolytic activity is dispensable for virulence. *Infect. Immun.* 77, 4041–4050. doi:10.1128/IAI.00425-09.

Bertuzzi, M., Schrettl, M., Alcazar-Fuoli, L., Cairns, T. C., Muñoz, A., Walker, L. A., et al. (2014). The pH-responsive PacC transcription factor of Aspergillus fumigatus governs epithelial entry and tissue invasion during pulmonary aspergillosis. *PLoS Pathog.* 10, e1004413. doi:10.1371/journal.ppat.1004413.

Bhabhra, R., Miley, M. D., Mylonakis, E., Boettner, D., Fortwendel, J., Panepinto, J. C., et al. (2004). Disruption of the Aspergillus fumigatus gene encoding nucleolar protein CgrA impairs thermotolerant growth and reduces virulence. *Infect. Immun.* 72, 4731–4740. doi:10.1128/IAI.72.8.4731-4740.2004.

Bhatia, S., Fei, M., Yarlagadda, M., Qi, Z., Akira, S., Saijo, S., et al. (2011). Rapid host defense against Aspergillus fumigatus involves alveolar macrophages with a predominance of alternatively activated phenotype. *PloS One* 6, e15943. doi:10.1371/journal.pone.0015943.

Bhatti, M. F., Jamal, A., Petrou, M. A., Cairns, T. C., Bignell, E. M., and Coutts, R. H. A. (2011). The effects of dsRNA mycoviruses on growth and murine virulence of Aspergillus fumigatus. *Fungal Genet. Biol. FG B* 48, 1071–1075. doi:10.1016/j.fgb.2011.07.008.

BitMansour, A., and Brown, J. M. Y. (2002). Prophylactic administration of liposomal amphotericin B is superior to treatment in a murine model of invasive aspergillosis after hematopoietic cell transplantation. *J. Infect. Dis.* 186, 134–137. doi:10.1086/341294.

BitMansour, A., Burns, S. M., Traver, D., Akashi, K., Contag, C. H., Weissman, I. L., et al. (2002). Myeloid progenitors protect against invasive aspergillosis and Pseudomonas aeruginosa infection following hematopoietic stem cell transplantation. *Blood* 100, 4660–4667. doi:10.1182/blood-2002-05-1552.

BitMansour, A., Cao, T. M., Chao, S., Shashidhar, S., and Brown, J. M. Y. (2005). Single infusion of myeloid progenitors reduces death from Aspergillus fumigatus following chemotherapy-induced neutropenia. *Blood* 105, 3535–3537. doi:10.1182/blood-2004-07-2676.

Björgvinsdóttir, H., Ding, C., Pech, N., Gifford, M. A., Li, L. L., and Dinauer, M. C. (1997). Retroviral-mediated gene transfer of gp91phox into bone marrow cells rescues defect in host defense against Aspergillus fumigatus in murine X-linked chronic granulomatous disease. *Blood* 89, 41–48.

Blatzer, M., Barker, B. M., Willger, S. D., Beckmann, N., Blosser, S. J., Cornish, E. J., et al. (2011). SREBP coordinates iron and ergosterol homeostasis to mediate triazole drug and hypoxia responses in the human fungal pathogen Aspergillus fumigatus. *PLoS Genet.* 7, e1002374. doi:10.1371/journal.pgen.1002374.

Bok, J. W., Balajee, S. A., Marr, K. A., Andes, D., Nielsen, K. F., Frisvad, J. C., et al. (2005). LaeA, a regulator of morphogenetic fungal virulence factors. *Eukaryot. Cell* 4, 1574–1582. doi:10.1128/EC.4.9.1574-1582.2005.

Bok, J. W., Chung, D., Balajee, S. A., Marr, K. A., Andes, D., Nielsen, K. F., et al. (2006). GliZ, a Transcriptional Regulator of Gliotoxin Biosynthesis, Contributes to Aspergillus fumigatus Virulence. *Infect. Immun.* 74, 6761–6768. doi:10.1128/IAI.00780-06.

Bom, V. L. P., de Castro, P. A., Winkelströter, L. K., Marine, M., Hori, J. I., Ramalho, L. N. Z., et al. (2015). The Aspergillus fumigatus sitA Phosphatase Homologue Is Important for Adhesion, Cell Wall Integrity, Biofilm Formation, and Virulence. *Eukaryot. Cell* 14, 728–744. doi:10.1128/EC.00008-15.

Bonifazi, P., D’Angelo, C., Zagarella, S., Zelante, T., Bozza, S., De Luca, A., et al. (2010). Intranasally delivered siRNA targeting PI3K/Akt/mTOR inflammatory pathways protects from aspergillosis. *Mucosal Immunol.* 3, 193–205. doi:10.1038/mi.2009.130.

Bonnett, C. R., Cornish, E. J., Harmsen, A. G., and Burritt, J. B. (2006). Early neutrophil recruitment and aggregation in the murine lung inhibit germination of Aspergillus fumigatus Conidia. *Infect. Immun.* 74, 6528–6539. doi:10.1128/IAI.00909-06.

Bowman, J. C., Abruzzo, G. K., Anderson, J. W., Flattery, A. M., Gill, C. J., Pikounis, V. B., et al. (2001). Quantitative PCR assay to measure Aspergillus fumigatus burden in a murine model of disseminated aspergillosis: demonstration of efficacy of caspofungin acetate. *Antimicrob. Agents Chemother.* 45, 3474–3481. doi:10.1128/AAC.45.12.3474-3481.2001.

Bozza, S., Campo, S., Arseni, B., Inforzato, A., Ragnar, L., Bottazzi, B., et al. (2014). PTX3 binds MD-2 and promotes TRIF-dependent immune protection in aspergillosis. *J. Immunol. Baltim. Md 1950* 193, 2340–2348. doi:10.4049/jimmunol.1400814.

Bozza, S., Clavaud, C., Giovannini, G., Fontaine, T., Beauvais, A., Sarfati, J., et al. (2009). Immune sensing of Aspergillus fumigatus proteins, glycolipids, and polysaccharides and the impact on Th immunity and vaccination. *J. Immunol. Baltim. Md 1950* 183, 2407–2414. doi:10.4049/jimmunol.0900961.

Bozza, S., Gaziano, R., Lipford, G. B., Montagnoli, C., Bacci, A., Di Francesco, P., et al. (2002a). Vaccination of mice against invasive aspergillosis with recombinant Aspergillus proteins and CpG oligodeoxynucleotides as adjuvants. *Microbes Infect. Inst. Pasteur* 4, 1281–1290.

Bozza, S., Gaziano, R., Spreca, A., Bacci, A., Montagnoli, C., di Francesco, P., et al. (2002b). Dendritic cells transport conidia and hyphae of Aspergillus fumigatus from the airways to the draining lymph nodes and initiate disparate Th responses to the fungus. *J. Immunol. Baltim. Md 1950* 168, 1362–1371.

Bozza, S., Perruccio, K., Montagnoli, C., Gaziano, R., Bellocchio, S., Burchielli, E., et al. (2003). A dendritic cell vaccine against invasive aspergillosis in allogeneic hematopoietic transplantation. *Blood* 102, 3807–3814. doi:10.1182/blood-2003-03-0748.

Bozza, S., Zelante, T., Moretti, S., Bonifazi, P., DeLuca, A., D’Angelo, C., et al. (2008). Lack of Toll IL-1R8 exacerbates Th17 cell responses in fungal infection. *J. Immunol. Baltim. Md 1950* 180, 4022–4031.

Bretz, C., Gersuk, G., Knoblaugh, S., Chaudhary, N., Randolph-Habecker, J., Hackman, R. C., et al. (2008). MyD88 signaling contributes to early pulmonary responses to Aspergillus fumigatus. *Infect. Immun.* 76, 952–958. doi:10.1128/IAI.00927-07.

Brieland, J. K., Jackson, C., Menzel, F., Loebenberg, D., Cacciapuoti, A., Halpern, J., et al. (2001). Cytokine networking in lungs of immunocompetent mice in response to inhaled Aspergillus fumigatus. *Infect. Immun.* 69, 1554–1560. doi:10.1128/IAI.69.3.1554-1560.2001.

Brock, M., Jouvion, G., Droin-Bergère, S., Dussurget, O., Nicola, M.-A., and Ibrahim-Granet, O. (2008). Bioluminescent Aspergillus fumigatus, a new tool for drug efficiency testing and in vivo monitoring of invasive aspergillosis. *Appl. Environ. Microbiol.* 74, 7023–7035. doi:10.1128/AEM.01288-08.

Brown, J. S., Aufauvre-Brown, A., Brown, J., Jennings, J. M., Arst, H., and Holden, D. W. (2000). Signature-tagged and directed mutagenesis identify PABA synthetase as essential for Aspergillus fumigatus pathogenicity. *Mol. Microbiol.* 36, 1371–1380.

Bruns, S., Kniemeyer, O., Hasenberg, M., Aimanianda, V., Nietzsche, S., Thywissen, A., et al. (2010). Production of extracellular traps against Aspergillus fumigatus in vitro and in infected lung tissue is dependent on invading neutrophils and influenced by hydrophobin RodA. *PLoS Pathog.* 6, e1000873. doi:10.1371/journal.ppat.1000873.

Buskirk, A. D., Green, B. J., Lemons, A. R., Nayak, A. P., Goldsmith, W. T., Kashon, M. L., et al. (2014). A murine inhalation model to characterize pulmonary exposure to dry Aspergillus fumigatus conidia. *PloS One* 9, e109855. doi:10.1371/journal.pone.0109855.

Cacciapuoti, A., Halpern, J., Mendrick, C., Norris, C., Patel, R., and Loebenberg, D. (2006). Interaction between posaconazole and caspofungin in concomitant treatment of mice with systemic Aspergillus infection. *Antimicrob. Agents Chemother.* 50, 2587–2590. doi:10.1128/AAC.00829-05.

Cacciapuoti, A., Loebenberg, D., Corcoran, E., Menzel, F., Moss, E. L., Norris, C., et al. (2000). In vitro and in vivo activities of SCH 56592 (posaconazole), a new triazole antifungal agent, against Aspergillus and Candida. *Antimicrob. Agents Chemother.* 44, 2017–2022.

Caffrey, A. K., Lehmann, M. M., Zickovich, J. M., Espinosa, V., Shepardson, K. M., Watschke, C. P., et al. (2015). IL-1α signaling is critical for leukocyte recruitment after pulmonary Aspergillus fumigatus challenge. *PLoS Pathog.* 11, e1004625. doi:10.1371/journal.ppat.1004625.

Caretti, A., Torelli, R., Perdoni, F., Falleni, M., Tosi, D., Zulueta, A., et al. (2016). Inhibition of ceramide de novo synthesis by myriocin produces the double effect of reducing pathological inflammation and exerting antifungal activity against A. fumigatus airways infection. *Biochim. Biophys. Acta* 1860, 1089–1097. doi:10.1016/j.bbagen.2016.02.014.

Carpenter, K. J., and Hogaboam, C. M. (2005). Immunosuppressive effects of CCL17 on pulmonary antifungal responses during pulmonary invasive aspergillosis. *Infect. Immun.* 73, 7198–7207. doi:10.1128/IAI.73.11.7198-7207.2005.

Carrion, S. de J., Leal, S. M., Ghannoum, M. A., Aimanianda, V., Latgé, J.-P., and Pearlman, E. (2013). The RodA hydrophobin on Aspergillus fumigatus spores masks dectin-1- and dectin-2-dependent responses and enhances fungal survival in vivo. *J. Immunol. Baltim. Md 1950* 191, 2581–2588. doi:10.4049/jimmunol.1300748.

Carvalho, A., De Luca, A., Bozza, S., Cunha, C., D’Angelo, C., Moretti, S., et al. (2012). TLR3 essentially promotes protective class I-restricted memory CD8^+^ T-cell responses to Aspergillus fumigatus in hematopoietic transplanted patients. *Blood* 119, 967–977. doi:10.1182/blood-2011-06-362582.

Casadio, S., Donetti, A., and Coppi, G. (1973). N-trisubstituted methylimidazoles as antifungal agents. *J. Pharm. Sci.* 62, 773–778.

Cenci, E., Mencacci, A., Bacci, A., Bistoni, F., Kurup, V. P., and Romani, L. (2000). T cell vaccination in mice with invasive pulmonary aspergillosis. *J. Immunol. Baltim. Md 1950* 165, 381–388.

Cenci, E., Mencacci, A., Casagrande, A., Mosci, P., Bistoni, F., and Romani, L. (2001). Impaired antifungal effector activity but not inflammatory cell recruitment in interleukin-6-deficient mice with invasive pulmonary aspergillosis. *J. Infect. Dis.* 184, 610–617. doi:10.1086/322793.

Cenci, E., Mencacci, A., Del Sero, G., Bacci, A., Montagnoli, C., d’Ostiani, C. F., et al. (1999). Interleukin-4 causes susceptibility to invasive pulmonary aspergillosis through suppression of protective type I responses. *J. Infect. Dis.* 180, 1957–1968. doi:10.1086/315142.

Cenci, E., Mencacci, A., Fè d’Ostiani, C., Del Sero, G., Mosci, P., Montagnoli, C., et al. (1998). Cytokine- and T helper-dependent lung mucosal immunity in mice with invasive pulmonary aspergillosis. *J. Infect. Dis.* 178, 1750–1760.

Cenci, E., Mencacci, A., Spreca, A., Montagnoli, C., Bacci, A., Perruccio, K., et al. (2002). Protection of killer antiidiotypic antibodies against early invasive aspergillosis in a murine model of allogeneic T-cell-depleted bone marrow transplantation. *Infect. Immun.* 70, 2375–2382.

Cenci, E., Perito, S., Enssle, K. H., Mosci, P., Latgé, J. P., Romani, L., et al. (1997). Th1 and Th2 cytokines in mice with invasive aspergillosis. *Infect. Immun.* 65, 564–570.

Centeno-Lima, S., Silveira, H., Casimiro, C., Aguiar, P., and do Rosário, V. E. (2002). Kinetics of cytokine expression in mice with invasive aspergillosis: lethal infection and protection. *FEMS Immunol. Med. Microbiol.* 32, 167–173.

Chai, L. Y. A., Vonk, A. G., Kullberg, B. J., Verweij, P. E., Verschueren, I., van der Meer, J. W. M., et al. (2011). Aspergillus fumigatus cell wall components differentially modulate host TLR2 and TLR4 responses. *Microbes Infect. Inst. Pasteur* 13, 151–159. doi:10.1016/j.micinf.2010.10.005.

Chakraborty, K. K., and Naik, S. R. (2000). In situ liposomal preparation containing amphotericin B: related toxicity and tissue disposition studies. *Pharm. Dev. Technol.* 5, 543–553. doi:10.1081/PDT-100102037.

Chakraborty, K. K., and Naik, S. R. (2003). Therapeutic and hemolytic evaluation of in-situ liposomal preparation containing amphotericin - beta complexed with different chemically modified beta - cyclodextrins. *J. Pharm. Pharm. Sci. Publ. Can. Soc. Pharm. Sci. Société Can. Sci. Pharm.* 6, 231–237.

Chandenier, J., Bernard, S., Montharu, J., Bailly, E., Fetissof, F., de Monte, M., et al. (2009). The utility of a nebulised intra-tracheal rat model of invasive pulmonary aspergillosis. *Mycoses* 52, 239–245. doi:10.1111/j.1439-0507.2009.01695.x.

Chandrasekar, P. H., Cutright, J. L., and Manavathu, E. K. (2004). Efficacy of voriconazole plus amphotericin B or micafungin in a guinea-pig model of invasive pulmonary aspergillosis. *Clin. Microbiol. Infect. Off. Publ. Eur. Soc. Clin. Microbiol. Infect. Dis.* 10, 925–928. doi:10.1111/j.1469-0691.2004.00958.x.

Chang, Y. C., Tsai, H.-F., Karos, M., and Kwon-Chung, K. J. (2004). THTA, a thermotolerance gene of Aspergillus fumigatus. *Fungal Genet. Biol. FG B* 41, 888–896.

Chaturvedi, A. K., Kavishwar, A., Shiva Keshava, G. B., and Shukla, P. K. (2005). Monoclonal immunoglobulin G1 directed against Aspergillus fumigatus cell wall glycoprotein protects against experimental murine aspergillosis. *Clin. Diagn. Lab. Immunol.* 12, 1063–1068. doi:10.1128/CDLI.12.9.1063-1068.2005.

Chaudhary, N., Datta, K., Askin, F. B., Staab, J. F., and Marr, K. A. (2012). Cystic fibrosis transmembrane conductance regulator regulates epithelial cell response to Aspergillus and resultant pulmonary inflammation. *Am. J. Respir. Crit. Care Med.* 185, 301–310. doi:10.1164/rccm.201106-1027OC.

Chiang, L. Y., Ejzykowicz, D. E., Tian, Z.-Q., Katz, L., and Filler, S. G. (2006). Efficacy of ambruticin analogs in a murine model of invasive pulmonary aspergillosis. *Antimicrob. Agents Chemother.* 50, 3464–3466. doi:10.1128/AAC.00558-06.

Chiang, L. Y., Sheppard, D. C., Gravelat, F. N., Patterson, T. F., and Filler, S. G. (2008). Aspergillus fumigatus stimulates leukocyte adhesion molecules and cytokine production by endothelial cells in vitro and during invasive pulmonary disease. *Infect. Immun.* 76, 3429–3438. doi:10.1128/IAI.01510-07.

Chiller, T. M., Luque, J. C., Sobel, R. A., Farrokhshad, K., Clemons, K. V., and Stevens, D. A. (2002). Development of a murine model of cerebral aspergillosis. *J. Infect. Dis.* 186, 574–577. doi:10.1086/341567.

Chiller, T. M., Sobel, R. A., Luque, J. C., Clemons, K. V., and Stevens, D. A. (2003). Efficacy of amphotericin B or itraconazole in a murine model of central nervous system Aspergillus infection. *Antimicrob. Agents Chemother.* 47, 813–815.

Chowdhry, L., Khan, Z. K., and Kulshrestha, D. K. (1996). Evaluation of himachalol in murine invasive aspergillosis. *Mycoses* 39, 449–452.

Chowdhry, L., Khan, Z. K., and Kulshrestha, D. K. (1997). Comparative in vitro and in vivo evaluation of himachalol in murine invasive aspergillosis. *Indian J. Exp. Biol.* 35, 727–734.

Chung, D., Thammahong, A., Shepardson, K. M., Blosser, S. J., and Cramer, R. A. (2014). Endoplasmic reticulum localized PerA is required for cell wall integrity, azole drug resistance, and virulence in Aspergillus fumigatus. *Mol. Microbiol.* 92, 1279–1298. doi:10.1111/mmi.12626.

Cicogna, C. E., White, M. H., Bernard, E. M., Ishimura, T., Sun, M., Tong, W. P., et al. (1997). Efficacy of prophylactic aerosol amphotericin B lipid complex in a rat model of pulmonary aspergillosis. *Antimicrob. Agents Chemother.* 41, 259–261.

Clark, H. L., Jhingran, A., Sun, Y., Vareechon, C., de Jesus Carrion, S., Skaar, E. P., et al. (2016). Zinc and Manganese Chelation by Neutrophil S100A8/A9 (Calprotectin) Limits Extracellular Aspergillus fumigatus Hyphal Growth and Corneal Infection. *J. Immunol. Baltim. Md 1950* 196, 336–344. doi:10.4049/jimmunol.1502037.

Clark, J. M., Whitney, R. R., Olsen, S. J., George, R. J., Swerdel, M. R., Kunselman, L., et al. (1991). Amphotericin B lipid complex therapy of experimental fungal infections in mice. *Antimicrob. Agents Chemother.* 35, 615–621.

Clemons, K. V., Danielson, M. E., Michel, K. S., Liu, M., Ottoson, N. C., Leonardo, S. M., et al. (2014a). Whole glucan particles as a vaccine against murine aspergillosis. *J. Med. Microbiol.* 63, 1750–1759. doi:10.1099/jmm.0.079681-0.

Clemons, K. V., Espiritu, M., Parmar, R., and Stevens, D. A. (2005). Comparative efficacies of conventional amphotericin b, liposomal amphotericin B (AmBisome), caspofungin, micafungin, and voriconazole alone and in combination against experimental murine central nervous system aspergillosis. *Antimicrob. Agents Chemother.* 49, 4867–4875. doi:10.1128/AAC.49.12.4867-4875.2005.

Clemons, K. V., Grunig, G., Sobel, R. A., Mirels, L. F., Rennick, D. M., and Stevens, D. A. (2000a). Role of IL-10 in invasive aspergillosis: increased resistance of IL-10 gene knockout mice to lethal systemic aspergillosis. *Clin. Exp. Immunol.* 122, 186–191.

Clemons, K. V., Martinez, M., Chen, V., Liu, M., Yoon, H. J., and Stevens, D. A. (2014b). Protection against experimental aspergillosis by heat-killed yeast is not antibody dependent. *Med. Mycol.* 52, 422–426. doi:10.1093/mmy/myt015.

Clemons, K. V., Martinez, M., Tong, A.-J., and Stevens, D. A. (2010). Resistance of MBL gene-knockout mice to experimental systemic aspergillosis. *Immunol. Lett.* 128, 105–107. doi:10.1016/j.imlet.2009.12.021.

Clemons, K. V., Miller, T. K., Selitrennikoff, C. P., and Stevens, D. A. (2002). fos-1, a putative histidine kinase as a virulence factor for systemic aspergillosis. *Med. Mycol.* 40, 259–262.

Clemons, K. V., Parmar, R., Martinez, M., and Stevens, D. A. (2006). Efficacy of Abelcet alone, or in combination therapy, against experimental central nervous system aspergillosis. *J. Antimicrob. Chemother.* 58, 466–469. doi:10.1093/jac/dkl236.

Clemons, K. V., Schwartz, J. A., and Stevens, D. A. (2011). Therapeutic and toxicologic studies in a murine model of invasive pulmonary aspergillosis. *Med. Mycol.* 49, 834–847. doi:10.3109/13693786.2011.577822.

Clemons, K. V., Schwartz, J. A., and Stevens, D. A. (2012). Experimental central nervous system aspergillosis therapy: efficacy, drug levels and localization, immunohistopathology, and toxicity. *Antimicrob. Agents Chemother.* 56, 4439–4449. doi:10.1128/AAC.06015-11.

Clemons, K. V., Sobel, R. A., and Stevens, D. A. (2000b). Toxicity of LY303366, an echinocandin antifungal, in mice pretreated with glucocorticoids. *Antimicrob. Agents Chemother.* 44, 378–381.

Clemons, K. V., and Stevens, D. A. (2004). Comparative efficacies of four amphotericin B formulations--Fungizone, amphotec (Amphocil), AmBisome, and Abelcet--against systemic murine aspergillosis. *Antimicrob. Agents Chemother.* 48, 1047–1050.

Clemons, K. V., and Stevens, D. A. (2006). Efficacy of micafungin alone or in combination against experimental pulmonary aspergillosis. *Med. Mycol.* 44, 69–73.

Cohen, N. R., Tatituri, R. V. V., Rivera, A., Watts, G. F. M., Kim, E. Y., Chiba, A., et al. (2011). Innate recognition of cell wall β-glucans drives invariant natural killer T cell responses against fungi. *Cell Host Microbe* 10, 437–450. doi:10.1016/j.chom.2011.09.011.

Corbel, M. J., and Eades, S. M. (1977). Examination of the effect of age and acquired immunity on the susceptibility of mice to infection with Aspergillus fumigatus. *Mycopathologia* 60, 79–85.

Cornish, E. J., Hurtgen, B. J., McInnerney, K., Burritt, N. L., Taylor, R. M., Jarvis, J. N., et al. (2008). Reduced nicotinamide adenine dinucleotide phosphate oxidase-independent resistance to Aspergillus fumigatus in alveolar macrophages. *J. Immunol. Baltim. Md 1950* 180, 6854–6867.

Cramer, R. A., Gamcsik, M. P., Brooking, R. M., Najvar, L. K., Kirkpatrick, W. R., Patterson, T. F., et al. (2006). Disruption of a nonribosomal peptide synthetase in Aspergillus fumigatus eliminates gliotoxin production. *Eukaryot. Cell* 5, 972–980. doi:10.1128/EC.00049-06.

Cramer, R. A., Perfect, B. Z., Pinchai, N., Park, S., Perlin, D. S., Asfaw, Y. G., et al. (2008). Calcineurin target CrzA regulates conidial germination, hyphal growth, and pathogenesis of Aspergillus fumigatus. *Eukaryot. Cell* 7, 1085–1097. doi:10.1128/EC.00086-08.

Cruz, C. R. Y., Lam, S., Hanley, P. J., Bear, A. S., Langston, C., Cohen, A. J., et al. (2013). Robust T cell responses to aspergillosis in chronic granulomatous disease: implications for immunotherapy. *Clin. Exp. Immunol.* 174, 89–96. doi:10.1111/cei.12156.

Cunha, C., Di Ianni, M., Bozza, S., Giovannini, G., Zagarella, S., Zelante, T., et al. (2010). Dectin-1 Y238X polymorphism associates with susceptibility to invasive aspergillosis in hematopoietic transplantation through impairment of both recipient- and donor-dependent mechanisms of antifungal immunity. *Blood* 116, 5394–5402. doi:10.1182/blood-2010-04-279307.

Cutsem, J. V., Meulemans, L., Gerven, F. V., and Stynen, D. (1993). Effect of tissue invasion and treatment with itraconazole or amphotericin B on galactomannan levels in plasma of guinea-pigs with experimental invasive aspergillosis. *J. Med. Vet. Mycol.* 31, 315–324. doi:10.1080/02681219380000381.

da Silva Ferreira, M. E., Heinekamp, T., Härtl, A., Brakhage, A. A., Semighini, C. P., Harris, S. D., et al. (2007). Functional characterization of the Aspergillus fumigatus calcineurin. *Fungal Genet. Biol. FG B* 44, 219–230. doi:10.1016/j.fgb.2006.08.004.

da Silva Ferreira, M. E., Kress, M. R. V. Z., Savoldi, M., Goldman, M. H. S., Härtl, A., Heinekamp, T., et al. (2006). The akuB(KU80) mutant deficient for nonhomologous end joining is a powerful tool for analyzing pathogenicity in Aspergillus fumigatus. *Eukaryot. Cell* 5, 207–211. doi:10.1128/EC.5.1.207-211.2006.

Dabur, R., Diwedi, S. K., Yadav, V., Mishra, V., Singh, R., Singh, H., et al. (2005). Efficacy of 2-(3,4-dimethyl-2,5-dihydro-1h-pyrrole-2-yl)-1-methylethyl pentanoate in a murine model of invasive aspergillosis. *Antimicrob. Agents Chemother.* 49, 4365–4367. doi:10.1128/AAC.49.10.4365-4367.2005.

Dagenais, T. R. T., Chung, D., Giles, S. S., Hull, C. M., Andes, D., and Keller, N. P. (2008). Defects in conidiophore development and conidium-macrophage interactions in a dioxygenase mutant of Aspergillus fumigatus. *Infect. Immun.* 76, 3214–3220. doi:10.1128/IAI.00009-08.

Dams, E. T., Becker, M. J., Oyen, W. J., Boerman, O. C., Storm, G., Laverman, P., et al. (1999). Scintigraphic imaging of bacterial and fungal infection in granulocytopenic rats. *J. Nucl. Med. Off. Publ. Soc. Nucl. Med.* 40, 2066–2072.

D’Angelo, C., De Luca, A., Zelante, T., Bonifazi, P., Moretti, S., Giovannini, G., et al. (2009). Exogenous pentraxin 3 restores antifungal resistance and restrains inflammation in murine chronic granulomatous disease. *J. Immunol. Baltim. Md 1950* 183, 4609–4618. doi:10.4049/jimmunol.0900345.

Dannaoui, E., Borel, E., Monier, M. F., Piens, M. A., Picot, S., and Persat, F. (2001). Acquired itraconazole resistance in Aspergillus fumigatus. *J. Antimicrob. Chemother.* 47, 333–340.

Dannaoui, E., Borel, E., Persat, F., Monier, M. F., and Piens, M. A. (1999). In-vivo itraconazole resistance of Aspergillus fumigatus in systemic murine aspergillosis. EBGA Network. European research group on Biotypes and Genotypes of Aspergillus fumigatus. *J. Med. Microbiol.* 48, 1087–1093. doi:10.1099/00222615-48-12-1087.

Davies, D. A., and Pope, A. M. (1978). Mycolase, a new kind of systemic antimycotic. *Nature* 273, 235–236.

de Castro, P. A., Chen, C., de Almeida, R. S. C., Freitas, F. Z., Bertolini, M. C., Morais, E. R., et al. (2014a). ChIP-seq reveals a role for CrzA in the Aspergillus fumigatus high-osmolarity glycerol response (HOG) signalling pathway. *Mol. Microbiol.* 94, 655–674. doi:10.1111/mmi.12785.

de Castro, P. A., Chiaratto, J., Winkelströter, L. K., Bom, V. L. P., Ramalho, L. N. Z., Goldman, M. H. S., et al. (2014b). The involvement of the Mid1/Cch1/Yvc1 calcium channels in Aspergillus fumigatus virulence. *PloS One* 9, e103957. doi:10.1371/journal.pone.0103957.

de Gouvêa, P. F., Soriani, F. M., Malavazi, I., Savoldi, M., Goldman, M. H. de S., Loss, O., et al. (2008). Functional characterization of the Aspergillus fumigatusPHO80 homologue. *Fungal Genet. Biol. FG B* 45, 1135–1146. doi:10.1016/j.fgb.2008.04.001.

de Luca, A., Bozza, S., Zelante, T., Zagarella, S., D’Angelo, C., Perruccio, K., et al. (2010). Non-hematopoietic cells contribute to protective tolerance to Aspergillus fumigatus via a TRIF pathway converging on IDO. *Cell. Mol. Immunol.* 7, 459–470. doi:10.1038/cmi.2010.43.

De Luca, A., Iannitti, R. G., Bozza, S., Beau, R., Casagrande, A., D’Angelo, C., et al. (2012). CD4(+) T cell vaccination overcomes defective cross-presentation of fungal antigens in a mouse model of chronic granulomatous disease. *J. Clin. Invest.* 122, 1816–1831. doi:10.1172/JCI60862.

de Luca, A., Smeekens, S. P., Casagrande, A., Iannitti, R., Conway, K. L., Gresnigt, M. S., et al. (2014). IL-1 receptor blockade restores autophagy and reduces inflammation in chronic granulomatous disease in mice and in humans. *Proc. Natl. Acad. Sci. U. S. A.* 111, 3526–3531. doi:10.1073/pnas.1322831111.

de Repentigny, L., Petitbois, S., Boushira, M., Michaliszyn, E., Sénéchal, S., Gendron, N., et al. (1993). Acquired immunity in experimental murine aspergillosis is mediated by macrophages. *Infect. Immun.* 61, 3791–3802.

Defaveri, J., Salazar, M. H., Rinaldi, M. G., and Graybill, J. R. (1990). Pulmonary Aspergillosis in Mice: Treatment with a New Triazole SCH39304. *Am. Rev. Respir. Dis.* 142, 512–515. doi:10.1164/ajrccm/142.3.512.

Del Sero, G., Mencacci, A., Cenci, E., d’Ostiani, C. F., Montagnoli, C., Bacci, A., et al. (1999). Antifungal type 1 responses are upregulated in IL-10-deficient mice. *Microbes Infect. Inst. Pasteur* 1, 1169–1180.

Delmas, G., Park, S., Chen, Z. W., Tan, F., Kashiwazaki, R., Zarif, L., et al. (2002). Efficacy of orally delivered cochleates containing amphotericin B in a murine model of aspergillosis. *Antimicrob. Agents Chemother.* 46, 2704–2707.

D’Enfert, C., Diaquin, M., Delit, A., Wuscher, N., Debeaupuis, J. P., Huerre, M., et al. (1996). Attenuated virulence of uridine-uracil auxotrophs of Aspergillus fumigatus. *Infect. Immun.* 64, 4401–4405.

Denning, D. W., Hall, L., Jackson, M., and Hollis, S. (1995). Efficacy of D0870 compared with those of itraconazole and amphotericin B in two murine models of invasive aspergillosis. *Antimicrob. Agents Chemother.* 39, 1809–1814.

Denning, D. W., Radford, S. A., Oakley, K. L., Hall, L., Johnson, E. M., and Warnock, D. W. (1997a). Correlation between in-vitro susceptibility testing to itraconazole and in-vivo outcome of Aspergillus fumigatus infection. *J. Antimicrob. Chemother.* 40, 401–414.

Denning, D. W., and Stevens, D. A. (1991). Efficacy of cilofungin alone and in combination with amphotericin B in a murine model of disseminated aspergillosis. *Antimicrob. Agents Chemother.* 35, 1329–1333.

Denning, D. W., Venkateswarlu, K., Oakley, K. L., Anderson, M. J., Manning, N. J., Stevens, D. A., et al. (1997b). Itraconazole resistance in Aspergillus fumigatus. *Antimicrob. Agents Chemother.* 41, 1364–1368.

Denning, D. W., and Warn, P. (1999). Dose range evaluation of liposomal nystatin and comparisons with amphotericin B and amphotericin B lipid complex in temporarily neutropenic mice infected with an isolate of Aspergillus fumigatus with reduced susceptibility to amphotericin B. *Antimicrob. Agents Chemother.* 43, 2592–2599.

Dennis, C. G., Greco, W. R., Brun, Y., Youn, R., Slocum, H. K., Bernacki, R. J., et al. (2006). Effect of amphotericin B and micafungin combination on survival, histopathology, and fungal burden in experimental aspergillosis in the p47phox-/- mouse model of chronic granulomatous disease. *Antimicrob. Agents Chemother.* 50, 422–427. doi:10.1128/AAC.50.2.422-427.2006.

Desai, S. K., and Naik, S. R. (2009). Chemotherapeutic activity of liposomal SJA-95: a new polyene macrolide antibiotic in experimental aspergillosis and cryptococcosis. *Biomed. Pharmacother. Bioméd. Pharmacothérapie* 63, 287–292. doi:10.1016/j.biopha.2008.08.017.

Desoubeaux, G., and Chandenier, J. (2012). A nebulized intra-tracheal rat model of invasive pulmonary aspergillosis. *Methods Mol. Biol. Clifton NJ* 845, 511–518. doi:10.1007/978-1-61779-539-8_36.

Desoubeaux, G., Jourdan, M.-L., Valera, L., Jardin, B., Hem, S., Caille, A., et al. (2014). Proteomic demonstration of the recurrent presence of inter-alpha-inhibitor H4 heavy-chain during aspergillosis induced in an animal model. *Int. J. Med. Microbiol. IJMM* 304, 327–338. doi:10.1016/j.ijmm.2013.11.015.

Dhuley, J. N. (1998). Therapeutic efficacy of Ashwagandha against experimental aspergillosis in mice. *Immunopharmacol. Immunotoxicol.* 20, 191–198. doi:10.3109/08923979809034817.

Dinamarco, T. M., Almeida, R. S., de Castro, P. A., Brown, N. A., dos Reis, T. F., Ramalho, L. N. Z., et al. (2012a). Molecular characterization of the putative transcription factor SebA involved in virulence in Aspergillus fumigatus. *Eukaryot. Cell* 11, 518–531. doi:10.1128/EC.00016-12.

Dinamarco, T. M., Freitas, F. Z., Almeida, R. S., Brown, N. A., dos Reis, T. F., Ramalho, L. N. Z., et al. (2012b). Functional characterization of an Aspergillus fumigatus calcium transporter (PmcA) that is essential for fungal infection. *PloS One* 7, e37591. doi:10.1371/journal.pone.0037591.

Dinauer, M. C., Gifford, M. A., Pech, N., Li, L. L., and Emshwiller, P. (2001). Variable correction of host defense following gene transfer and bone marrow transplantation in murine X-linked chronic granulomatous disease. *Blood* 97, 3738–3745.

Dirr, F., Echtenacher, B., Heesemann, J., Hoffmann, P., Ebel, F., and Wagener, J. (2010). AfMkk2 is required for cell wall integrity signaling, adhesion, and full virulence of the human pathogen Aspergillus fumigatus. *Int. J. Med. Microbiol. IJMM* 300, 496–502. doi:10.1016/j.ijmm.2010.03.001.

Dixon, D. M. (1987). In vivo models: evaluating antifungal agents. *Methods Find. Exp. Clin. Pharmacol.* 9, 729–738.

Dixon, D. M., Polak, A., and Walsh, T. J. (1989). Fungus dose-dependent primary pulmonary aspergillosis in immunosuppressed mice. *Infect. Immun.* 57, 1452–1456.

Donat, S., Hasenberg, M., Schäfer, T., Ohlsen, K., Gunzer, M., Einsele, H., et al. (2012). Surface display of Gaussia princeps luciferase allows sensitive fungal pathogen detection during cutaneous aspergillosis. *Virulence* 3, 51–61. doi:10.4161/viru.3.1.18799.

Du, C., Li, R., Ma, S., and Wang, D. (2002). Cloning of Aspergillus fumigatus histidine kinase gene fragment and its expression during invasive infection. *Mycopathologia* 153, 5–10.

Dubourdeau, M., Athman, R., Balloy, V., Huerre, M., Chignard, M., Philpott, D. J., et al. (2006). Aspergillus fumigatus induces innate immune responses in alveolar macrophages through the MAPK pathway independently of TLR2 and TLR4. *J. Immunol. Baltim. Md 1950* 177, 3994–4001.

Dufresne, S. F., Datta, K., Li, X., Dadachova, E., Staab, J. F., Patterson, T. F., et al. (2012). Detection of urinary excreted fungal galactomannan-like antigens for diagnosis of invasive aspergillosis. *PloS One* 7, e42736. doi:10.1371/journal.pone.0042736.

Duong, M., Ouellet, N., Simard, M., Bergeron, Y., Olivier, M., and Bergeron, M. G. (1998). Kinetic study of host defense and inflammatory response to Aspergillus fumigatus in steroid-induced immunosuppressed mice. *J. Infect. Dis.* 178, 1472–1482.

Durrant, C., Tayem, H., Yalcin, B., Cleak, J., Goodstadt, L., de Villena, F. P.-M., et al. (2011). Collaborative Cross mice and their power to map host susceptibility to Aspergillus fumigatus infection. *Genome Res.* 21, 1239–1248. doi:10.1101/gr.118786.110.

Ejzykowicz, D. E., Cunha, M. M., Rozental, S., Solis, N. V., Gravelat, F. N., Sheppard, D. C., et al. (2009). The Aspergillus fumigatus transcription factor Ace2 governs pigment production, conidiation and virulence. *Mol. Microbiol.* 72, 155–169. doi:10.1111/j.1365-2958.2009.06631.x.

Ejzykowicz, D. E., Solis, N. V., Gravelat, F. N., Chabot, J., Li, X., Sheppard, D. C., et al. (2010). Role of Aspergillus fumigatus DvrA in host cell interactions and virulence. *Eukaryot. Cell* 9, 1432–1440. doi:10.1128/EC.00055-10.

El-Muzghi, A. A. M., Mirkov, I., Djokic, J., Popov Aleksandrov, A., Miljkovic, D., Glamoclija, J., et al. (2013). Regional cytokine responses to pulmonary aspergillosis in immunocompetent rats. *Immunobiology* 218, 1514–1523. doi:10.1016/j.imbio.2013.05.007.

Espinosa, V., Jhingran, A., Dutta, O., Kasahara, S., Donnelly, R., Du, P., et al. (2014). Inflammatory monocytes orchestrate innate antifungal immunity in the lung. *PLoS Pathog.* 10, e1003940. doi:10.1371/journal.ppat.1003940.

Evans, S. E., Scott, B. L., Clement, C. G., Larson, D. T., Kontoyiannis, D., Lewis, R. E., et al. (2010a). Stimulated innate resistance of lung epithelium protects mice broadly against bacteria and fungi. *Am. J. Respir. Cell Mol. Biol.* 42, 40–50. doi:10.1165/rcmb.2008-0260OC.

Evans, S. E., Tuvim, M. J., Zhang, J., Larson, D. T., García, C. D., Martinez-Pro, S., et al. (2010b). Host lung gene expression patterns predict infectious etiology in a mouse model of pneumonia. *Respir. Res.* 11, 101. doi:10.1186/1465-9921-11-101.

Fahmy, S. R., Soliman, A. M., and Ali, E. M. (2014). Antifungal and antihepatotoxic effects of sepia ink extract against oxidative stress as a risk factor of invasive pulmonary aspergillosis in neutropenic mice. *Afr. J. Tradit. Complement. Altern. Med. AJTCAM Afr. Netw. Ethnomedicines* 11, 148–159.

Falk, R., Grunwald, J., Hoffman, A., Domb, A. J., and Polacheck, I. (2004). Distribution of amphotericin B-arabinogalactan conjugate in mouse tissue and its therapeutic efficacy against murine aspergillosis. *Antimicrob. Agents Chemother.* 48, 3606–3609. doi:10.1128/AAC.48.9.3606-3609.2004.

Faro-Trindade, I., Willment, J. A., Kerrigan, A. M., Redelinghuys, P., Hadebe, S., Reid, D. M., et al. (2012). Characterisation of innate fungal recognition in the lung. *PloS One* 7, e35675. doi:10.1371/journal.pone.0035675.

Fei, M., Bhatia, S., Oriss, T. B., Yarlagadda, M., Khare, A., Akira, S., et al. (2011). TNF-alpha from inflammatory dendritic cells (DCs) regulates lung IL-17A/IL-5 levels and neutrophilia versus eosinophilia during persistent fungal infection. *Proc. Natl. Acad. Sci. U. S. A.* 108, 5360–5365. doi:10.1073/pnas.1015476108.

Fekkar, A., Pionneau, C., Brossas, J. Y., Marinach-Patrice, C., Snounou, G., Brock, M., et al. (2012). DIGE enables the detection of a putative serum biomarker of fungal origin in a mouse model of invasive aspergillosis. *J. Proteomics* 75, 2536–2549. doi:10.1016/j.jprot.2012.01.040.

Feng, X., Krishnan, K., Richie, D. L., Aimanianda, V., Hartl, L., Grahl, N., et al. (2011). HacA-independent functions of the ER stress sensor IreA synergize with the canonical UPR to influence virulence traits in Aspergillus fumigatus. *PLoS Pathog.* 7, e1002330. doi:10.1371/journal.ppat.1002330.

Fidan, I., Kalkanci, A., Bolat, S., Yesilyurt, E., Erdal, B., Yolbakan, S., et al. (2008). Expression of the surface antigens of lymphocytes and the levels of cytokines in mice infected with Aspergillus fumigatus. *J. Infect. Dev. Ctries.* 2, 34–39.

Fleck, C. B., and Brock, M. (2010). Aspergillus fumigatus catalytic glucokinase and hexokinase: expression analysis and importance for germination, growth, and conidiation. *Eukaryot. Cell* 9, 1120–1135. doi:10.1128/EC.00362-09.

Fontaine, T., Delangle, A., Simenel, C., Coddeville, B., van Vliet, S. J., van Kooyk, Y., et al. (2011). Galactosaminogalactan, a new immunosuppressive polysaccharide of Aspergillus fumigatus. *PLoS Pathog.* 7, e1002372. doi:10.1371/journal.ppat.1002372.

Ford, S., and Friedman, L. (1967). Experimental study of the pathogenicity of aspergilli for mice. *J. Bacteriol.* 94, 928–933.

Fortwendel, J. R., Zhao, W., Bhabhra, R., Park, S., Perlin, D. S., Askew, D. S., et al. (2005). A fungus-specific ras homolog contributes to the hyphal growth and virulence of Aspergillus fumigatus. *Eukaryot. Cell* 4, 1982–1989. doi:10.1128/EC.4.12.1982-1989.2005.

Frosco, M. B., Chase, T., and Macmillan, J. D. (1994). The effect of elastase-specific monoclonal and polyclonal antibodies on the virulence of Aspergillus fumigatus in immunocompromised mice. *Mycopathologia* 125, 65–76.

Fujie, A., Iwamoto, T., Muramatsu, H., Okudaira, T., Nitta, K., Nakanishi, T., et al. (2000a). FR901469, a novel antifungal antibiotic from an unidentified fungus No.11243. I. Taxonomy, fermentation, isolation, physico-chemical properties and biological properties. *J. Antibiot. (Tokyo)* 53, 912–919.

Fujie, A., Iwamoto, T., Muramatsu, H., Okudaira, T., Sato, I., Furuta, T., et al. (2000b). FR901469, a novel antifungal antibiotic from an unidentified fungus No.11243. II. In vitro and in vivo activities. *J. Antibiot. (Tokyo)* 53, 920–927.

Fuller, K. K., Richie, D. L., Feng, X., Krishnan, K., Stephens, T. J., Wikenheiser-Brokamp, K. A., et al. (2011). Divergent Protein Kinase A isoforms co-ordinately regulate conidial germination, carbohydrate metabolism and virulence in Aspergillus fumigatus. *Mol. Microbiol.* 79, 1045–1062. doi:10.1111/j.1365-2958.2010.07509.x.

Furumai, T., Hasegawa, T., Kakushima, M., Suzuki, K., Yamamoto, H., Yamamoto, S., et al. (1993). Pradimicins T1 and T2, new antifungal antibiotics produced by an actinomycete. I. Taxonomy, production, isolation, physico-chemical and biological properties. *J. Antibiot. (Tokyo)* 46, 589–597.

Galiger, C., Brock, M., Jouvion, G., Savers, A., Parlato, M., and Ibrahim-Granet, O. (2013). Assessment of efficacy of antifungals against Aspergillus fumigatus: value of real-time bioluminescence imaging. *Antimicrob. Agents Chemother.* 57, 3046–3059. doi:10.1128/AAC.01660-12.

Gao, J. L., Wynn, T. A., Chang, Y., Lee, E. J., Broxmeyer, H. E., Cooper, S., et al. (1997). Impaired host defense, hematopoiesis, granulomatous inflammation and type 1-type 2 cytokine balance in mice lacking CC chemokine receptor 1. *J. Exp. Med.* 185, 1959–1968.

García, M. E., Blanco, J. L., Caballero, J., and Gargallo-Viola, D. (2002). Anticoagulants interfere with PCR used to diagnose invasive aspergillosis. *J. Clin. Microbiol.* 40, 1567–1568.

García, M. E., Caballero, J., Blanco, I., Cruzado, M., Costas, E., and Blanco, J. L. (2006). Changes in the elastase activity and colonization ability of Aspergillus fumigatus after successive inoculations in mice. *Rev. Iberoam. Micol.* 23, 221–223.

Garlanda, C., Hirsch, E., Bozza, S., Salustri, A., De Acetis, M., Nota, R., et al. (2002). Non-redundant role of the long pentraxin PTX3 in anti-fungal innate immune response. *Nature* 420, 182–186. doi:10.1038/nature01195.

Gavaldà, J., Martín, M.-T., López, P., Gomis, X., Ramírez, J.-L., Rodríguez, D., et al. (2005). Efficacy of nebulized liposomal amphotericin B in treatment of experimental pulmonary aspergillosis. *Antimicrob. Agents Chemother.* 49, 3028–3030. doi:10.1128/AAC.49.7.3028-3030.2005.

Gaziano, R., Bozza, S., Bellocchio, S., Perruccio, K., Montagnoli, C., Pitzurra, L., et al. (2004). Anti-Aspergillus fumigatus efficacy of pentraxin 3 alone and in combination with antifungals. *Antimicrob. Agents Chemother.* 48, 4414–4421. doi:10.1128/AAC.48.11.4414-4421.2004.

Gehrke, A., Heinekamp, T., Jacobsen, I. D., and Brakhage, A. A. (2010). Heptahelical receptors GprC and GprD of Aspergillus fumigatus Are essential regulators of colony growth, hyphal morphogenesis, and virulence. *Appl. Environ. Microbiol.* 76, 3989–3998. doi:10.1128/AEM.00052-10.

Gessner, M. A., Doran, S. F., Yu, Z., Dunaway, C. W., Matalon, S., and Steele, C. (2013). Chlorine gas exposure increases susceptibility to invasive lung fungal infection. *Am. J. Physiol. Lung Cell. Mol. Physiol.* 304, L765-773. doi:10.1152/ajplung.00030.2013.

Gessner, M. A., Werner, J. L., Lilly, L. M., Nelson, M. P., Metz, A. E., Dunaway, C. W., et al. (2012). Dectin-1-dependent interleukin-22 contributes to early innate lung defense against Aspergillus fumigatus. *Infect. Immun.* 80, 410–417. doi:10.1128/IAI.05939-11.

Ghosh, S. K., Mehta, P. K., Patel, J. G., Kashyap, S. K., and Chatterjee, S. K. (1977). Histochemical changes in adrenals of Swiss mice on intraperitoneal administration of Aspergillus spores. *Cell. Mol. Biol. Cyto-Enzymol.* 22, 65–71.

Grahl, N., Dinamarco, T. M., Willger, S. D., Goldman, G. H., and Cramer, R. A. (2012). Aspergillus fumigatus mitochondrial electron transport chain mediates oxidative stress homeostasis, hypoxia responses and fungal pathogenesis. *Mol. Microbiol.* 84, 383–399. doi:10.1111/j.1365-2958.2012.08034.x.

Grahl, N., Puttikamonkul, S., Macdonald, J. M., Gamcsik, M. P., Ngo, L. Y., Hohl, T. M., et al. (2011). In vivo hypoxia and a fungal alcohol dehydrogenase influence the pathogenesis of invasive pulmonary aspergillosis. *PLoS Pathog.* 7, e1002145. doi:10.1371/journal.ppat.1002145.

Gravelat, F. N., Beauvais, A., Liu, H., Lee, M. J., Snarr, B. D., Chen, D., et al. (2013). Aspergillus galactosaminogalactan mediates adherence to host constituents and conceals hyphal β-glucan from the immune system. *PLoS Pathog.* 9, e1003575. doi:10.1371/journal.ppat.1003575.

Gravelat, F. N., Doedt, T., Chiang, L. Y., Liu, H., Filler, S. G., Patterson, T. F., et al. (2008). In Vivo Analysis of Aspergillus fumigatus Developmental Gene Expression Determined by Real-Time Reverse Transcription-PCR. *Infect. Immun.* 76, 3632–3639. doi:10.1128/IAI.01483-07.

Gravelat, F. N., Ejzykowicz, D. E., Chiang, L. Y., Chabot, J. C., Urb, M., Macdonald, K. D., et al. (2010). Aspergillus fumigatus MedA governs adherence, host cell interactions and virulence. *Cell. Microbiol.* 12, 473–488. doi:10.1111/j.1462-5822.2009.01408.x.

Graybill, J. R., and Ahrens, J. (1985). Itraconazole treatment of murine aspergillosis. *Sabouraudia* 23, 219–223.

Graybill, J. R., Bocanegra, R., Gonzalez, G. M., and Najvar, L. K. (2003). Combination antifungal therapy of murine aspergillosis: liposomal amphotericin B and micafungin. *J. Antimicrob. Chemother.* 52, 656–662. doi:10.1093/jac/dkg425.

Graybill, J. R., Bocanegra, R., Najvar, L. K., Loebenberg, D., and Luther, M. F. (1998a). Granulocyte colony-stimulating factor and azole antifungal therapy in murine aspergillosis: role of immune suppression. *Antimicrob. Agents Chemother.* 42, 2467–2473.

Graybill, J. R., Bocanegra, R., Najvar, L. K., Luther, M. F., and Loebenberg, D. (1998b). SCH56592 treatment of murine invasive aspergillosis. *J. Antimicrob. Chemother.* 42, 539–542.

Graybill, J. R., and Kaster, S. R. (1984). Experimental murine aspergillosis. Comparison of amphotericin B and a new polyene antifungal drug, SCH 28191. *Am. Rev. Respir. Dis.* 129, 292–295.

Graybill, J. R., Kaster, S. R., and Drutz, D. J. (1983). Treatment of experimental murine aspergillosis with BAY n7133. *J. Infect. Dis.* 148, 898–906.

Gresnigt, M. S., Bozza, S., Becker, K. L., Joosten, L. A. B., Abdollahi-Roodsaz, S., van der Berg, W. B., et al. (2014). A polysaccharide virulence factor from Aspergillus fumigatus elicits anti-inflammatory effects through induction of Interleukin-1 receptor antagonist. *PLoS Pathog.* 10, e1003936. doi:10.1371/journal.ppat.1003936.

Grimm, M. J., Vethanayagam, R. R., Almyroudis, N. G., Dennis, C. G., Khan, A. N. H., D’Auria, A. C., et al. (2013). Monocyte- and macrophage-targeted NADPH oxidase mediates antifungal host defense and regulation of acute inflammation in mice. *J. Immunol. Baltim. Md 1950* 190, 4175–4184. doi:10.4049/jimmunol.1202800.

Guo, H., Gao, J., and Wu, X. (2012). Toll-like receptor 2 siRNA suppresses corneal inflammation and attenuates Aspergillus fumigatus keratitis in rats. *Immunol. Cell Biol.* 90, 352–357. doi:10.1038/icb.2011.49.

Gyotoku, H., Izumikawa, K., Ikeda, H., Takazono, T., Morinaga, Y., Nakamura, S., et al. (2012). A case of bronchial aspergillosis caused by Aspergillus udagawae and its mycological features. *Med. Mycol.* 50, 631–636. doi:10.3109/13693786.2011.639036.

Habicht, J. M., Preiss, M., Passweg, J., Dalquen, P., Matt, P., Adler, H., et al. (2002). Invasive pulmonary aspergillosis: effects of early resection in a neutropenic rat model. *Eur. J. Cardio-Thorac. Surg. Off. J. Eur. Assoc. Cardio-Thorac. Surg.* 22, 728–732.

Hachem, R., Bahna, P., Hanna, H., Stephens, L. C., and Raad, I. (2006). EDTA as an adjunct antifungal agent for invasive pulmonary aspergillosis in a rodent model. *Antimicrob. Agents Chemother.* 50, 1823–1827. doi:10.1128/AAC.50.5.1823-1827.2006.

Han, K.-H., Chun, Y.-H., Figueiredo, B. de C. P., Soriani, F. M., Savoldi, M., Almeida, A., et al. (2010). The conserved and divergent roles of carbonic anhydrases in the filamentous fungi Aspergillus fumigatus and Aspergillus nidulans. *Mol. Microbiol.* 75, 1372–1388. doi:10.1111/j.1365-2958.2010.07058.x.

Hanazawa, R., Murayama, S. Y., and Yamaguchi, H. (2000). In-situ detection of Aspergillus fumigatus. *J. Med. Microbiol.* 49, 285–290. doi:10.1099/0022-1317-49-3-285.

Hanson, L. H., Clemons, K. V., Denning, D. W., and Stevens, D. A. (1995). Efficacy of oral saperconazole in systemic murine aspergillosis. *J. Med. Vet. Mycol. Bi-Mon. Publ. Int. Soc. Hum. Anim. Mycol.* 33, 311–317.

Harindran, J., Chakraborty, K. K., and Naik, S. R. (1999). Preparation, relative toxicity and therapeutic efficacy in mice and rats of liposomal HA-1-92, a new oxohexaene polyene macrolide antibiotic. *J. Pharm. Pharmacol.* 51, 771–776.

Hartigan, A. J., Kallal, L. E., and Hogaboam, C. M. (2010). CCR7 impairs hematopoiesis after hematopoietic stem cell transplantation increasing susceptibility to invasive aspergillosis. *Blood* 116, 5383–5393. doi:10.1182/blood-2010-01-265454.

Hartigan, A. J., Westwick, J., Jarai, G., and Hogaboam, C. M. (2009). CCR7 deficiency on dendritic cells enhances fungal clearance in a murine model of pulmonary invasive aspergillosis. *J. Immunol. Baltim. Md 1950* 183, 5171–5179. doi:10.4049/jimmunol.0901027.

Hartmann, T., Cairns, T. C., Olbermann, P., Morschhäuser, J., Bignell, E. M., and Krappmann, S. (2011). Oligopeptide transport and regulation of extracellular proteolysis are required for growth of Aspergillus fumigatus on complex substrates but not for virulence. *Mol. Microbiol.* 82, 917–935. doi:10.1111/j.1365-2958.2011.07868.x.

Hasenberg, M., Köhler, A., Bonifatius, S., Jeron, A., and Gunzer, M. (2011). Direct observation of phagocytosis and NET-formation by neutrophils in infected lungs using 2-photon microscopy. *J. Vis. Exp. JoVE*. doi:10.3791/2659.

Hashiguchi, K., Niki, Y., and Soejima, R. (1994). Cyclophosphamide induces false-positive results in detection of aspergillus antigen in urine. *Chest* 105, 975–976.

Hashimoto, A., Yamakami, Y., Kamberi, P., Yamagata, E., Karashima, R., Nagaoka, H., et al. (1998). Comparison of PCR, (1-->3)-beta-D-glucan and galactomannan assays in sera of rats with experimental invasive aspergillosis. *J. Clin. Lab. Anal.* 12, 257–262.

Hata, K., Horii, T., Miyazaki, M., Watanabe, N.-A., Okubo, M., Sonoda, J., et al. (2011). Efficacy of oral E1210, a new broad-spectrum antifungal with a novel mechanism of action, in murine models of candidiasis, aspergillosis, and fusariosis. *Antimicrob. Agents Chemother.* 55, 4543–4551. doi:10.1128/AAC.00366-11.

Hata, K., Kimura, J., Miki, H., Toyosawa, T., Moriyama, M., and Katsu, K. (1996a). Efficacy of ER-30346, a novel oral triazole antifungal agent, in experimental models of aspergillosis, candidiasis, and cryptococcosis. *Antimicrob. Agents Chemother.* 40, 2243–2247.

Hata, K., Kimura, J., Miki, H., Toyosawa, T., Nakamura, T., and Katsu, K. (1996b). In vitro and in vivo antifungal activities of ER-30346, a novel oral triazole with a broad antifungal spectrum. *Antimicrob. Agents Chemother.* 40, 2237–2242.

Hayashi, R., Kitamoto, N., Iizawa, Y., Ichikawa, T., Itoh, K., Kitazaki, T., et al. (2002). Efficacy of TAK-457, a novel intravenous triazole, against invasive pulmonary Aspergillosis in neutropenic mice. *Antimicrob. Agents Chemother.* 46, 283–287.

Hector, R. F., and Yee, E. (1990). Evaluation of Bay R 3783 in rodent models of superficial and systemic candidiasis, meningeal cryptococcosis, and pulmonary aspergillosis. *Antimicrob. Agents Chemother.* 34, 448–454.

Hector, R. F., Yee, E., and Collins, M. S. (1990). Use of DBA/2N mice in models of systemic candidiasis and pulmonary and systemic aspergillosis. *Infect. Immun.* 58, 1476–1478.

Heesemann, L., Kotz, A., Echtenacher, B., Broniszewska, M., Routier, F., Hoffmann, P., et al. (2011). Studies on galactofuranose-containing glycostructures of the pathogenic mold Aspergillus fumigatus. *Int. J. Med. Microbiol. IJMM* 301, 523–530. doi:10.1016/j.ijmm.2011.02.003.

Hein, K. Z., Takahashi, H., Tsumori, T., Yasui, Y., Nanjoh, Y., Toga, T., et al. (2015). Disulphide-reduced psoriasin is a human apoptosis-inducing broad-spectrum fungicide. *Proc. Natl. Acad. Sci. U. S. A.* 112, 13039–13044. doi:10.1073/pnas.1511197112.

Hensel, M., Arst, H. N., Aufauvre-Brown, A., and Holden, D. W. (1998). The role of the Aspergillus fumigatus areA gene in invasive pulmonary aspergillosis. *Mol. Gen. Genet. MGG* 258, 553–557.

Herbst, S., Shah, A., Carby, M., Chusney, G., Kikkeri, N., Dorling, A., et al. (2013). A new and clinically relevant murine model of solid-organ transplant aspergillosis. *Dis. Model. Mech.* 6, 643–651. doi:10.1242/dmm.010330.

Herbst, S., Shah, A., Mazon Moya, M., Marzola, V., Jensen, B., Reed, A., et al. (2015). Phagocytosis-dependent activation of a TLR9-BTK-calcineurin-NFAT pathway co-ordinates innate immunity to Aspergillus fumigatus. *EMBO Mol. Med.* 7, 240–258. doi:10.15252/emmm.201404556.

High, K. P., and Washburn, R. G. (1997). Invasive aspergillosis in mice immunosuppressed with cyclosporin A, tacrolimus (FK506), or sirolimus (rapamycin). *J. Infect. Dis.* 175, 222–225.

Hissen, A. H. T., Wan, A. N. C., Warwas, M. L., Pinto, L. J., and Moore, M. M. (2005). The Aspergillus fumigatus siderophore biosynthetic gene sidA, encoding L-ornithine N5-oxygenase, is required for virulence. *Infect. Immun.* 73, 5493–5503. doi:10.1128/IAI.73.9.5493-5503.2005.

Hohl, T. M., Rivera, A., Lipuma, L., Gallegos, A., Shi, C., Mack, M., et al. (2009). Inflammatory monocytes facilitate adaptive CD4 T cell responses during respiratory fungal infection. *Cell Host Microbe* 6, 470–481. doi:10.1016/j.chom.2009.10.007.

Hohl, T. M., Van Epps, H. L., Rivera, A., Morgan, L. A., Chen, P. L., Feldmesser, M., et al. (2005). Aspergillus fumigatus triggers inflammatory responses by stage-specific beta-glucan display. *PLoS Pathog.* 1, e30. doi:10.1371/journal.ppat.0010030.

Hood, J. R., Burton, D., Wilkinson, J. M., and Cavanagh, H. M. A. (2010). Antifungal activity of Leptospermum petersonii oil volatiles against Aspergillus spp. in vitro and in vivo. *J. Antimicrob. Chemother.* 65, 285–288. doi:10.1093/jac/dkp400.

Hooper, D. G., Bolton, V. E., Sutton, J. S., Guilford, F. T., Straus, D. C., Najvar, L. K., et al. (2012). Assessment of Aspergillus fumigatus in guinea pig bronchoalveolar lavages and pulmonary tissue by culture and realtime polymerase chain reaction studies. *Int. J. Mol. Sci.* 13, 726–736. doi:10.3390/ijms13010726.

Howard, S. J., Lestner, J. M., Sharp, A., Gregson, L., Goodwin, J., Slater, J., et al. (2011). Pharmacokinetics and pharmacodynamics of posaconazole for invasive pulmonary aspergillosis: clinical implications for antifungal therapy. *J. Infect. Dis.* 203, 1324–1332. doi:10.1093/infdis/jir023.

Hsu, J. L., Khan, M. A., Sobel, R. A., Jiang, X., Clemons, K. V., Nguyen, T. T., et al. (2013). Aspergillus fumigatus invasion increases with progressive airway ischemia. *PloS One* 8, e77136. doi:10.1371/journal.pone.0077136.

Hu, W., Sillaots, S., Lemieux, S., Davison, J., Kauffman, S., Breton, A., et al. (2007). Essential gene identification and drug target prioritization in Aspergillus fumigatus. *PLoS Pathog.* 3, e24. doi:10.1371/journal.ppat.0030024.

Huang, W., Ling, S., Jia, X., Lin, B., Huang, X., Zhong, J., et al. (2014). Tacrolimus (FK506) suppresses TREM-1 expression at an early but not at a late stage in a murine model of fungal keratitis. *PloS One* 9, e114386. doi:10.1371/journal.pone.0114386.

Huber, F., and Bignell, E. (2014). Distribution, expression and expansion of Aspergillus fumigatus LINE-like retrotransposon populations in clinical and environmental isolates. *Fungal Genet. Biol. FG B* 64, 36–44. doi:10.1016/j.fgb.2014.01.002.

Hummel, M., Baust, C., Kretschmar, M., Nichterlein, T., Schleiermacher, D., Spiess, B., et al. (2004). Detection of Aspergillus DNA by a nested PCR assay is superior to blood culture in an experimental murine model of invasive aspergillosis. *J. Med. Microbiol.* 53, 803–806. doi:10.1099/jmm.0.45545-0.

Iannitti, R. G., Casagrande, A., De Luca, A., Cunha, C., Sorci, G., Riuzzi, F., et al. (2013). Hypoxia promotes danger-mediated inflammation via receptor for advanced glycation end products in cystic fibrosis. *Am. J. Respir. Crit. Care Med.* 188, 1338–1350. doi:10.1164/rccm.201305-0986OC.

Iannitti, R. G., Napolioni, V., Oikonomou, V., De Luca, A., Galosi, C., Pariano, M., et al. (2016). IL-1 receptor antagonist ameliorates inflammasome-dependent inflammation in murine and human cystic fibrosis. *Nat. Commun.* 7, 10791. doi:10.1038/ncomms10791.

Ibrahim, A. S., Gebremariam, T., French, S. W., Edwards, J. E., and Spellberg, B. (2010). The iron chelator deferasirox enhances liposomal amphotericin B efficacy in treating murine invasive pulmonary aspergillosis. *J. Antimicrob. Chemother.* 65, 289–292. doi:10.1093/jac/dkp426.

Ibrahim, A. S., Gebremariam, T., Luo, G., Fu, Y., French, S. W., Edwards, J. E., et al. (2011). Combination therapy of murine mucormycosis or aspergillosis with iron chelation, polyenes, and echinocandins. *Antimicrob. Agents Chemother.* 55, 1768–1770. doi:10.1128/AAC.01577-10.

Ibrahim-Granet, O., Dubourdeau, M., Latgé, J.-P., Ave, P., Huerre, M., Brakhage, A. A., et al. (2008). Methylcitrate synthase from Aspergillus fumigatus is essential for manifestation of invasive aspergillosis. *Cell. Microbiol.* 10, 134–148. doi:10.1111/j.1462-5822.2007.1025.x.

Ibrahim-Granet, O., Jouvion, G., Hohl, T. M., Droin-Bergère, S., Philippart, F., Kim, O. Y., et al. (2010). In vivo bioluminescence imaging and histopathopathologic analysis reveal distinct roles for resident and recruited immune effector cells in defense against invasive aspergillosis. *BMC Microbiol.* 10, 105. doi:10.1186/1471-2180-10-105.

Ichikawa, T., Kitazaki, T., Matsushita, Y., Yamada, M., Hayashi, R., Yamaguchi, M., et al. (2001). Optically active antifungal azoles. XII. Synthesis and antifungal activity of the water-soluble prodrugs of 1-[(1R,2R)-2-(2,4-difluorophenyl)-2-hydroxy-1-methyl-3-(1H-1,2,4-triazol-1-yl)propyl]-3-[4-(1H-1-tetrazolyl)phenyl]-2-imidazolidinone. *Chem. Pharm. Bull. (Tokyo)* 49, 1102–1109.

Ikeda, F., Wakai, Y., Matsumoto, S., Maki, K., Watabe, E., Tawara, S., et al. (2000). Efficacy of FK463, a new lipopeptide antifungal agent, in mouse models of disseminated candidiasis and aspergillosis. *Antimicrob. Agents Chemother.* 44, 614–618.

Imai, J. K., Singh, G., Clemons, K. V., and Stevens, D. A. (2004). Efficacy of posaconazole in a murine model of central nervous system aspergillosis. *Antimicrob. Agents Chemother.* 48, 4063–4066. doi:10.1128/AAC.48.10.4063-4066.2004.

Imai, J., Singh, G., Fernandez, B., Clemons, K. V., and Stevens, D. A. (2005). Efficacy of Abelcet and caspofungin, alone or in combination, against CNS aspergillosis in a murine model. *J. Antimicrob. Chemother.* 56, 166–171. doi:10.1093/jac/dki178.

Italia, J. L., Sharp, A., Carter, K. C., Warn, P., and Kumar, M. N. V. R. (2011). Peroral amphotericin B polymer nanoparticles lead to comparable or superior in vivo antifungal activity to that of intravenous Ambisome® or Fungizone^TM^. *PloS One* 6, e25744. doi:10.1371/journal.pone.0025744.

Ito, J. I., and Lyons, J. M. (2002). Vaccination of corticosteroid immunosuppressed mice against invasive pulmonary aspergillosis. *J. Infect. Dis.* 186, 869–871. doi:10.1086/342509.

Ito, J. I., Lyons, J. M., Hong, T. B., Tamae, D., Liu, Y.-K., Wilczynski, S. P., et al. (2006). Vaccinations with recombinant variants of Aspergillus fumigatus allergen Asp f 3 protect mice against invasive aspergillosis. *Infect. Immun.* 74, 5075–5084. doi:10.1128/IAI.00815-06.

Jahn, B., Koch, A., Schmidt, A., Wanner, G., Gehringer, H., Bhakdi, S., et al. (1997). Isolation and characterization of a pigmentless-conidium mutant of Aspergillus fumigatus with altered conidial surface and reduced virulence. *Infect. Immun.* 65, 5110–5117.

Jambunathan, K., Watson, D. S., Najvar, L. K., Wiederhold, N. P., Kirkpatrick, W. R., Patterson, T. F., et al. (2013). Prolyl endopeptidase activity in bronchoalveolar lavage fluid: a novel diagnostic biomarker in a guinea pig model of invasive pulmonary aspergillosis. *Med. Mycol.* 51, 592–602. doi:10.3109/13693786.2012.761360.

Jaton-Ogay, K., Paris, S., Huerre, M., Quadroni, M., Falchetto, R., Togni, G., et al. (1994). Cloning and disruption of the gene encoding an extracellular metalloprotease of Aspergillus fumigatus. *Mol. Microbiol.* 14, 917–928.

Jensen, H. E., Aalbaek, B., Hau, J., and Latgé, J. P. (1996a). Detection of galactomannan and complement activation in the pregnant mouse during experimental systemic aspergillosis. *APMIS Acta Pathol. Microbiol. Immunol. Scand.* 104, 926–932.

Jensen, H. E., Aalbaek, B., Lind, P., Frandsen, P. L., Krogh, H. V., and Stynen, D. (1993a). Enzyme immunohistochemistry with mono- and polyclonal antibodies in the pathological diagnosis of systemic bovine mycoses. *APMIS Acta Pathol. Microbiol. Immunol. Scand.* 101, 505–516.

Jensen, H. E., Halbaek, B., Lind, P., Krogh, H. V., and Frandsen, P. L. (1996b). Development of murine monoclonal antibodies for the immunohistochemical diagnosis of systemic bovine aspergillosis. *J. Vet. Diagn. Investig. Off. Publ. Am. Assoc. Vet. Lab. Diagn. Inc* 8, 68–75.

Jensen, H. E., and Hau, J. (1990a). A murine model for the study of the impact of Aspergillus fumigatus inoculation on the foeto-placental unit. *Mycopathologia* 112, 11–18.

Jensen, H. E., and Hau, J. (1990b). Murine mycotic placentitis produced by intravenous inoculation of conidia from Aspergillus fumigatus. *Vivo Athens Greece* 4, 247–252.

Jensen, H. E., and Schønheyder, H. (1989). Immunofluorescence staining of hyphae in the histopathological diagnosis of mycoses in cattle. *J. Med. Vet. Mycol. Bi-Mon. Publ. Int. Soc. Hum. Anim. Mycol.* 27, 33–44.

Jensen, H. E., and Schønheyder, H. (1993). Experimental murine mycotic placentitis and abortion. A potent animal model (short communication). *J. Exp. Anim. Sci.* 35, 155–160.

Jensen, H. E., Stynen, D., Sarfati, J., and Latgé, J. P. (1993b). Detection of galactomannan and the 18 kDa antigen from Aspergillus fumigatus in serum and urine from cattle with systemic aspergillosis. *Zentralblatt Für Veterinärmedizin Reihe B J. Vet. Med. Ser. B* 40, 397–408.

Jhingran, A., Kasahara, S., Shepardson, K. M., Junecko, B. A. F., Heung, L. J., Kumasaka, D. K., et al. (2015). Compartment-specific and sequential role of MyD88 and CARD9 in chemokine induction and innate defense during respiratory fungal infection. *PLoS Pathog.* 11, e1004589. doi:10.1371/journal.ppat.1004589.

Jhingran, A., Mar, K. B., Kumasaka, D. K., Knoblaugh, S. E., Ngo, L. Y., Segal, B. H., et al. (2012). Tracing conidial fate and measuring host cell antifungal activity using a reporter of microbial viability in the lung. *Cell Rep.* 2, 1762–1773. doi:10.1016/j.celrep.2012.10.026.

Jiang, B., Xu, D., Allocco, J., Parish, C., Davison, J., Veillette, K., et al. (2008). PAP inhibitor with in vivo efficacy identified by Candida albicans genetic profiling of natural products. *Chem. Biol.* 15, 363–374. doi:10.1016/j.chembiol.2008.02.016.

Jiang, H., Shen, Y., Liu, W., and Lu, L. (2014). Deletion of the putative stretch-activated ion channel Mid1 is hypervirulent in Aspergillus fumigatus. *Fungal Genet. Biol. FG B* 62, 62–70. doi:10.1016/j.fgb.2013.11.003.

Jiang, N., Zhao, G., Lin, J., Hu, L., Che, C., Li, C., et al. (2015). Indoleamine 2,3-Dioxygenase Is Involved in the Inflammation Response of Corneal Epithelial Cells to Aspergillus fumigatus Infections. *PloS One* 10, e0137423. doi:10.1371/journal.pone.0137423.

Jiang, X., Hsu, J. L., Tian, W., Yuan, K., Olcholski, M., Perez, V. de J., et al. (2013). Tie2-dependent VHL knockdown promotes airway microvascular regeneration and attenuates invasive growth of Aspergillus fumigatus. *J. Mol. Med. Berl. Ger.* 91, 1081–1093. doi:10.1007/s00109-013-1063-8.

Jie Zhao, null, Wu, X., and Yu, F.-S. X. (2009). Activation of Toll-like receptors 2 and 4 in Aspergillus fumigatus keratitis. *Innate Immun.* 15, 155–168. doi:10.1177/1753425908101521.

Johnson, C. P., Edmiston, C. E., Zhu, Y.-R., Adams, M. B., Roza, A. M., and Kurup, V. (2005). A murine model of invasive aspergillosis: variable benefit of interferon-gamma administration under in vitro and in vivo conditions. *Surg. Infect.* 6, 397–407. doi:10.1089/sur.2005.6.397.

Johnson, E. M., Oakley, K. L., Radford, S. A., Moore, C. B., Warn, P., Warnock, D. W., et al. (2000). Lack of correlation of in vitro amphotericin B susceptibility testing with outcome in a murine model of Aspergillus infection. *J. Antimicrob. Chemother.* 45, 85–93.

Jouvion, G., Brock, M., Droin-Bergère, S., and Ibrahim-Granet, O. (2012). Duality of liver and kidney lesions after systemic infection of immunosuppressed and immunocompetent mice with Aspergillus fumigatus. *Virulence* 3, 43–50. doi:10.4161/viru.3.1.18654.

Jung, S. H., Lim, D. H., Jung, S. H., Lee, J. E., Jeong, K.-S., Seong, H., et al. (2009). Amphotericin B-entrapping lipid nanoparticles and their in vitro and in vivo characteristics. *Eur. J. Pharm. Sci. Off. J. Eur. Fed. Pharm. Sci.* 37, 313–320. doi:10.1016/j.ejps.2009.02.021.

Juvvadi, P. R., Gehrke, C., Fortwendel, J. R., Lamoth, F., Soderblom, E. J., Cook, E. C., et al. (2013). Phosphorylation of Calcineurin at a novel serine-proline rich region orchestrates hyphal growth and virulence in Aspergillus fumigatus. *PLoS Pathog.* 9, e1003564. doi:10.1371/journal.ppat.1003564.

Kai, H., Yamashita, M., Nakamura, I., Yoshikawa, K., Nitta, K., Watanabe, M., et al. (2013). Synergistic antifungal activity of KB425796-C in combination with micafungin against Aspergillus fumigatus and its efficacy in murine infection models. *J. Antibiot. (Tokyo)* 66, 479–484. doi:10.1038/ja.2013.57.

Kakeya, H., Miyazaki, Y., Senda, H., Kobayashi, T., Seki, M., Izumikawa, K., et al. (2008). Efficacy of SPK-843, a novel polyene antifungal, in comparison with amphotericin B, liposomal amphotericin B, and micafungin against murine pulmonary aspergillosis. *Antimicrob. Agents Chemother.* 52, 1868–1870. doi:10.1128/AAC.01369-07.

Kapp, K., Prüfer, S., Michel, C. S., Habermeier, A., Luckner-Minden, C., Giese, T., et al. (2014). Granulocyte functions are independent of arginine availability. *J. Leukoc. Biol.* 96, 1047–1053. doi:10.1189/jlb.3AB0214-082R.

Karki, R., Man, S. M., Malireddi, R. K. S., Gurung, P., Vogel, P., Lamkanfi, M., et al. (2015). Concerted activation of the AIM2 and NLRP3 inflammasomes orchestrates host protection against Aspergillus infection. *Cell Host Microbe* 17, 357–368. doi:10.1016/j.chom.2015.01.006.

Kasahara, S., Jhingran, A., Dhingra, S., Salem, A., Cramer, R. A., and Hohl, T. M. (2016). Role of Granulocyte-Macrophage Colony-Stimulating Factor Signaling in Regulating Neutrophil Antifungal Activity and the Oxidative Burst During Respiratory Fungal Challenge. *J. Infect. Dis.* 213, 1289–1298. doi:10.1093/infdis/jiw054.

Kaur, S., Gupta, V. K., Thiel, S., Sarma, P. U., and Madan, T. (2007). Protective role of mannan-binding lectin in a murine model of invasive pulmonary aspergillosis. *Clin. Exp. Immunol.* 148, 382–389. doi:10.1111/j.1365-2249.2007.03351.x.

Keller, S., Macheleidt, J., Scherlach, K., Schmaler-Ripcke, J., Jacobsen, I. D., Heinekamp, T., et al. (2011). Pyomelanin formation in Aspergillus fumigatus requires HmgX and the transcriptional activator HmgR but is dispensable for virulence. *PloS One* 6, e26604. doi:10.1371/journal.pone.0026604.

Kerr, S. C., Fischer, G. J., Sinha, M., McCabe, O., Palmer, J. M., Choera, T., et al. (2016). FleA Expression in Aspergillus fumigatus Is Recognized by Fucosylated Structures on Mucins and Macrophages to Prevent Lung Infection. *PLoS Pathog.* 12, e1005555. doi:10.1371/journal.ppat.1005555.

Khalaj, V., Azizi, M., Enayati, S., Khorasanizadeh, D., and Ardakani, E. M. (2012). NCE102 homologue in Aspergillus fumigatus is required for normal sporulation, not hyphal growth or pathogenesis. *FEMS Microbiol. Lett.* 329, 138–145. doi:10.1111/j.1574-6968.2012.02513.x.

Khan, M. A., Ahmad, N., Moin, S., Mannan, A., Wajahul, H., Pasha, S. T., et al. (2005). Tuftsin-mediated immunoprophylaxis against an isolate of Aspergillus fumigatus shows less in vivo susceptibility to amphotericin B. *FEMS Immunol. Med. Microbiol.* 44, 269–276. doi:10.1016/j.femsim.2004.12.013.

Khan, M. A., Faisal, S. M., and Mohammad, O. (2006). Safety, efficacy and pharmacokinetics of tuftsin-loaded nystatin liposomes in murine model. *J. Drug Target.* 14, 233–241. doi:10.1080/10611860600720384.

Khan, M. A., Nasti, T. H., Saima, K., Mallick, A. I., Firoz, A., Wajahul, H., et al. (2004). Co-administration of immunomodulator tuftsin and liposomised nystatin can combat less susceptible Candida albicans infection in temporarily neutropenic mice. *FEMS Immunol. Med. Microbiol.* 41, 249–258. doi:10.1016/j.femsim.2004.03.011.

Khan, Z. U., Ahmad, S., and Theyyathel, A. M. (2008). Detection of Aspergillus fumigatus-specific DNA, (1-3)-beta-D-glucan and galactomannan in serum and bronchoalveolar lavage specimens of experimentally infected rats. *Mycoses* 51, 129–135. doi:10.1111/j.1439-0507.2007.01461.x.

Khosravi, A. R., Mahdavi Omran, S., Shokri, H., Lotfi, A., and Moosavi, Z. (2012). Importance of elastase production in development of invasive aspergillosis. *J. Mycol. Médicale* 22, 167–172. doi:10.1016/j.mycmed.2012.03.002.

Kirkpatrick, W. R., Coco, B. J., and Patterson, T. F. (2006). Sequential or combination antifungal therapy with voriconazole and liposomal amphotericin B in a Guinea pig model of invasive aspergillosis. *Antimicrob. Agents Chemother.* 50, 1567–1569. doi:10.1128/AAC.50.4.1567-1569.2006.

Kirkpatrick, W. R., McAtee, R. K., Fothergill, A. W., Rinaldi, M. G., and Patterson, T. F. (2000). Efficacy of voriconazole in a guinea pig model of disseminated invasive aspergillosis. *Antimicrob. Agents Chemother.* 44, 2865–2868.

Kirkpatrick, W. R., Najvar, L. K., Vallor, A. C., Wiederhold, N. P., Bocanegra, R., Pfeiffer, J., et al. (2012). Prophylactic efficacy of single dose pulmonary administration of amphotericin B inhalation powder in a guinea pig model of invasive pulmonary aspergillosis. *J. Antimicrob. Chemother.* 67, 970–976. doi:10.1093/jac/dkr567.

Kirkpatrick, W. R., Perea, S., Coco, B. J., and Patterson, T. F. (2002a). Efficacy of caspofungin alone and in combination with voriconazole in a Guinea pig model of invasive aspergillosis. *Antimicrob. Agents Chemother.* 46, 2564–2568.

Kirkpatrick, W. R., Perea, S., Coco, B. J., and Patterson, T. F. (2002b). Efficacy of ravuconazole (BMS-207147) in a guinea pig model of disseminated aspergillosis. *J. Antimicrob. Chemother.* 49, 353–357.

Kolattukudy, P. E., Lee, J. D., Rogers, L. M., Zimmerman, P., Ceselski, S., Fox, B., et al. (1993). Evidence for possible involvement of an elastolytic serine protease in aspergillosis. *Infect. Immun.* 61, 2357–2368.

Kothary, M. H., Chase, T., and Macmillan, J. D. (1984). Correlation of elastase production by some strains of Aspergillus fumigatus with ability to cause pulmonary invasive aspergillosis in mice. *Infect. Immun.* 43, 320–325.

Kotz, A., Wagener, J., Engel, J., Routier, F., Echtenacher, B., Pich, A., et al. (2010). The mitA gene of Aspergillus fumigatus is required for mannosylation of inositol-phosphorylceramide, but is dispensable for pathogenicity. *Fungal Genet. Biol. FG B* 47, 169–178. doi:10.1016/j.fgb.2009.10.001.

Krappmann, S., Bignell, E. M., Reichard, U., Rogers, T., Haynes, K., and Braus, G. H. (2004). The Aspergillus fumigatus transcriptional activator CpcA contributes significantly to the virulence of this fungal pathogen. *Mol. Microbiol.* 52, 785–799. doi:10.1111/j.1365-2958.2004.04015.x.

Kretschmar, M., Buchheidt, D., Hof, H., and Nichterlein, T. (2001). Galactomannan enzyme immunoassay for monitoring systemic infection with Aspergillus fumigatus in mice. *Diagn. Microbiol. Infect. Dis.* 41, 107–112.

Krishnan, K., Feng, X., Powers-Fletcher, M. V., Bick, G., Richie, D. L., Woollett, L. A., et al. (2013). Effects of a defective endoplasmic reticulum-associated degradation pathway on the stress response, virulence, and antifungal drug susceptibility of the mold pathogen Aspergillus fumigatus. *Eukaryot. Cell* 12, 512–519. doi:10.1128/EC.00319-12.

Krishnan Natesan, S., Wu, W., Cutright, J. L., and Chandrasekar, P. H. (2012). In vitro-in vivo correlation of voriconazole resistance due to G448S mutation (cyp51A gene) in Aspergillus fumigatus. *Diagn. Microbiol. Infect. Dis.* 74, 272–277. doi:10.1016/j.diagmicrobio.2012.06.030.

Kumaresan, P. R., Manuri, P. R., Albert, N. D., Maiti, S., Singh, H., Mi, T., et al. (2014). Bioengineering T cells to target carbohydrate to treat opportunistic fungal infection. *Proc. Natl. Acad. Sci. U. S. A.* 111, 10660–10665. doi:10.1073/pnas.1312789111.

Kupfahl, C., Heinekamp, T., Geginat, G., Ruppert, T., Härtl, A., Hof, H., et al. (2006). Deletion of the gliP gene of Aspergillus fumigatus results in loss of gliotoxin production but has no effect on virulence of the fungus in a low-dose mouse infection model. *Mol. Microbiol.* 62, 292–302. doi:10.1111/j.1365-2958.2006.05373.x.

Kurtz, M. B., Bernard, E. M., Edwards, F. F., Marrinan, J. A., Dropinski, J., Douglas, C. M., et al. (1995). Aerosol and parenteral pneumocandins are effective in a rat model of pulmonary aspergillosis. *Antimicrob. Agents Chemother.* 39, 1784–1789.

Kwon-Chung, K. J. (1975). A new pathogenic species of Aspergillus in the Aspergillus fumigatus series. *Mycologia* 67, 770–779.

Lamarre, C., Ibrahim-Granet, O., Du, C., Calderone, R., and Latgé, J.-P. (2007). Characterization of the SKN7 ortholog of Aspergillus fumigatus. *Fungal Genet. Biol. FG B* 44, 682–690. doi:10.1016/j.fgb.2007.01.009.

Lambou, K., Lamarre, C., Beau, R., Dufour, N., and Latge, J.-P. (2010). Functional analysis of the superoxide dismutase family in Aspergillus fumigatus. *Mol. Microbiol.* 75, 910–923. doi:10.1111/j.1365-2958.2009.07024.x.

Lamoth, F., Juvvadi, P. R., Gehrke, C., Asfaw, Y. G., and Steinbach, W. J. (2014a). Transcriptional activation of heat shock protein 90 mediated via a proximal promoter region as trigger of caspofungin resistance in Aspergillus fumigatus. *J. Infect. Dis.* 209, 473–481. doi:10.1093/infdis/jit530.

Lamoth, F., Juvvadi, P. R., Soderblom, E. J., Moseley, M. A., Asfaw, Y. G., and Steinbach, W. J. (2014b). Identification of a key lysine residue in heat shock protein 90 required for azole and echinocandin resistance in Aspergillus fumigatus. *Antimicrob. Agents Chemother.* 58, 1889–1896. doi:10.1128/AAC.02286-13.

Land, C. J., Sostarić, B., Fuchs, R., Lundström, H., and Hult, K. (1989). Intratracheal exposure of rats to Aspergillus fumigatus spores isolated from sawmills in Sweden. *Appl. Environ. Microbiol.* 55, 2856–2860.

Langfelder, K., Jahn, B., Gehringer, H., Schmidt, A., Wanner, G., and Brakhage, A. A. (1998). Identification of a polyketide synthase gene (pksP) of Aspergillus fumigatus involved in conidial pigment biosynthesis and virulence. *Med. Microbiol. Immunol. (Berl.)* 187, 79–89.

Langfelder, K., Philippe, B., Jahn, B., Latgé, J. P., and Brakhage, A. A. (2001). Differential expression of the Aspergillus fumigatus pksP gene detected in vitro and in vivo with green fluorescent protein. *Infect. Immun.* 69, 6411–6418. doi:10.1128/IAI.69.10.6411-6418.2001.

Lass-Flörl, C., Speth, C., Mayr, A., Würzner, R., Dierich, M. P., Ulmer, H., et al. (2003). Diagnosing and monitoring of invasive aspergillosis during antifungal therapy by polymerase chain reaction: an experimental study in mice. *Diagn. Microbiol. Infect. Dis.* 47, 569–572.

Le Conte, P., Joly, V., Saint-Julien, L., Gillardin, J. M., Carbon, C., and Yeni, P. (1992). Tissue distribution and antifungal effect of liposomal itraconazole in experimental cryptococcosis and pulmonary aspergillosis. *Am. Rev. Respir. Dis.* 145, 424–429. doi:10.1164/ajrccm/145.2_Pt_1.424.

Leal, S. M., Cowden, S., Hsia, Y.-C., Ghannoum, M. A., Momany, M., and Pearlman, E. (2010). Distinct roles for Dectin-1 and TLR4 in the pathogenesis of Aspergillus fumigatus keratitis. *PLoS Pathog.* 6, e1000976. doi:10.1371/journal.ppat.1000976.

Leal, S. M., Roy, S., Vareechon, C., Carrion, S. deJesus, Clark, H., Lopez-Berges, M. S., et al. (2013). Targeting iron acquisition blocks infection with the fungal pathogens Aspergillus fumigatus and Fusarium oxysporum. *PLoS Pathog.* 9, e1003436. doi:10.1371/journal.ppat.1003436.

Leal, S. M., Vareechon, C., Cowden, S., Cobb, B. A., Latgé, J.-P., Momany, M., et al. (2012). Fungal antioxidant pathways promote survival against neutrophils during infection. *J. Clin. Invest.* 122, 2482–2498. doi:10.1172/JCI63239.

Lee, I., Oh, J.-H., Shwab, E. K., Dagenais, T. R. T., Andes, D., and Keller, N. P. (2009). HdaA, a class 2 histone deacetylase of Aspergillus fumigatus, affects germination and secondary metabolite production. *Fungal Genet. Biol. FG B* 46, 782–790. doi:10.1016/j.fgb.2009.06.007.

Lee, J. D., and Kolattukudy, P. E. (1995). Molecular cloning of the cDNA and gene for an elastinolytic aspartic proteinase from Aspergillus fumigatus and evidence of its secretion by the fungus during invasion of the host lung. *Infect. Immun.* 63, 3796–3803.

Leenders, A. C., de Marie, S., ten Kate, M. T., Bakker-Woudenberg, I. A., and Verbrugh, H. A. (1996). Liposomal amphotericin B (AmBisome) reduces dissemination of infection as compared with amphotericin B deoxycholate (Fungizone) in a rate model of pulmonary aspergillosis. *J. Antimicrob. Chemother.* 38, 215–225.

Lehmann, P. F., and White, L. O. (1975). Chitin assay used to demonstrate renal localization and cortisone-enhanced growth of Aspergillus fumigatus mycelium in mice. *Infect. Immun.* 12, 987–992.

Lehmann, P. F., and White, L. O. (1976). Acquired immunity to Aspergillus fumigatus. *Infect. Immun.* 13, 1296–1298.

Lehmann, P. F., and White, L. O. (1978). Rapid germination of Aspergillus fumigatus conidia in mouse kidneys and a kidney extract. *Sabouraudia* 16, 203–209.

Leleu, C., Menotti, J., Meneceur, P., Choukri, F., Sulahian, A., Garin, Y. J.-F., et al. (2013a). Bayesian development of a dose-response model for Aspergillus fumigatus and invasive aspergillosis. *Risk Anal. Off. Publ. Soc. Risk Anal.* 33, 1441–1453. doi:10.1111/risa.12007.

Leleu, C., Menotti, J., Meneceur, P., Choukri, F., Sulahian, A., Garin, Y. J.-F., et al. (2013b). Efficacy of liposomal amphotericin B for prophylaxis of acute or reactivation models of invasive pulmonary aspergillosis. *Mycoses* 56, 241–249. doi:10.1111/myc.12011.

Lengerova, M., Kocmanova, I., Racil, Z., Hrncirova, K., Pospisilova, S., Mayer, J., et al. (2012). Detection and measurement of fungal burden in a guinea pig model of invasive pulmonary aspergillosis by novel quantitative nested real-time PCR compared with galactomannan and (1,3)-β-D-glucan detection. *J. Clin. Microbiol.* 50, 602–608. doi:10.1128/JCM.05356-11.

Lepak, A. J., Marchillo, K., VanHecker, J., and Andes, D. R. (2013a). Impact of in vivo triazole and echinocandin combination therapy for invasive pulmonary aspergillosis: enhanced efficacy against Cyp51 mutant isolates. *Antimicrob. Agents Chemother.* 57, 5438–5447. doi:10.1128/AAC.00833-13.

Lepak, A. J., Marchillo, K., Vanhecker, J., and Andes, D. R. (2013b). Isavuconazole (BAL4815) pharmacodynamic target determination in an in vivo murine model of invasive pulmonary aspergillosis against wild-type and cyp51 mutant isolates of Aspergillus fumigatus. *Antimicrob. Agents Chemother.* 57, 6284–6289. doi:10.1128/AAC.01355-13.

Lepak, A. J., Marchillo, K., Vanhecker, J., and Andes, D. R. (2013c). Posaconazole pharmacodynamic target determination against wild-type and Cyp51 mutant isolates of Aspergillus fumigatus in an in vivo model of invasive pulmonary aspergillosis. *Antimicrob. Agents Chemother.* 57, 579–585. doi:10.1128/AAC.01279-12.

Lessing, F., Kniemeyer, O., Wozniok, I., Loeffler, J., Kurzai, O., Haertl, A., et al. (2007). The Aspergillus fumigatus transcriptional regulator AfYap1 represents the major regulator for defense against reactive oxygen intermediates but is dispensable for pathogenicity in an intranasal mouse infection model. *Eukaryot. Cell* 6, 2290–2302. doi:10.1128/EC.00267-07.

Levdansky, E., Kashi, O., Sharon, H., Shadkchan, Y., and Osherov, N. (2010). The Aspergillus fumigatus cspA gene encoding a repeat-rich cell wall protein is important for normal conidial cell wall architecture and interaction with host cells. *Eukaryot. Cell* 9, 1403–1415. doi:10.1128/EC.00126-10.

Lewis, R. E., Albert, N. D., and Kontoyiannis, D. P. (2008a). Comparison of the dose-dependent activity and paradoxical effect of caspofungin and micafungin in a neutropenic murine model of invasive pulmonary aspergillosis. *J. Antimicrob. Chemother.* 61, 1140–1144. doi:10.1093/jac/dkn069.

Lewis, R. E., Albert, N. D., and Kontoyiannis, D. P. (2008b). Efficacy of single-dose liposomal amphotericin B or micafungin prophylaxis in a neutropenic murine model of invasive pulmonary aspergillosis. *Antimicrob. Agents Chemother.* 52, 4178–4180. doi:10.1128/AAC.00715-08.

Lewis, R. E., Albert, N. D., and Kontoyiannis, D. P. (2014). Comparative pharmacodynamics of posaconazole in neutropenic murine models of invasive pulmonary aspergillosis and mucormycosis. *Antimicrob. Agents Chemother.* 58, 6767–6772. doi:10.1128/AAC.03569-14.

Lewis, R. E., Liao, G., Hou, J., Chamilos, G., Prince, R. A., and Kontoyiannis, D. P. (2007). Comparative analysis of amphotericin B lipid complex and liposomal amphotericin B kinetics of lung accumulation and fungal clearance in a murine model of acute invasive pulmonary aspergillosis. *Antimicrob. Agents Chemother.* 51, 1253–1258. doi:10.1128/AAC.01449-06.

Lewis, R. E., Liao, G., Hou, J., Prince, R. A., and Kontoyiannis, D. P. (2011a). Comparative in vivo dose-dependent activity of caspofungin and anidulafungin against echinocandin-susceptible and -resistant Aspergillus fumigatus. *J. Antimicrob. Chemother.* 66, 1324–1331. doi:10.1093/jac/dkr142.

Lewis, R. E., Liao, G., Wang, W., Prince, R. A., and Kontoyiannis, D. P. (2011b). Voriconazole pre-exposure selects for breakthrough mucormycosis in a mixed model of Aspergillus fumigatus-Rhizopus oryzae pulmonary infection. *Virulence* 2, 348–355.

Lewis, R. E., Prince, R. A., Chi, J., and Kontoyiannis, D. P. (2002). Itraconazole preexposure attenuates the efficacy of subsequent amphotericin B therapy in a murine model of acute invasive pulmonary aspergillosis. *Antimicrob. Agents Chemother.* 46, 3208–3214.

Lewis, R. E., Wiederhold, N. P., Chi, J., Han, X. Y., Komanduri, K. V., Kontoyiannis, D. P., et al. (2005). Detection of gliotoxin in experimental and human aspergillosis. *Infect. Immun.* 73, 635–637. doi:10.1128/IAI.73.1.635-637.2005.

Li, C., Zhao, G., Che, C., Lin, J., Li, N., Hu, L., et al. (2015a). The Role of LOX-1 in Innate Immunity to Aspergillus fumigatus in Corneal Epithelial Cells. *Invest. Ophthalmol. Vis. Sci.* 56, 3593–3603. doi:10.1167/iovs.14-15989.

Li, H., Barker, B. M., Grahl, N., Puttikamonkul, S., Bell, J. D., Craven, K. D., et al. (2011). The small GTPase RacA mediates intracellular reactive oxygen species production, polarized growth, and virulence in the human fungal pathogen Aspergillus fumigatus. *Eukaryot. Cell* 10, 174–186. doi:10.1128/EC.00288-10.

Li, H., Zhou, H., Luo, Y., Ouyang, H., Hu, H., and Jin, C. (2007). Glycosylphosphatidylinositol (GPI) anchor is required in Aspergillus fumigatus for morphogenesis and virulence. *Mol. Microbiol.* 64, 1014–1027. doi:10.1111/j.1365-2958.2007.05709.x.

Li, P., Xu, X., Cao, E., Yu, B., Li, W., Fan, M., et al. (2014). Vitamin D deficiency causes defective resistance to Aspergillus fumigatus in mice via aggravated and sustained inflammation. *PloS One* 9, e99805. doi:10.1371/journal.pone.0099805.

Li, S.-X., Song, Y.-J., Zhang, L.-L., Shi, J.-P., Ma, Z.-L., Guo, H., et al. (2015b). An in vitro and in vivo study on the synergistic effect and mechanism of itraconazole or voriconazole alone and in combination with tetrandrine against Aspergillus fumigatus. *J. Med. Microbiol.* 64, 1008–1020. doi:10.1099/jmm.0.000120.

Li, X., Gao, M., Han, X., Tao, S., Zheng, D., Cheng, Y., et al. (2012). Disruption of the phospholipase D gene attenuates the virulence of Aspergillus fumigatus. *Infect. Immun.* 80, 429–440. doi:10.1128/IAI.05830-11.

Li, Y., Zhang, L., Wang, D., Zhou, H., Ouyang, H., Ming, J., et al. (2008). Deletion of the msdS/AfmsdC gene induces abnormal polarity and septation in Aspergillus fumigatus. *Microbiol. Read. Engl.* 154, 1960–1972. doi:10.1099/mic.0.2008/017525-0.

Liebmann, B., Mühleisen, T. W., Müller, M., Hecht, M., Weidner, G., Braun, A., et al. (2004a). Deletion of the Aspergillus fumigatus lysine biosynthesis gene lysF encoding homoaconitase leads to attenuated virulence in a low-dose mouse infection model of invasive aspergillosis. *Arch. Microbiol.* 181, 378–383. doi:10.1007/s00203-004-0667-3.

Liebmann, B., Müller, M., Braun, A., and Brakhage, A. A. (2004b). The cyclic AMP-dependent protein kinase a network regulates development and virulence in Aspergillus fumigatus. *Infect. Immun.* 72, 5193–5203. doi:10.1128/IAI.72.9.5193-5203.2004.

Lilly, L. M., Scopel, M., Nelson, M. P., Burg, A. R., Dunaway, C. W., and Steele, C. (2014). Eosinophil deficiency compromises lung defense against Aspergillus fumigatus. *Infect. Immun.* 82, 1315–1325. doi:10.1128/IAI.01172-13.

Lin, J.-C., Xing, Y.-L., Xu, W.-M., Li, M., Bo, P., Niu, Y.-Y., et al. (2014). Evaluation of galactomannan enzyme immunoassay and quantitative real-time PCR for the diagnosis of invasive pulmonary aspergillosis in a rat model. *J. Microbiol. Biotechnol.* 24, 1044–1050.

Lin, L., Ibrahim, A. S., Baquir, B., Fu, Y., Applebaum, D., Schwartz, J., et al. (2010). Safety and efficacy of activated transfected killer cells for neutropenic fungal infections. *J. Infect. Dis.* 201, 1708–1717. doi:10.1086/652496.

Lin, L., Ibrahim, A. S., Baquir, B., Palosaari, A., and Spellberg, B. (2012). Luminescent-activated transfected killer cells to monitor leukocyte trafficking during systemic bacterial and fungal infection. *J. Infect. Dis.* 205, 337–347. doi:10.1093/infdis/jir725.

Lionakis, M. S., Lahdenranta, J., Sun, J., Liu, W., Lewis, R. E., Albert, N. D., et al. (2005). Development of a ligand-directed approach to study the pathogenesis of invasive aspergillosis. *Infect. Immun.* 73, 7747–7758. doi:10.1128/IAI.73.11.7747-7758.2005.

Liu, H., Gravelat, F. N., Chiang, L. Y., Chen, D., Vanier, G., Ejzykowicz, D. E., et al. (2010). Aspergillus fumigatus AcuM regulates both iron acquisition and gluconeogenesis. *Mol. Microbiol.* 78, 1038–1054. doi:10.1111/j.1365-2958.2010.07389.x.

Liu, M., Capilla, J., Johansen, M. E., Alvarado, D., Martinez, M., Chen, V., et al. (2011). Saccharomyces as a vaccine against systemic aspergillosis: “the friend of man” a friend again? *J. Med. Microbiol.* 60, 1423–1432. doi:10.1099/jmm.0.033290-0.

Liu, M., Machová, E., Neščáková, Z., Medovarská, I., Clemons, K. V., Martinez, M., et al. (2012). Vaccination with mannan protects mice against systemic aspergillosis. *Med. Mycol.* 50, 818–828. doi:10.3109/13693786.2012.683539.

Liu, W., Yan, M., Sugui, J. A., Li, H., Xu, C., Joo, J., et al. (2013). Olfm4 deletion enhances defense against Staphylococcus aureus in chronic granulomatous disease. *J. Clin. Invest.* 123, 3751–3755. doi:10.1172/JCI68453.

Lo Giudice, P., Campo, S., De Santis, R., and Salvatori, G. (2012). Effect of PTX3 and voriconazole combination in a rat model of invasive pulmonary aspergillosis. *Antimicrob. Agents Chemother.* 56, 6400–6402. doi:10.1128/AAC.01000-12.

Lo Giudice, P., Campo, S., Verdoliva, A., Rivieccio, V., Borsini, F., De Santis, R., et al. (2010). Efficacy of PTX3 in a rat model of invasive aspergillosis. *Antimicrob. Agents Chemother.* 54, 4513–4515. doi:10.1128/AAC.00674-10.

Loeffler, J., Kloepfer, K., Hebart, H., Najvar, L., Graybill, J. R., Kirkpatrick, W. R., et al. (2002). Polymerase chain reaction detection of aspergillus DNA in experimental models of invasive aspergillosis. *J. Infect. Dis.* 185, 1203–1206. doi:10.1086/339824.

Lohan, S., Monga, J., Chauhan, C. S., and Bisht, G. S. (2015). In Vitro and In Vivo Evaluation of Small Cationic Abiotic Lipopeptides as Novel Antifungal Agents. *Chem. Biol. Drug Des.* 86, 829–836. doi:10.1111/cbdd.12558.

Losada, L., Sugui, J. A., Eckhaus, M. A., Chang, Y. C., Mounaud, S., Figat, A., et al. (2015). Genetic Analysis Using an Isogenic Mating Pair of Aspergillus fumigatus Identifies Azole Resistance Genes and Lack of MAT Locus’s Role in Virulence. *PLoS Pathog.* 11, e1004834. doi:10.1371/journal.ppat.1004834.

Loussert, C., Schmitt, C., Prevost, M.-C., Balloy, V., Fadel, E., Philippe, B., et al. (2010). In vivo biofilm composition of Aspergillus fumigatus. *Cell. Microbiol.* 12, 405–410. doi:10.1111/j.1462-5822.2009.01409.x.

Lupetti, A., Welling, M. M., Mazzi, U., Nibbering, P. H., and Pauwels, E. K. J. (2002). Technetium-99m labelled fluconazole and antimicrobial peptides for imaging of Candida albicans and Aspergillus fumigatus infections. *Eur. J. Nucl. Med. Mol. Imaging* 29, 674–679. doi:10.1007/s00259-001-0760-7.

Luque, J. C., Clemons, K. V., and Stevens, D. A. (2003). Efficacy of micafungin alone or in combination against systemic murine aspergillosis. *Antimicrob. Agents Chemother.* 47, 1452–1455.

Ma, Y., Qiao, J., Liu, W., Wan, Z., Wang, X., Calderone, R., et al. (2008). The sho1 sensor regulates growth, morphology, and oxidant adaptation in Aspergillus fumigatus but is not essential for development of invasive pulmonary aspergillosis. *Infect. Immun.* 76, 1695–1701. doi:10.1128/IAI.01507-07.

MacCallum, D. M., Whyte, J. A., and Odds, F. C. (2005). Efficacy of caspofungin and voriconazole combinations in experimental aspergillosis. *Antimicrob. Agents Chemother.* 49, 3697–3701. doi:10.1128/AAC.49.9.3697-3701.2005.

Macheleidt, J., Scherlach, K., Neuwirth, T., Schmidt-Heck, W., Straßburger, M., Spraker, J., et al. (2015). Transcriptome analysis of cyclic AMP-dependent protein kinase A-regulated genes reveals the production of the novel natural compound fumipyrrole by Aspergillus fumigatus. *Mol. Microbiol.* 96, 148–162. doi:10.1111/mmi.12926.

Madan, T., Kishore, U., Singh, M., Strong, P., Clark, H., Hussain, E. M., et al. (2001). Surfactant proteins A and D protect mice against pulmonary hypersensitivity induced by Aspergillus fumigatus antigens and allergens. *J. Clin. Invest.* 107, 467–475. doi:10.1172/JCI10124.

Madan, T., Reid, K. B. M., Clark, H., Singh, M., Nayak, A., Sarma, P. U., et al. (2010). Susceptibility of mice genetically deficient in SP-A or SP-D gene to invasive pulmonary aspergillosis. *Mol. Immunol.* 47, 1923–1930. doi:10.1016/j.molimm.2010.02.027.

Maheshwari, R. K., Tandon, R. N., Feuillette, A. R., Mahouy, G., Badillet, G., and Friedman, R. M. (1988). Interferon inhibits Aspergillus fumigatus growth in mice: an activity against an extracellular infection. *J. Interferon Res.* 8, 35–44.

Manavathu, E. K., Cutright, J. L., and Chandrasekar, P. H. (2005). In vivo resistance of a laboratory-selected Aspergillus fumigatus isolate to amphotericin B. *Antimicrob. Agents Chemother.* 49, 428–430. doi:10.1128/AAC.49.1.428-430.2005.

Manavathu, E. K., Dimmock, J. R., Vashishtha, S. C., Cutright, J., and Chandrasekar, P. H. (1998). In-vitro and in-vivo susceptibility of Aspergillus fumigatus to a novel conjugated styryl ketone. *J. Antimicrob. Chemother.* 42, 585–590.

Markaryan, A., Morozova, I., Yu, H., and Kolattukudy, P. E. (1994). Purification and characterization of an elastinolytic metalloprotease from Aspergillus fumigatus and immunoelectron microscopic evidence of secretion of this enzyme by the fungus invading the murine lung. *Infect. Immun.* 62, 2149–2157.

Marples, B., Downing, L., Sawarynski, K. E., Finkelstein, J. N., Williams, J. P., Martinez, A. A., et al. (2011). Pulmonary injury after combined exposures to low-dose low-LET radiation and fungal spores. *Radiat. Res.* 175, 501–509. doi:10.1667/RR2379.1.

Marra, E., Sousa, V. L., Gaziano, R., Pacello, M. L., Arseni, B., Aurisicchio, L., et al. (2014). Efficacy of PTX3 and posaconazole combination in a rat model of invasive pulmonary aspergillosis. *Antimicrob. Agents Chemother.* 58, 6284–6286. doi:10.1128/AAC.03038-14.

Martín, M. T., Gavaldà, J., López, P., Gomis, X., Ramírez, J. L., Rodríguez, D., et al. (2003). Efficacy of high doses of liposomal amphotericin B in the treatment of experimental aspergillosis. *J. Antimicrob. Chemother.* 52, 1032–1034. doi:10.1093/jac/dkh003.

Martin, M. V., Yates, J., and Hitchcock, C. A. (1997). Comparison of voriconazole (UK-109,496) and itraconazole in prevention and treatment of Aspergillus fumigatus endocarditis in guinea pigs. *Antimicrob. Agents Chemother.* 41, 13–16.

Martinez, A., Aviles, P., Jimenez, E., Caballero, J., and Gargallo-Viola, D. (2000). Activities of sordarins in experimental models of candidiasis, aspergillosis, and pneumocystosis. *Antimicrob. Agents Chemother.* 44, 3389–3394.

Martinez, J., Malireddi, R. K. S., Lu, Q., Cunha, L. D., Pelletier, S., Gingras, S., et al. (2015). Molecular characterization of LC3-associated phagocytosis reveals distinct roles for Rubicon, NOX2 and autophagy proteins. *Nat. Cell Biol.* 17, 893–906. doi:10.1038/ncb3192.

Martinez, M., Chen, V., Tong, A.-J., Hamilton, K., Clemons, K. V., and Stevens, D. A. (2013). Experimental evidence that granulocyte transfusions are efficacious in treatment of neutropenic hosts with pulmonary aspergillosis. *Antimicrob. Agents Chemother.* 57, 1882–1887. doi:10.1128/AAC.02533-12.

Matsumoto, S., Wakai, Y., Nakai, T., Hatano, K., Ushitani, T., Ikeda, F., et al. (2000). Efficacy of FK463, a new lipopeptide antifungal agent, in mouse models of pulmonary aspergillosis. *Antimicrob. Agents Chemother.* 44, 619–621.

Mattila, P. E., Metz, A. E., Rapaka, R. R., Bauer, L. D., and Steele, C. (2008). Dectin-1 Fc targeting of aspergillus fumigatus beta-glucans augments innate defense against invasive pulmonary aspergillosis. *Antimicrob. Agents Chemother.* 52, 1171–1172. doi:10.1128/AAC.01274-07.

Maubon, D., Park, S., Tanguy, M., Huerre, M., Schmitt, C., Prévost, M. C., et al. (2006). AGS3, an alpha(1-3)glucan synthase gene family member of Aspergillus fumigatus, modulates mycelium growth in the lung of experimentally infected mice. *Fungal Genet. Biol. FG B* 43, 366–375. doi:10.1016/j.fgb.2006.01.006.

Mavridou, E., Brüggemann, R. J. M., Melchers, W. J. G., Mouton, J. W., and Verweij, P. E. (2010a). Efficacy of posaconazole against three clinical Aspergillus fumigatus isolates with mutations in the cyp51A gene. *Antimicrob. Agents Chemother.* 54, 860–865. doi:10.1128/AAC.00931-09.

Mavridou, E., Bruggemann, R. J. M., Melchers, W. J. G., Verweij, P. E., and Mouton, J. W. (2010b). Impact of cyp51A mutations on the pharmacokinetic and pharmacodynamic properties of voriconazole in a murine model of disseminated aspergillosis. *Antimicrob. Agents Chemother.* 54, 4758–4764. doi:10.1128/AAC.00606-10.

Mazaki, Y., Hashimoto, S., Tsujimura, T., Morishige, M., Hashimoto, A., Aritake, K., et al. (2006). Neutrophil direction sensing and superoxide production linked by the GTPase-activating protein GIT2. *Nat. Immunol.* 7, 724–731. doi:10.1038/ni1349.

Mazzolla, R., Barluzzi, R., Romani, L., Mosci, P., and Bistoni, F. (1991). Anti-Candida resistance in the mouse brain and effect of intracerebral administration of interleukin 1. *J. Gen. Microbiol.* 137, 1799–1804. doi:10.1099/00221287-137-8-1799.

McCormick, A., Jacobsen, I. D., Broniszewska, M., Beck, J., Heesemann, J., and Ebel, F. (2012). The two-component sensor kinase TcsC and its role in stress resistance of the human-pathogenic mold Aspergillus fumigatus. *PloS One* 7, e38262. doi:10.1371/journal.pone.0038262.

McCulloch, E., Ramage, G., Jones, B., Warn, P., Kirkpatrick, W. R., Patterson, T. F., et al. (2009). Don’t throw your blood clots away: use of blood clot may improve sensitivity of PCR diagnosis in invasive aspergillosis. *J. Clin. Pathol.* 62, 539–541. doi:10.1136/jcp.2008.063321.

McCulloch, E., Ramage, G., Rajendran, R., Lappin, D. F., Jones, B., Warn, P., et al. (2012). Antifungal treatment affects the laboratory diagnosis of invasive aspergillosis. *J. Clin. Pathol.* 65, 83–86. doi:10.1136/jcp.2011.090464.

McDonagh, A., Fedorova, N. D., Crabtree, J., Yu, Y., Kim, S., Chen, D., et al. (2008). Sub-telomere directed gene expression during initiation of invasive aspergillosis. *PLoS Pathog.* 4, e1000154. doi:10.1371/journal.ppat.1000154.

Mehrad, B., Moore, T. A., and Standiford, T. J. (2000). Macrophage inflammatory protein-1 alpha is a critical mediator of host defense against invasive pulmonary aspergillosis in neutropenic hosts. *J. Immunol. Baltim. Md 1950* 165, 962–968.

Mehrad, B., Strieter, R. M., Moore, T. A., Tsai, W. C., Lira, S. A., and Standiford, T. J. (1999a). CXC chemokine receptor-2 ligands are necessary components of neutrophil-mediated host defense in invasive pulmonary aspergillosis. *J. Immunol. Baltim. Md 1950* 163, 6086–6094.

Mehrad, B., Strieter, R. M., and Standiford, T. J. (1999b). Role of TNF-alpha in pulmonary host defense in murine invasive aspergillosis. *J. Immunol. Baltim. Md 1950* 162, 1633–1640.

Mehrad, B., Wiekowski, M., Morrison, B. E., Chen, S.-C., Coronel, E. C., Manfra, D. J., et al. (2002). Transient lung-specific expression of the chemokine KC improves outcome in invasive aspergillosis. *Am. J. Respir. Crit. Care Med.* 166, 1263–1268. doi:10.1164/rccm.200204-367OC.

Melchers, W. J., Verweij, P. E., van den Hurk, P., van Belkum, A., De Pauw, B. E., Hoogkamp-Korstanje, J. A., et al. (1994). General primer-mediated PCR for detection of Aspergillus species. *J. Clin. Microbiol.* 32, 1710–1717.

Mellado, E., Alcazar-Fuoli, L., Cuenca-Estrella, M., and Rodriguez-Tudela, J. L. (2011). Role of Aspergillus lentulus 14-α sterol demethylase (Cyp51A) in azole drug susceptibility. *Antimicrob. Agents Chemother.* 55, 5459–5468. doi:10.1128/AAC.05178-11.

Mellado, E., Aufauvre-Brown, A., Gow, N. A., and Holden, D. W. (1996). The Aspergillus fumigatus chsC and chsG genes encode class III chitin synthases with different functions. *Mol. Microbiol.* 20, 667–679.

Mellado, E., Garcia-Effron, G., Buitrago, M. J., Alcazar-Fuoli, L., Cuenca-Estrella, M., and Rodriguez-Tudela, J. L. (2005). Targeted gene disruption of the 14-alpha sterol demethylase (cyp51A) in Aspergillus fumigatus and its role in azole drug susceptibility. *Antimicrob. Agents Chemother.* 49, 2536–2538. doi:10.1128/AAC.49.6.2536-2538.2005.

Mircescu, M. M., Lipuma, L., van Rooijen, N., Pamer, E. G., and Hohl, T. M. (2009). Essential role for neutrophils but not alveolar macrophages at early time points following Aspergillus fumigatus infection. *J. Infect. Dis.* 200, 647–656. doi:10.1086/600380.

Mirkov, I., Belij, S., Kataranovski, M., Zolotarevski, L., Glamoclija, J., Stojanovic, I., et al. (2012). The relevance of the migration inhibitory factor (MIF) for peripheral tissue response in murine sublethal systemic Aspergillus fumigatus infection. *Med. Mycol.* 50, 476–487. doi:10.3109/13693786.2011.645893.

Mirkov, I., Demenesku, J., Popov Aleksandrov, A., Ninkov, M., Glamoclija, J., Kataranovski, D., et al. (2015). Strain differences in the immune mechanisms of resistance of immunocompetent rats to pulmonary aspergillosis. *Immunobiology* 220, 1075–1084. doi:10.1016/j.imbio.2015.05.007.

Mirkov, I., El-Muzghi, A. A. M., Djokic, J., Ninkov, M., Popov Aleksandrov, A., Glamoclija, J., et al. (2014). Pulmonary immune responses to Aspergillus fumigatus in rats. *Biomed. Environ. Sci. BES* 27, 684–694. doi:10.3967/bes2014.104.

Mirkov, I., Glamoclija, J., Stosic-Grujicic, S., Zolotarevski, L., Kataranovski, D., and Kataranovski, M. (2013). Differential strain-related tissue immune response to sublethal systemic Aspergillus fumigatus infection in mice. *APMIS Acta Pathol. Microbiol. Immunol. Scand.* 121, 211–220. doi:10.1111/j.1600-0463.2012.02958.x.

Mirkov, I., Stojanovic, I., Glamoclija, J., Stosic-Grujicic, S., Zolotarevski, L., Kataranovski, D., et al. (2011). Differential mechanisms of resistance to sublethal systemic Aspergillus fumigatus infection in immunocompetent BALB/c and C57BL/6 mice. *Immunobiology* 216, 234–242. doi:10.1016/j.imbio.2010.04.007.

Mirkov, I., Stojanovic, I., Stosic-Grujicic, S., Glamoclija, J., Zolotarevski, L., Kataranovski, D., et al. (2010). Splenic and lung response to nonlethal systemic Aspergillus fumigatus infection in C57BL/6 mice. *Med. Mycol.* 48, 735–743. doi:10.3109/13693780903496591.

Mitsutake, K., Kohno, S., Miyazaki, T., Yamamoto, Y., Yanagihara, K., Kakeya, H., et al. (1995). Detection of (1-3)-beta-D-glucan in a rat model of aspergillosis. *J. Clin. Lab. Anal.* 9, 119–122.

Mitsuyama, J., Kizawa, K., Minami, S., Watanabe, Y., and Yamaguchi, K. (2003). Evaluation of antimicrobial agents using an experimental pulmonary superinfection model with Aspergillus fumigatus and Pseudomonas aeruginosain leukopenic mice. *J. Infect. Chemother. Off. J. Jpn. Soc. Chemother.* 9, 144–150. doi:10.1007/s10156-003-0234-x.

Mitsuyama, J., Nomura, N., Hashimoto, K., Yamada, E., Nishikawa, H., Kaeriyama, M., et al. (2008). In vitro and in vivo antifungal activities of T-2307, a novel arylamidine. *Antimicrob. Agents Chemother.* 52, 1318–1324. doi:10.1128/AAC.01159-07.

Miyazaki, H. M., Kohno, S., Miyazaki, Y., Mitsutake, K., Tomono, K., Kaku, M., et al. (1993). Efficacy of intravenous itraconazole against experimental pulmonary aspergillosis. *Antimicrob. Agents Chemother.* 37, 2762–2765.

Moalli, F., Doni, A., Deban, L., Zelante, T., Zagarella, S., Bottazzi, B., et al. (2010). Role of complement and Fc{gamma} receptors in the protective activity of the long pentraxin PTX3 against Aspergillus fumigatus. *Blood* 116, 5170–5180. doi:10.1182/blood-2009-12-258376.

Mondon, P., De Champs, C., Donadille, A., Ambroise-Thomas, P., and Grillot, R. (1996). Variation in virulence of Aspergillus fumigatus strains in a murine model of invasive pulmonary aspergillosis. *J. Med. Microbiol.* 45, 186–191. doi:10.1099/00222615-45-3-186.

Monga, D. P. (1983). Studies on experimental aspergillosis in immunodeficient mice. *Zentralblatt Für Bakteriol. Mikrobiol. Hyg. 1 Abt Orig. Med. Mikrobiol. Infekt. Parasitol. Int. J. Microbiol. Hyg. Med. Microbiol. Infect. Parasitol.* 254, 552–560.

Monod, M., Paris, S., Sarfati, J., Jaton-Ogay, K., Ave, P., and Latgé, J. P. (1993). Virulence of alkaline protease-deficient mutants of Aspergillus fumigatus. *FEMS Microbiol. Lett.* 106, 39–46.

Monroy, F., and Sheppard, D. C. (2005). Taf1: a class II transposon of Aspergillus fumigatus. *Fungal Genet. Biol. FG B* 42, 638–645. doi:10.1016/j.fgb.2005.04.003.

Montagnoli, C., Bozza, S., Bacci, A., Gaziano, R., Mosci, P., Morschhäuser, J., et al. (2003). A role for antibodies in the generation of memory antifungal immunity. *Eur. J. Immunol.* 33, 1193–1204. doi:10.1002/eji.200323790.

Montagnoli, C., Fallarino, F., Gaziano, R., Bozza, S., Bellocchio, S., Zelante, T., et al. (2006). Immunity and tolerance to Aspergillus involve functionally distinct regulatory T cells and tryptophan catabolism. *J. Immunol. Baltim. Md 1950* 176, 1712–1723.

Moonis, M., Ahmad, I., and Bachhawat, B. K. (1992). Liposomal hamycin in the control of experimental aspergillosis in mice: relative toxicity, therapeutic efficacy and tissue distribution of free and liposomal hamycin. *Indian J. Biochem. Biophys.* 29, 339–345.

Moonis, M., Ahmad, I., and Bachhawat, B. K. (1993a). Liposomal hamycin in the control of experimental aspergillosis in mice: effect of phosphatidic acid with and without cholesterol. *J. Antimicrob. Chemother.* 31, 569–579.

Moonis, M., Ahmad, I., and Bachhawat, B. K. (1993b). Mannosylated liposomes as carriers for hamycin in the treatment of experimental aspergillosis in Balb/C mice. *J. Drug Target.* 1, 147–155. doi:10.3109/10611869308996071.

Moonis, M., Ahmad, I., and Bachhawat, B. K. (1994). Effect of elimination of phagocytic cells by liposomal dichloromethylene diphosphonate on aspergillosis virulence and toxicity of liposomal amphotericin B in mice. *J. Antimicrob. Chemother.* 33, 571–583.

Moretti, S., Bellocchio, S., Bonifazi, P., Bozza, S., Zelante, T., Bistoni, F., et al. (2008). The contribution of PARs to inflammation and immunity to fungi. *Mucosal Immunol.* 1, 156–168. doi:10.1038/mi.2007.13.

Moretti, S., Bozza, S., D’Angelo, C., Casagrande, A., Della Fazia, M. A., Pitzurra, L., et al. (2012). Role of innate immune receptors in paradoxical caspofungin activity in vivo in preclinical aspergillosis. *Antimicrob. Agents Chemother.* 56, 4268–4276. doi:10.1128/AAC.05198-11.

Moretti, S., Bozza, S., Massi-Benedetti, C., Prezioso, L., Rossetti, E., Romani, L., et al. (2014a). An immunomodulatory activity of micafungin in preclinical aspergillosis. *J. Antimicrob. Chemother.* 69, 1065–1074. doi:10.1093/jac/dkt457.

Moretti, S., Bozza, S., Oikonomou, V., Renga, G., Casagrande, A., Iannitti, R. G., et al. (2014b). IL-37 inhibits inflammasome activation and disease severity in murine aspergillosis. *PLoS Pathog.* 10, e1004462. doi:10.1371/journal.ppat.1004462.

Morgenstern, D. E., Gifford, M. A., Li, L. L., Doerschuk, C. M., and Dinauer, M. C. (1997). Absence of respiratory burst in X-linked chronic granulomatous disease mice leads to abnormalities in both host defense and inflammatory response to Aspergillus fumigatus. *J. Exp. Med.* 185, 207–218.

Morisse, H., Heyman, L., Salaün, M., Favennec, L., Picquenot, J. M., Bohn, P., et al. (2012). In vivo and in situ imaging of experimental invasive pulmonary aspergillosis using fibered confocal fluorescence microscopy. *Med. Mycol.* 50, 386–395. doi:10.3109/13693786.2011.617788.

Morisse, H., Heyman, L., Salaün, M., Favennec, L., Picquenot, J. M., Bohn, P., et al. (2013). In vivo molecular microimaging of pulmonary aspergillosis. *Med. Mycol.* 51, 352–360. doi:10.3109/13693786.2012.729138.

Morrison, B. E., Park, S. J., Mooney, J. M., and Mehrad, B. (2003). Chemokine-mediated recruitment of NK cells is a critical host defense mechanism in invasive aspergillosis. *J. Clin. Invest.* 112, 1862–1870. doi:10.1172/JCI200318125.

Morton, C. O., Clemons, K. V., Springer, J., Mueller, J. G., Rogers, T. R., Stevens, D. A., et al. (2011). Real-time PCR and quantitative culture for monitoring of experimental Aspergillus fumigatus intracranial infection in neutropenic mice. *J. Med. Microbiol.* 60, 913–919. doi:10.1099/jmm.0.028399-0.

Morton, C. O., de Luca, A., Romani, L., and Rogers, T. R. (2012). RT-qPCR detection of Aspergillus fumigatus RNA in vitro and in a murine model of invasive aspergillosis utilizing the PAXgene® and Tempus^TM^ RNA stabilization systems. *Med. Mycol.* 50, 661–666. doi:10.3109/13693786.2011.652200.

Morton, C. O., Loeffler, J., De Luca, A., Frost, S., Kenny, C., Duval, S., et al. (2010). Dynamics of extracellular release of Aspergillus fumigatus DNA and galactomannan during growth in blood and serum. *J. Med. Microbiol.* 59, 408–413. doi:10.1099/jmm.0.017418-0.

Mota Júnior, A. O., Malavazi, I., Soriani, F. M., Heinekamp, T., Jacobsen, I., Brakhage, A. A., et al. (2008). Molecular characterization of the Aspergillus fumigatus NCS-1 homologue, NcsA. *Mol. Genet. Genomics MGG* 280, 483–495. doi:10.1007/s00438-008-0381-y.

Moutaouakil, M., Monod, M., Prévost, M. C., Bouchara, J. P., Paris, S., and Latgé, J. P. (1993). Identification of the 33-kDa alkaline protease of Aspergillus fumigatus in vitro and in vivo. *J. Med. Microbiol.* 39, 393–399. doi:10.1099/00222615-39-5-393.

Mouton, J. W., te Dorsthorst, D. T. A., Meis, J. F. G. M., and Verweij, P. E. (2009). Dose-response relationships of three amphotericin B formulations in a non-neutropenic murine model of invasive aspergillosis. *Med. Mycol.* 47, 802–807. doi:10.3109/13693780802672644.

Mouyna, I., Kniemeyer, O., Jank, T., Loussert, C., Mellado, E., Aimanianda, V., et al. (2010). Members of protein O-mannosyltransferase family in Aspergillus fumigatus differentially affect growth, morphogenesis and viability. *Mol. Microbiol.* 76, 1205–1221. doi:10.1111/j.1365-2958.2010.07164.x.

Murphy, M., Bernard, E. M., Ishimaru, T., and Armstrong, D. (1997). Activity of voriconazole (UK-109,496) against clinical isolates of Aspergillus species and its effectiveness in an experimental model of invasive pulmonary aspergillosis. *Antimicrob. Agents Chemother.* 41, 696–698.

Muszkieta, L., Aimanianda, V., Mellado, E., Gribaldo, S., Alcàzar-Fuoli, L., Szewczyk, E., et al. (2014a). Deciphering the role of the chitin synthase families 1 and 2 in the in vivo and in vitro growth of Aspergillus fumigatus by multiple gene targeting deletion. *Cell. Microbiol.* 16, 1784–1805. doi:10.1111/cmi.12326.

Muszkieta, L., Carrion, S. de J., Robinet, P., Beau, R., Elbim, C., Pearlman, E., et al. (2014b). The protein phosphatase PhzA of A. fumigatus is involved in oxidative stress tolerance and fungal virulence. *Fungal Genet. Biol. FG B* 66, 79–85. doi:10.1016/j.fgb.2014.02.009.

Nagai, H., Guo, J., Choi, H., and Kurup, V. (1995). Interferon-gamma and tumor necrosis factor-alpha protect mice from invasive aspergillosis. *J. Infect. Dis.* 172, 1554–1560.

Nagasaki, Y., Eriguchi, Y., Uchida, Y., Miyake, N., Maehara, Y., Kadowaki, M., et al. (2009). Combination therapy with micafungin and amphotericin B for invasive pulmonary aspergillosis in an immunocompromised mouse model. *J. Antimicrob. Chemother.* 64, 379–382. doi:10.1093/jac/dkp175.

Naik, S. R., Thakare, V. N., Desai, S. K., and Rahalkar, P. R. (2011). Study of immunological aspects of aspergillosis in mice and effect of polyene macrolide antibiotic (SJA-95) and IFN-γ: a possible role of IFN-γ as an adjunct in antifungal therapy. *Immunol. Lett.* 141, 68–73. doi:10.1016/j.imlet.2011.07.008.

Najvar, L. K., Luther, M. F., and Graybill, J. R. (1995). Treatment of experimental systemic mycoses with BRL 49594A. *J. Antimicrob. Chemother.* 36, 1005–1011.

Nawada, R., Amitani, R., Tanaka, E., Niimi, A., Suzuki, K., Murayama, T., et al. (1996). Murine model of invasive pulmonary aspergillosis following an earlier stage, noninvasive Aspergillus infection. *J. Clin. Microbiol.* 34, 1433–1439.

Niki, Y., Bernard, E. M., Edwards, F. F., Schmitt, H. J., Yu, B., and Armstrong, D. (1991). Model of recurrent pulmonary aspergillosis in rats. *J. Clin. Microbiol.* 29, 1317–1322.

Niwano, Y., Kuzuhara, N., Goto, Y., Munechika, Y., Kodama, H., Kanai, K., et al. (1999). Efficacy of NND-502, a novel imidazole antimycotic agent, in experimental models of Candida albicans and Aspergillus fumigatus infections. *Int. J. Antimicrob. Agents* 12, 221–228.

Nobre, G. (1977). Sensitivity to 5-fluorocytosine and virulence for mice of some human isolates of Aspergillus. *Mycopathologia* 62, 57–60.

Oakley, K. L., Morrissey, G., and Denning, D. W. (1997). Efficacy of SCH-56592 in a temporarily neutropenic murine model of invasive aspergillosis with an itraconazole-susceptible and an itraconazole-resistant isolate of Aspergillus fumigatus. *Antimicrob. Agents Chemother.* 41, 1504–1507.

Odds, F. C., Oris, M., Van Dorsselaer, P., and Van Gerven, F. (2000). Activities of an intravenous formulation of itraconazole in experimental disseminated Aspergillus, Candida, and Cryptococcus infections. *Antimicrob. Agents Chemother.* 44, 3180–3183.

Odds, F. C., Van Gerven, F., Espinel-Ingroff, A., Bartlett, M. S., Ghannoum, M. A., Lancaster, M. V., et al. (1998). Evaluation of possible correlations between antifungal susceptibilities of filamentous fungi in vitro and antifungal treatment outcomes in animal infection models. *Antimicrob. Agents Chemother.* 42, 282–288.

O’Dea, E. M., Amarsaikhan, N., Li, H., Downey, J., Steele, E., Van Dyken, S. J., et al. (2014). Eosinophils are recruited in response to chitin exposure and enhance Th2-mediated immune pathology in Aspergillus fumigatus infection. *Infect. Immun.* 82, 3199–3205. doi:10.1128/IAI.01990-14.

O’Hanlon, K. A., Cairns, T., Stack, D., Schrettl, M., Bignell, E. M., Kavanagh, K., et al. (2011). Targeted disruption of nonribosomal peptide synthetase pes3 augments the virulence of Aspergillus fumigatus. *Infect. Immun.* 79, 3978–3992. doi:10.1128/IAI.00192-11.

Ohtsuka, K., Watanabe, M., Orikasa, Y., Inouye, S., Uchida, K., Yamaguchi, H., et al. (1997). The in-vivo activity of an antifungal antibiotic, benanomicin A, in comparison with amphotericin B and fluconazole. *J. Antimicrob. Chemother.* 39, 71–77.

Okamoto, T., Tanida, T., Wei, B., Ueta, E., Yamamoto, T., and Osaki, T. (2004). Regulation of fungal infection by a combination of amphotericin B and peptide 2, a lactoferrin peptide that activates neutrophils. *Clin. Diagn. Lab. Immunol.* 11, 1111–1119. doi:10.1128/CDLI.11.6.1111-1119.2004.

Okawa, Y., Murata, Y., Suzuki, M., Ito, M., Hotchi, M., and Suzuki, S. (2002). Delayed lethal response to Aspergillus fumigatus infection in sarcoma 180 tumor-bearing mice. *FEMS Immunol. Med. Microbiol.* 34, 113–117.

Okazaki, K.-I., Asakura, M., Sugimoto, N., Hinenoya, A., and Yamasaki, S. (2009). Candida albicans, Cryptococcus neoformans or Aspergillus fumigatus induces an antifungal activity in mouse serum, which is different from transferrin. *J. Vet. Med. Sci. Jpn. Soc. Vet. Sci.* 71, 1459–1464.

Oki, T., Kakushima, M., Hirano, M., Takahashi, A., Ohta, A., Masuyoshi, S., et al. (1992). In vitro and in vivo antifungal activities of BMS-181184. *J. Antibiot. (Tokyo)* 45, 1512–1517.

Olivas, I., Royuela, M., Romero, B., Monteiro, M. C., Mínguez, J. M., Laborda, F., et al. (2008). Ability to grow on lipids accounts for the fully virulent phenotype in neutropenic mice of Aspergillus fumigatus null mutants in the key glyoxylate cycle enzymes. *Fungal Genet. Biol. FG B* 45, 45–60. doi:10.1016/j.fgb.2007.05.002.

Oliver, J. D., Kaye, S. J., Tuckwell, D., Johns, A. E., Macdonald, D. A., Livermore, J., et al. (2012). The Aspergillus fumigatus dihydroxyacid dehydratase Ilv3A/IlvC is required for full virulence. *PloS One* 7, e43559. doi:10.1371/journal.pone.0043559.

Olson, J. A., Adler-Moore, J. P., Jensen, G. M., Schwartz, J., Dignani, M. C., and Proffitt, R. T. (2008). Comparison of the physicochemical, antifungal, and toxic properties of two liposomal amphotericin B products. *Antimicrob. Agents Chemother.* 52, 259–268. doi:10.1128/AAC.00870-07.

Olson, J. A., Adler-Moore, J. P., Schwartz, J., Jensen, G. M., and Proffitt, R. T. (2006). Comparative efficacies, toxicities, and tissue concentrations of amphotericin B lipid formulations in a murine pulmonary aspergillosis model. *Antimicrob. Agents Chemother.* 50, 2122–2131. doi:10.1128/AAC.00315-06.

Olson, J. A., George, A., Constable, D., Smith, P., Proffitt, R. T., and Adler-Moore, J. P. (2010). Liposomal amphotericin B and echinocandins as monotherapy or sequential or concomitant therapy in murine disseminated and pulmonary Aspergillus fumigatus infections. *Antimicrob. Agents Chemother.* 54, 3884–3894. doi:10.1128/AAC.01554-09.

Olson, J. A., Schwartz, J. A., Hahka, D., Nguyen, N., Bunch, T., Jensen, G. M., et al. (2015). Toxicity and efficacy differences between liposomal amphotericin B formulations in uninfected and Aspergillus fumigatus infected mice. *Med. Mycol.* 53, 107–118. doi:10.1093/mmy/myu070.

Otsubo, T., Maesaki, S., Hossain, M. A., Yamamoto, Y., Tomono, K., Tashiro, T., et al. (1999). In vitro and in vivo activities of NS-718, a new lipid nanosphere incorporating amphotericin B, against Aspergillus fumigatus. *Antimicrob. Agents Chemother.* 43, 471–475.

Otsubo, T., Maruyama, K., Maesaki, S., Miyazaki, Y., Tanaka, E., Takizawa, T., et al. (1998). Long-circulating immunoliposomal amphotericin B against invasive pulmonary aspergillosis in mice. *Antimicrob. Agents Chemother.* 42, 40–44.

Overdijk, B., Van Steijn, G. J., and Odds, F. C. (1996). Chitinase levels in guinea pig blood are increased after systemic infection with Aspergillus fumigatus. *Glycobiology* 6, 627–634.

Overdijk, B., Van Steijn, G. J., and Odds, F. C. (1999). Distribution of chitinase in guinea pig tissues and increases in levels of this enzyme after systemic infection with Aspergillus fumigatus. *Microbiol. Read. Engl.* 145 ( Pt 1), 259–269. doi:10.1099/13500872-145-1-259.

Owais, M., Ahmed, I., Krishnakumar, B., Jain, R. K., Bachhawat, B. K., and Gupta, C. M. (1993). Tuftsin-bearing liposomes as drug vehicles in the treatment of experimental aspergillosis. *FEBS Lett.* 326, 56–58.

Paisley, D., Robson, G. D., and Denning, D. W. (2005). Correlation between in vitro growth rate and in vivo virulence in Aspergillus fumigatus. *Med. Mycol.* 43, 397–401.

Panepinto, J. C., Oliver, B. G., Fortwendel, J. R., Smith, D. L. H., Askew, D. S., and Rhodes, J. C. (2003). Deletion of the Aspergillus fumigatus gene encoding the Ras-related protein RhbA reduces virulence in a model of Invasive pulmonary aspergillosis. *Infect. Immun.* 71, 2819–2826.

Pardo, J., Urban, C., Galvez, E. M., Ekert, P. G., Müller, U., Kwon-Chung, J., et al. (2006). The mitochondrial protein Bak is pivotal for gliotoxin-induced apoptosis and a critical host factor of Aspergillus fumigatus virulence in mice. *J. Cell Biol.* 174, 509–519. doi:10.1083/jcb.200604044.

Paris, S., Monod, M., Diaquin, M., Lamy, B., Arruda, L. K., Punt, P. J., et al. (1993). A transformant of Aspergillus fumigatus deficient in the antigenic cytotoxin ASPFI. *FEMS Microbiol. Lett.* 111, 31–36.

Paris, S., Wysong, D., Debeaupuis, J.-P., Shibuya, K., Philippe, B., Diamond, R. D., et al. (2003). Catalases of Aspergillus fumigatus. *Infect. Immun.* 71, 3551–3562.

Park, S. J., Burdick, M. D., Brix, W. K., Stoler, M. H., Askew, D. S., Strieter, R. M., et al. (2010). Neutropenia enhances lung dendritic cell recruitment in response to Aspergillus via a cytokine-to-chemokine amplification loop. *J. Immunol. Baltim. Md 1950* 185, 6190–6197. doi:10.4049/jimmunol.1002064.

Park, S. J., Hughes, M. A., Burdick, M., Strieter, R. M., and Mehrad, B. (2009). Early NK cell-derived IFN-{gamma} is essential to host defense in neutropenic invasive aspergillosis. *J. Immunol. Baltim. Md 1950* 182, 4306–4312. doi:10.4049/jimmunol.0803462.

Patera, A. C., Menzel, F., Jackson, C., Brieland, J. K., Halpern, J., Hare, R., et al. (2004). Effect of granulocyte colony-stimulating factor combination therapy on efficacy of posaconazole (SCH56592) in an inhalation model of murine pulmonary aspergillosis. *Antimicrob. Agents Chemother.* 48, 3154–3158. doi:10.1128/AAC.48.8.3154-3158.2004.

Paulussen, C., Boulet, G., Bosschaerts, T., Cos, P., Fortin, A., and Maes, L. (2015). Efficacy of oleylphosphocholine (OlPC) in vitro and in a mouse model of invasive aspergillosis. *Mycoses* 58, 127–132. doi:10.1111/myc.12286.

Petersen, J. E., Hiran, T. S., Goebel, W. S., Johnson, C., Murphy, R. C., Azmi, F. H., et al. (2002). Enhanced cutaneous inflammatory reactions to Aspergillus fumigatus in a murine model of chronic granulomatous disease. *J. Invest. Dermatol.* 118, 424–429. doi:10.1046/j.0022-202x.2001.01691.x.

Petrik, M., Franssen, G. M., Haas, H., Laverman, P., Hörtnagl, C., Schrettl, M., et al. (2012). Preclinical evaluation of two 68Ga-siderophores as potential radiopharmaceuticals for Aspergillus fumigatus infection imaging. *Eur. J. Nucl. Med. Mol. Imaging* 39, 1175–1183. doi:10.1007/s00259-012-2110-3.

Petrik, M., Haas, H., Dobrozemsky, G., Lass-Flörl, C., Helbok, A., Blatzer, M., et al. (2010). 68Ga-siderophores for PET imaging of invasive pulmonary aspergillosis: proof of principle. *J. Nucl. Med. Off. Publ. Soc. Nucl. Med.* 51, 639–645. doi:10.2967/jnumed.109.072462.

Petrik, M., Haas, H., Laverman, P., Schrettl, M., Franssen, G. M., Blatzer, M., et al. (2014). 68Ga-triacetylfusarinine C and 68Ga-ferrioxamine E for Aspergillus infection imaging: uptake specificity in various microorganisms. *Mol. Imaging Biol. MIB Off. Publ. Acad. Mol. Imaging* 16, 102–108. doi:10.1007/s11307-013-0654-7.

Phadke, A. P., Akangire, G., Park, S. J., Lira, S. A., and Mehrad, B. (2007). The role of CC chemokine receptor 6 in host defense in a model of invasive pulmonary aspergillosis. *Am. J. Respir. Crit. Care Med.* 175, 1165–1172. doi:10.1164/rccm.200602-256OC.

Philippe, B., Ibrahim-Granet, O., Prévost, M. C., Gougerot-Pocidalo, M. A., Sanchez Perez, M., Van der Meeren, A., et al. (2003). Killing of Aspergillus fumigatus by alveolar macrophages is mediated by reactive oxidant intermediates. *Infect. Immun.* 71, 3034–3042.

Pinchai, N., Juvvadi, P. R., Fortwendel, J. R., Perfect, B. Z., Rogg, L. E., Asfaw, Y. G., et al. (2010). The Aspergillus fumigatus P-type Golgi apparatus Ca2+/Mn2+ ATPase PmrA is involved in cation homeostasis and cell wall integrity but is not essential for pathogenesis. *Eukaryot. Cell* 9, 472–476. doi:10.1128/EC.00378-09.

Pinchai, N., Perfect, B. Z., Juvvadi, P. R., Fortwendel, J. R., Cramer, R. A., Asfaw, Y. G., et al. (2009). Aspergillus fumigatus calcipressin CbpA is involved in hyphal growth and calcium homeostasis. *Eukaryot. Cell* 8, 511–519. doi:10.1128/EC.00336-08.

Plempel, M. (1984). Antimycotic activity of BAY N 7133 in animal experiments. *J. Antimicrob. Chemother.* 13, 447–463.

Poelmans, J., Hillen, A., Vanherp, L., Govaerts, K., Maertens, J., Dresselaers, T., et al. (2016). Longitudinal, in vivo assessment of invasive pulmonary aspergillosis in mice by computed tomography and magnetic resonance imaging. *Lab. Investig. J. Tech. Methods Pathol.* 96, 692–704. doi:10.1038/labinvest.2016.45.

Polak, A. (1982). Oxiconazole, a new imidazole derivative. Evaluation of antifungal activity in vitro and in vivo. *Arzneimittelforschung.* 32, 17–24.

Polak, A. (1987). Combination therapy of experimental candidiasis, cryptococcosis, aspergillosis and wangiellosis in mice. *Chemotherapy* 33, 381–395.

Polak, A., Scholer, H. J., and Wall, M. (1982). Combination therapy of experimental candidiasis, cryptococcosis and aspergillosis in mice. *Chemotherapy* 28, 461–479.

Polak-Wyss, A. (1991). Protective effect of human granulocyte colony-stimulating factor (hG-CSF) on Cryptococcus and Aspergillus infections in normal and immunosuppressed mice. *Mycoses* 34, 205–215.

Pollock, J. D., Williams, D. A., Gifford, M. A., Li, L. L., Du, X., Fisherman, J., et al. (1995). Mouse model of X-linked chronic granulomatous disease, an inherited defect in phagocyte superoxide production. *Nat. Genet.* 9, 202–209. doi:10.1038/ng0295-202.

Pongpom, M., Liu, H., Xu, W., Snarr, B. D., Sheppard, D. C., Mitchell, A. P., et al. (2015). Divergent targets of Aspergillus fumigatus AcuK and AcuM transcription factors during growth in vitro versus invasive disease. *Infect. Immun.* 83, 923–933. doi:10.1128/IAI.02685-14.

Pope, A. M., and Davies, D. A. (1979). The influence of carbohydrases on the growth of fungal pathogens in vitro and in vivo. *Postgrad. Med. J.* 55, 674–676.

Powers-Fletcher, M. V., Jambunathan, K., Brewer, J. L., Krishnan, K., Feng, X., Galande, A. K., et al. (2011). Impact of the lectin chaperone calnexin on the stress response, virulence and proteolytic secretome of the fungal pathogen Aspergillus fumigatus. *PloS One* 6, e28865. doi:10.1371/journal.pone.0028865.

Prüfer, S., Weber, M., Stein, P., Bosmann, M., Stassen, M., Kreft, A., et al. (2014). Oxidative burst and neutrophil elastase contribute to clearance of Aspergillus fumigatus pneumonia in mice. *Immunobiology* 219, 87–96. doi:10.1016/j.imbio.2013.08.010.

Puttikamonkul, S., Willger, S. D., Grahl, N., Perfect, J. R., Movahed, N., Bothner, B., et al. (2010). Trehalose 6-phosphate phosphatase is required for cell wall integrity and fungal virulence but not trehalose biosynthesis in the human fungal pathogen Aspergillus fumigatus. *Mol. Microbiol.* 77, 891–911. doi:10.1111/j.1365-2958.2010.07254.x.

Qiao, J., Kontoyiannis, D. P., Calderone, R., Li, D., Ma, Y., Wan, Z., et al. (2008). Afyap1, encoding a bZip transcriptional factor of Aspergillus fumigatus, contributes to oxidative stress response but is not essential to the virulence of this pathogen in mice immunosuppressed by cyclophosphamide and triamcinolone. *Med. Mycol.* 46, 773–782. doi:10.1080/13693780802054215.

Quezada, G., Koshkina, N. V., Zweidler-McKay, P., Zhou, Z., Kontoyiannis, D. P., and Kleinerman, E. S. (2008). Intranasal granulocyte-macrophage colony-stimulating factor reduces the Aspergillus burden in an immunosuppressed murine model of pulmonary aspergillosis. *Antimicrob. Agents Chemother.* 52, 716–718. doi:10.1128/AAC.00760-07.

Raghuwanshi, S. K., Kumar, M., Kavishwar, A., Chaturvedi, A. K., Murthy, P. S. R., and Shukla, P. K. (2005). Immunolocalization of secretory proteins of Aspergillus fumigatus using monospecific polyclonal antibodies in a murine model. *Mycoses* 48, 313–320. doi:10.1111/j.1439-0507.2005.01141.x.

Rajendran, R., Mowat, E., McCulloch, E., Lappin, D. F., Jones, B., Lang, S., et al. (2011). Azole resistance of Aspergillus fumigatus biofilms is partly associated with efflux pump activity. *Antimicrob. Agents Chemother.* 55, 2092–2097. doi:10.1128/AAC.01189-10.

Ramaprakash, H., Ito, T., Standiford, T. J., Kunkel, S. L., and Hogaboam, C. M. (2009). Toll-like receptor 9 modulates immune responses to Aspergillus fumigatus conidia in immunodeficient and allergic mice. *Infect. Immun.* 77, 108–119. doi:10.1128/IAI.00998-08.

Ramirez-Ortiz, Z. G., Lee, C. K., Wang, J. P., Boon, L., Specht, C. A., and Levitz, S. M. (2011). A nonredundant role for plasmacytoid dendritic cells in host defense against the human fungal pathogen Aspergillus fumigatus. *Cell Host Microbe* 9, 415–424. doi:10.1016/j.chom.2011.04.007.

Rammaert, B., Jouvion, G., de Chaumont, F., Garcia-Hermoso, D., Szczepaniak, C., Renaudat, C., et al. (2015). Absence of Fungal Spore Internalization by Bronchial Epithelium in Mouse Models Evidenced by a New Bioimaging Approach and Transmission Electronic Microscopy. *Am. J. Pathol.* 185, 2421–2430. doi:10.1016/j.ajpath.2015.04.027.

Rebong, R. A., Santaella, R. M., Goldhagen, B. E., Majka, C. P., Perfect, J. R., Steinbach, W. J., et al. (2011). Polyhexamethylene biguanide and calcineurin inhibitors as novel antifungal treatments for Aspergillus keratitis. *Invest. Ophthalmol. Vis. Sci.* 52, 7309–7315. doi:10.1167/iovs.11-7739.

Reichard, U., Monod, M., Odds, F., and Rüchel, R. (1997). Virulence of an aspergillopepsin-deficient mutant of Aspergillus fumigatus and evidence for another aspartic proteinase linked to the fungal cell wall. *J. Med. Vet. Mycol. Bi-Mon. Publ. Int. Soc. Hum. Anim. Mycol.* 35, 189–196.

Ren, S., Zhang, F., Li, C., Jia, C., Li, S., Xi, H., et al. (2010). Selection of housekeeping genes for use in quantitative reverse transcription PCR assays on the murine cornea. *Mol. Vis.* 16, 1076–1086.

Renshaw, H., Vargas-Muñiz, J. M., Richards, A. D., Asfaw, Y. G., Juvvadi, P. R., and Steinbach, W. J. (2016). Distinct Roles of Myosins in Aspergillus fumigatus Hyphal Growth and Pathogenesis. *Infect. Immun.* 84, 1556–1564. doi:10.1128/IAI.01190-15.

Richie, D. L., Feng, X., Hartl, L., Aimanianda, V., Krishnan, K., Powers-Fletcher, M. V., et al. (2011). The virulence of the opportunistic fungal pathogen Aspergillus fumigatus requires cooperation between the endoplasmic reticulum-associated degradation pathway (ERAD) and the unfolded protein response (UPR). *Virulence* 2, 12–21.

Richie, D. L., Fuller, K. K., Fortwendel, J., Miley, M. D., McCarthy, J. W., Feldmesser, M., et al. (2007a). Unexpected link between metal ion deficiency and autophagy in Aspergillus fumigatus. *Eukaryot. Cell* 6, 2437–2447. doi:10.1128/EC.00224-07.

Richie, D. L., Hartl, L., Aimanianda, V., Winters, M. S., Fuller, K. K., Miley, M. D., et al. (2009). A role for the unfolded protein response (UPR) in virulence and antifungal susceptibility in Aspergillus fumigatus. *PLoS Pathog.* 5, e1000258. doi:10.1371/journal.ppat.1000258.

Richie, D. L., Miley, M. D., Bhabhra, R., Robson, G. D., Rhodes, J. C., and Askew, D. S. (2007b). The Aspergillus fumigatus metacaspases CasA and CasB facilitate growth under conditions of endoplasmic reticulum stress. *Mol. Microbiol.* 63, 591–604. doi:10.1111/j.1365-2958.2006.05534.x.

Rieber, N., Singh, A., Öz, H., Carevic, M., Bouzani, M., Amich, J., et al. (2015). Pathogenic fungi regulate immunity by inducing neutrophilic myeloid-derived suppressor cells. *Cell Host Microbe* 17, 507–514. doi:10.1016/j.chom.2015.02.007.

Rieg, G., Spellberg, B., Schwartz, J., Fu, Y., Edwards, J. E., Sheppard, D. C., et al. (2006). Antifungal prophylaxis is effective against murine invasive pulmonary aspergillosis. *Antimicrob. Agents Chemother.* 50, 2895–2896. doi:10.1128/AAC.00299-06.

Risovic, V., Rosland, M., Sivak, O., Wasan, K. M., and Bartlett, K. (2007). Assessing the antifungal activity of a new oral lipid-based amphotericin B formulation following administration to rats infected with Aspergillus fumigatus. *Drug Dev. Ind. Pharm.* 33, 703–707. doi:10.1080/03639040601077349.

Rivera, A., Collins, N., Stephan, M. T., Lipuma, L., Leiner, I., and Pamer, E. G. (2009). Aberrant tissue localization of fungus-specific CD4+ T cells in IL-10-deficient mice. *J. Immunol. Baltim. Md 1950* 183, 631–641. doi:10.4049/jimmunol.0900396.

Rivera, A., Hohl, T. M., Collins, N., Leiner, I., Gallegos, A., Saijo, S., et al. (2011). Dectin-1 diversifies Aspergillus fumigatus-specific T cell responses by inhibiting T helper type 1 CD4 T cell differentiation. *J. Exp. Med.* 208, 369–381. doi:10.1084/jem.20100906.

Rivera, A., Ro, G., Van Epps, H. L., Simpson, T., Leiner, I., Sant’Angelo, D. B., et al. (2006). Innate immune activation and CD4+ T cell priming during respiratory fungal infection. *Immunity* 25, 665–675. doi:10.1016/j.immuni.2006.08.016.

Rivera, A., Van Epps, H. L., Hohl, T. M., Rizzuto, G., and Pamer, E. G. (2005). Distinct CD4+-T-cell responses to live and heat-inactivated Aspergillus fumigatus conidia. *Infect. Immun.* 73, 7170–7179. doi:10.1128/IAI.73.11.7170-7179.2005.

Rizzetto, L., Giovannini, G., Bromley, M., Bowyer, P., Romani, L., and Cavalieri, D. (2013). Strain dependent variation of immune responses to A. fumigatus: definition of pathogenic species. *PloS One* 8, e56651. doi:10.1371/journal.pone.0056651.

Rodriguez, T. E., Falkowski, N. R., Harkema, J. R., and Huffnagle, G. B. (2007). Role of neutrophils in preventing and resolving acute fungal sinusitis. *Infect. Immun.* 75, 5663–5668. doi:10.1128/IAI.01542-06.

Röhm, M., Grimm, M. J., D’Auria, A. C., Almyroudis, N. G., Segal, B. H., and Urban, C. F. (2014). NADPH oxidase promotes neutrophil extracellular trap formation in pulmonary aspergillosis. *Infect. Immun.* 82, 1766–1777. doi:10.1128/IAI.00096-14.

Rolle, A.-M., Hasenberg, M., Thornton, C. R., Solouk-Saran, D., Männ, L., Weski, J., et al. (2016). ImmunoPET/MR imaging allows specific detection of Aspergillus fumigatus lung infection in vivo. *Proc. Natl. Acad. Sci. U. S. A.* 113, E1026-1033. doi:10.1073/pnas.1518836113.

Romani, L., Bistoni, F., Gaziano, R., Bozza, S., Montagnoli, C., Perruccio, K., et al. (2004). Thymosin alpha 1 activates dendritic cells for antifungal Th1 resistance through toll-like receptor signaling. *Blood* 103, 4232–4239. doi:10.1182/blood-2003-11-4036.

Romani, L., Bistoni, F., Perruccio, K., Montagnoli, C., Gaziano, R., Bozza, S., et al. (2006). Thymosin alpha1 activates dendritic cell tryptophan catabolism and establishes a regulatory environment for balance of inflammation and tolerance. *Blood* 108, 2265–2274. doi:10.1182/blood-2006-02-004762.

Romani, L., Fallarino, F., De Luca, A., Montagnoli, C., D’Angelo, C., Zelante, T., et al. (2008). Defective tryptophan catabolism underlies inflammation in mouse chronic granulomatous disease. *Nature* 451, 211–215. doi:10.1038/nature06471.

Romano, J., Nimrod, G., Ben-Tal, N., Shadkchan, Y., Baruch, K., Sharon, H., et al. (2006). Disruption of the Aspergillus fumigatus ECM33 homologue results in rapid conidial germination, antifungal resistance and hypervirulence. *Microbiol. Read. Engl.* 152, 1919–1928. doi:10.1099/mic.0.28936-0.

Rubino, I., Coste, A., Le Roy, D., Roger, T., Jaton, K., Boeckh, M., et al. (2012). Species-specific recognition of Aspergillus fumigatus by Toll-like receptor 1 and Toll-like receptor 6. *J. Infect. Dis.* 205, 944–954. doi:10.1093/infdis/jir882.

Rüchel, R., Schaffrinski, M., Seshan, K. R., and Cole, G. T. (2000). Vital staining of fungal elements in deep-seated mycotic lesions during experimental murine mycoses using the parenterally applied optical brightener Blankophor. *Med. Mycol.* 38, 231–237.

Ruijgrok, E. J., Fens, M. H. A., Bakker-Woudenberg, I. A. J. M., van Etten, E. W. M., and Vulto, A. G. (2005). Nebulization of four commercially available amphotericin B formulations in persistently granulocytopenic rats with invasive pulmonary aspergillosis: evidence for long-term biological activity. *J. Pharm. Pharmacol.* 57, 1289–1295. doi:10.1211/jpp.57.10.0007.

Ruijgrok, E. J., Fens, M. H. A. M., Bakker-Woudenberg, I. A. J. M., van Etten, E. W. M., and Vulto, A. G. (2006). Nebulized amphotericin B combined with intravenous amphotericin B in rats with severe invasive pulmonary aspergillosis. *Antimicrob. Agents Chemother.* 50, 1852–1854. doi:10.1128/AAC.50.5.1852-1854.2006.

Ruijgrok, E. J., Vulto, A. G., and Van Etten, E. W. (2001). Efficacy of aerosolized amphotericin B desoxycholate and liposomal amphotericin B in the treatment of invasive pulmonary aspergillosis in severely immunocompromised rats. *J. Antimicrob. Chemother.* 48, 89–95.

Ruiz-Cabello, J., Regadera, J., Santisteban, C., Graña, M., Pérez de Alejo, R., Echave, I., et al. (2002). Monitoring acute inflammatory processes in mouse muscle by MR imaging and spectroscopy: a comparison with pathological results. *NMR Biomed.* 15, 204–214. doi:10.1002/nbm.761.

Saeed, E. N., and Hay, R. J. (1981). Immunoperoxidase staining in the recognition of Aspergillus infections. *Histopathology* 5, 437–444.

Salas, V., Pastor, F. J., Calvo, E., Sutton, D. A., Fothergill, A. W., and Guarro, J. (2013). Evaluation of the in vitro activity of voriconazole as predictive of in vivo outcome in a murine Aspergillus fumigatus infection model. *Antimicrob. Agents Chemother.* 57, 1404–1408. doi:10.1128/AAC.01331-12.

Sandhu, D. K., Sandhu, R. S., Damodaran, V. N., and Randhawa, H. S. (1970). Effect of cortisone on bronchopulmonary aspergillosis in mice exposed to spores of various Aspergillus species. *Sabouraudia* 8, 32–38.

Sandhu, D. K., Sandhu, R. S., Khan, Z. U., and Damodaran, V. N. (1976). Conditional virulence of a p-aminobenzoic acid-requiring mutant of Aspergillus fumigatus. *Infect. Immun.* 13, 527–532.

Sarfati, J., Diaquin, M., Debeaupuis, J. P., Schmidt, A., Lecaque, D., Beauvais, A., et al. (2002). A new experimental murine aspergillosis model to identify strains of Aspergillus fumigatus with reduced virulence. *Nihon Ishinkin Gakkai Zasshi Jpn. J. Med. Mycol.* 43, 203–213.

Sasse, C., Bignell, E. M., Hasenberg, M., Haynes, K., Gunzer, M., Braus, G. H., et al. (2008). Basal expression of the Aspergillus fumigatus transcriptional activator CpcA is sufficient to support pulmonary aspergillosis. *Fungal Genet. Biol. FG B* 45, 693–704. doi:10.1016/j.fgb.2007.12.008.

Savers, A., Rasid, O., Parlato, M., Brock, M., Jouvion, G., Ryffel, B., et al. (2016). Infection-Mediated Priming of Phagocytes Protects against Lethal Secondary Aspergillus fumigatus Challenge. *PloS One* 11, e0153829. doi:10.1371/journal.pone.0153829.

Saxena, S., and Ghosh, P. C. (2000). Biodistribution of amphotericin B when delivered through cholesterol hemisuccinate vesicles in normal and A. fumigatus infected mice. *Pharm. Res.* 17, 1236–1242.

Saxena, S., Khan, J. A., and Ghosh, P. C. (1998). Toxicity and therapeutic efficacy of amphotericin B delivered through cholesterol hemisuccinate vesicles in the treatment of experimental murine aspergillosis. *J. Antimicrob. Chemother.* 42, 635–642.

Schaffner, A., and Böhler, A. (1993). Amphotericin B refractory aspergillosis after itraconazole: evidence for significant antagonism. *Mycoses* 36, 421–424.

Schaffner, A., Douglas, H., and Braude, A. (1982). Selective protection against conidia by mononuclear and against mycelia by polymorphonuclear phagocytes in resistance to Aspergillus. Observations on these two lines of defense in vivo and in vitro with human and mouse phagocytes. *J. Clin. Invest.* 69, 617–631.

Schaffner, A., and Frick, P. G. (1985). The effect of ketoconazole on amphotericin B in a model of disseminated aspergillosis. *J. Infect. Dis.* 151, 902–910.

Schaude, M., Petranyi, G., Ackerbauer, H., Meingassner, J. G., and Mieth, H. (1990). Preclinical antimycotic activity of SDZ 89-485: a new orally and topically effective triazole. *J. Med. Vet. Mycol. Bi-Mon. Publ. Int. Soc. Hum. Anim. Mycol.* 28, 445–454.

Schlitzer, A., McGovern, N., Teo, P., Zelante, T., Atarashi, K., Low, D., et al. (2013). IRF4 transcription factor-dependent CD11b+ dendritic cells in human and mouse control mucosal IL-17 cytokine responses. *Immunity* 38, 970–983. doi:10.1016/j.immuni.2013.04.011.

Schmalhorst, P. S., Krappmann, S., Vervecken, W., Rohde, M., Müller, M., Braus, G. H., et al. (2008). Contribution of galactofuranose to the virulence of the opportunistic pathogen Aspergillus fumigatus. *Eukaryot. Cell* 7, 1268–1277. doi:10.1128/EC.00109-08.

Schmitt, H. J., Andrade, J., Edwards, F., Niki, Y., Bernard, E., and Armstrong, D. (1990). Inactivity of terbinafine in a rat model of pulmonary aspergillosis. *Eur. J. Clin. Microbiol. Infect. Dis. Off. Publ. Eur. Soc. Clin. Microbiol.* 9, 832–835.

Schmitt, H. J., Bernard, E. M., Edwards, F. F., and Armstrong, D. (1991). Combination therapy in a model of pulmonary aspergillosis. *Mycoses* 34, 281–285.

Schmitt, H. J., Bernard, E. M., Häuser, M., and Armstrong, D. (1988). Aerosol amphotericin B is effective for prophylaxis and therapy in a rat model of pulmonary aspergillosis. *Antimicrob. Agents Chemother.* 32, 1676–1679.

Schmitt, H. J., Edwards, F., Andrade, J., Niki, Y., and Armstrong, D. (1992). Comparison of azoles against aspergilli in vitro and in an experimental model of pulmonary aspergillosis. *Chemotherapy* 38, 118–126.

Schöbel, F., Ibrahim-Granet, O., Avé, P., Latgé, J.-P., Brakhage, A. A., and Brock, M. (2007). Aspergillus fumigatus does not require fatty acid metabolism via isocitrate lyase for development of invasive aspergillosis. *Infect. Immun.* 75, 1237–1244. doi:10.1128/IAI.01416-06.

Schöbel, F., Jacobsen, I. D., and Brock, M. (2010). Evaluation of lysine biosynthesis as an antifungal drug target: biochemical characterization of Aspergillus fumigatus homocitrate synthase and virulence studies. *Eukaryot. Cell* 9, 878–893. doi:10.1128/EC.00020-10.

Schrettl, M., Beckmann, N., Varga, J., Heinekamp, T., Jacobsen, I. D., Jöchl, C., et al. (2010a). HapX-mediated adaption to iron starvation is crucial for virulence of Aspergillus fumigatus. *PLoS Pathog.* 6, e1001124. doi:10.1371/journal.ppat.1001124.

Schrettl, M., Bignell, E., Kragl, C., Joechl, C., Rogers, T., Arst, H. N., et al. (2004). Siderophore biosynthesis but not reductive iron assimilation is essential for Aspergillus fumigatus virulence. *J. Exp. Med.* 200, 1213–1219. doi:10.1084/jem.20041242.

Schrettl, M., Bignell, E., Kragl, C., Sabiha, Y., Loss, O., Eisendle, M., et al. (2007). Distinct roles for intra- and extracellular siderophores during Aspergillus fumigatus infection. *PLoS Pathog.* 3, 1195–1207. doi:10.1371/journal.ppat.0030128.

Schrettl, M., Ibrahim-Granet, O., Droin, S., Huerre, M., Latgé, J.-P., and Haas, H. (2010b). The crucial role of the Aspergillus fumigatus siderophore system in interaction with alveolar macrophages. *Microbes Infect. Inst. Pasteur* 12, 1035–1041. doi:10.1016/j.micinf.2010.07.005.

Schütte, M., Thullier, P., Pelat, T., Wezler, X., Rosenstock, P., Hinz, D., et al. (2009). Identification of a putative Crf splice variant and generation of recombinant antibodies for the specific detection of Aspergillus fumigatus. *PloS One* 4, e6625. doi:10.1371/journal.pone.0006625.

Scotter, J. M., and Chambers, S. T. (2005). Comparison of galactomannan detection, PCR-enzyme-linked immunosorbent assay, and real-time PCR for diagnosis of invasive aspergillosis in a neutropenic rat model and effect of caspofungin acetate. *Clin. Diagn. Lab. Immunol.* 12, 1322–1327. doi:10.1128/CDLI.12.11.1322-1327.2005.

Sekonyela, R., Palmer, J. M., Bok, J.-W., Jain, S., Berthier, E., Forseth, R., et al. (2013). RsmA regulates Aspergillus fumigatus gliotoxin cluster metabolites including cyclo(L-Phe-L-Ser), a potential new diagnostic marker for invasive aspergillosis. *PloS One* 8, e62591. doi:10.1371/journal.pone.0062591.

Severin, G. W., Jørgensen, J. T., Wiehr, S., Rolle, A.-M., Hansen, A. E., Maurer, A., et al. (2015). The impact of weakly bound ^89^Zr on preclinical studies: non-specific accumulation in solid tumors and aspergillus infection. *Nucl. Med. Biol.* 42, 360–368. doi:10.1016/j.nucmedbio.2014.11.005.

Seyedmousavi, S., Brüggemann, R. J. M., Meis, J. F., Melchers, W. J. G., Verweij, P. E., and Mouton, J. W. (2015a). Pharmacodynamics of isavuconazole in an Aspergillus fumigatus mouse infection model. *Antimicrob. Agents Chemother.* 59, 2855–2866. doi:10.1128/AAC.04907-14.

Seyedmousavi, S., Brüggemann, R. J. M., Melchers, W. J. G., Rijs, A. J. M. M., Verweij, P. E., and Mouton, J. W. (2013a). Efficacy and pharmacodynamics of voriconazole combined with anidulafungin in azole-resistant invasive aspergillosis. *J. Antimicrob. Chemother.* 68, 385–393. doi:10.1093/jac/dks402.

Seyedmousavi, S., Brüggemann, R. J. M., Melchers, W. J. G., Verweij, P. E., and Mouton, J. W. (2013b). Pharmacodynamics of anidulafungin against clinical Aspergillus fumigatus isolates in a nonneutropenic murine model of disseminated aspergillosis. *Antimicrob. Agents Chemother.* 57, 303–308. doi:10.1128/AAC.01430-12.

Seyedmousavi, S., Brüggemann, R. J. M., Melchers, W. J. G., Verweij, P. E., and Mouton, J. W. (2014). Intrapulmonary posaconazole penetration at the infection site in an immunosuppressed murine model of invasive pulmonary aspergillosis receiving oral prophylactic regimens. *Antimicrob. Agents Chemother.* 58, 2964–2967. doi:10.1128/AAC.00053-14.

Seyedmousavi, S., Melchers, W. J. G., Mouton, J. W., and Verweij, P. E. (2013c). Pharmacodynamics and dose-response relationships of liposomal amphotericin B against different azole-resistant Aspergillus fumigatus isolates in a murine model of disseminated aspergillosis. *Antimicrob. Agents Chemother.* 57, 1866–1871. doi:10.1128/AAC.02226-12.

Seyedmousavi, S., Mouton, J. W., Melchers, W. J. G., and Verweij, P. E. (2015b). Posaconazole prophylaxis in experimental azole-resistant invasive pulmonary aspergillosis. *Antimicrob. Agents Chemother.* 59, 1487–1494. doi:10.1128/AAC.03850-14.

Shadkchan, Y., Shemesh, E., Mirelman, D., Miron, T., Rabinkov, A., Wilchek, M., et al. (2004). Efficacy of allicin, the reactive molecule of garlic, in inhibiting Aspergillus spp. in vitro, and in a murine model of disseminated aspergillosis. *J. Antimicrob. Chemother.* 53, 832–836. doi:10.1093/jac/dkh174.

Shao, C., Qu, J., He, L., Zhang, Y., Wang, J., Wang, Y., et al. (2005a). Transient overexpression of gamma interferon promotes Aspergillus clearance in invasive pulmonary aspergillosis. *Clin. Exp. Immunol.* 142, 233–241. doi:10.1111/j.1365-2249.2005.02828.x.

Shao, C., Qu, J., He, L., Zhang, Y., Wang, J., Zhou, H., et al. (2005b). Dendritic cells transduced with an adenovirus vector encoding interleukin-12 are a potent vaccine for invasive pulmonary aspergillosis. *Genes Immun.* 6, 103–114. doi:10.1038/sj.gene.6364167.

Sharon, H., Hagag, S., and Osherov, N. (2009). Transcription factor PrtT controls expression of multiple secreted proteases in the human pathogenic mold Aspergillus fumigatus. *Infect. Immun.* 77, 4051–4060. doi:10.1128/IAI.00426-09.

Shepardson, K. M., Jhingran, A., Caffrey, A., Obar, J. J., Suratt, B. T., Berwin, B. L., et al. (2014). Myeloid derived hypoxia inducible factor 1-alpha is required for protection against pulmonary Aspergillus fumigatus infection. *PLoS Pathog.* 10, e1004378. doi:10.1371/journal.ppat.1004378.

Shepardson, K. M., Ngo, L. Y., Aimanianda, V., Latgé, J.-P., Barker, B. M., Blosser, S. J., et al. (2013). Hypoxia enhances innate immune activation to Aspergillus fumigatus through cell wall modulation. *Microbes Infect. Inst. Pasteur* 15, 259–269. doi:10.1016/j.micinf.2012.11.010.

Sheppard, D. C., Graybill, J. R., Najvar, L. K., Chiang, L. Y., Doedt, T., Kirkpatrick, W. R., et al. (2006a). Standardization of an experimental murine model of invasive pulmonary aspergillosis. *Antimicrob. Agents Chemother.* 50, 3501–3503. doi:10.1128/AAC.00787-06.

Sheppard, D. C., Marr, K. A., Fredricks, D. N., Chiang, L. Y., Doedt, T., and Filler, S. G. (2006b). Comparison of three methodologies for the determination of pulmonary fungal burden in experimental murine aspergillosis. *Clin. Microbiol. Infect. Off. Publ. Eur. Soc. Clin. Microbiol. Infect. Dis.* 12, 376–380. doi:10.1111/j.1469-0691.2005.01349.x.

Sheppard, D. C., Rieg, G., Chiang, L. Y., Filler, S. G., Edwards, J. E., Jr, and Ibrahim, A. S. (2004). Novel inhalational murine model of invasive pulmonary aspergillosis. *Antimicrob. Agents Chemother.* 48, 1908–1911.

Shibata, T., Habiel, D. M., Coelho, A. L., and Hogaboam, C. M. (2014). Axl receptor blockade protects from invasive pulmonary aspergillosis in mice. *J. Immunol. Baltim. Md 1950* 193, 3559–3565. doi:10.4049/jimmunol.1401258.

Shibuya, K., Takaoka, M., Uchida, K., Wakayama, M., Yamaguchi, H., Takahashi, K., et al. (1999). Histopathology of experimental invasive pulmonary aspergillosis in rats: pathological comparison of pulmonary lesions induced by specific virulent factor deficient mutants. *Microb. Pathog.* 27, 123–131. doi:10.1006/mpat.1999.0288.

Shirkhani, K., Teo, I., Armstrong-James, D., and Shaunak, S. (2015). Nebulised amphotericin B-polymethacrylic acid nanoparticle prophylaxis prevents invasive aspergillosis. *Nanomedicine Nanotechnol. Biol. Med.* 11, 1217–1226. doi:10.1016/j.nano.2015.02.012.

Siaens, R., Eijsink, V. G. H., Dierckx, R., and Slegers, G. (2004). (123)I-Labeled chitinase as specific radioligand for in vivo detection of fungal infections in mice. *J. Nucl. Med. Off. Publ. Soc. Nucl. Med.* 45, 1209–1216.

Singh, G., Imai, J., Clemons, K. V., and Stevens, D. A. (2005). Efficacy of caspofungin against central nervous system Aspergillus fumigatus infection in mice determined by TaqMan PCR and CFU methods. *Antimicrob. Agents Chemother.* 49, 1369–1376. doi:10.1128/AAC.49.4.1369-1376.2005.

Singh, M., Madan, T., Waters, P., Sonar, S., Singh, S. K., Kamran, M. F., et al. (2009). Therapeutic effects of recombinant forms of full-length and truncated human surfactant protein D in a murine model of invasive pulmonary aspergillosis. *Mol. Immunol.* 46, 2363–2369. doi:10.1016/j.molimm.2009.03.019.

Singh, S., Dabur, R., Gatne, M. M., Singh, B., Gupta, S., Pawar, S., et al. (2014). In vivo efficacy of a synthetic coumarin derivative in a murine model of aspergillosis. *PloS One* 9, e103039. doi:10.1371/journal.pone.0103039.

Sionov, E., Mendlovic, S., and Segal, E. (2005). Experimental systemic murine aspergillosis: treatment with polyene and caspofungin combination and G-CSF. *J. Antimicrob. Chemother.* 56, 594–597. doi:10.1093/jac/dki252.

Sionov, E., Mendlovic, S., and Segal, E. (2006). Efficacy of amphotericin B or amphotericin B-intralipid in combination with caspofungin against experimental aspergillosis. *J. Infect.* 53, 131–139. doi:10.1016/j.jinf.2005.10.015.

Sionov, E., and Segal, E. (2003). Polyene and cytokine treatment of experimental aspergillosis. *FEMS Immunol. Med. Microbiol.* 39, 221–227.

Sionov, E., and Segal, E. (2004). Treatment of murine systemic aspergillosis with polyene-intralipid admixtures. *Med. Mycol.* 42, 73–80.

Sirivoranankul, C., Martinez, M., Chen, V., Clemons, K. V., and Stevens, D. A. (2014). Vitamin D and experimental invasive aspergillosis. *Med. Mycol.* 52, 847–852. doi:10.1093/mmy/myu048.

Sivak, O., Bartlett, K., Risovic, V., Choo, E., Marra, F., Batty, D. S., et al. (2004a). Assessing the antifungal activity and toxicity profile of amphotericin B lipid complex (ABLC; Abelcet) in combination with caspofungin in experimental systemic aspergillosis. *J. Pharm. Sci.* 93, 1382–1389. doi:10.1002/jps.20080.

Sivak, O., Bartlett, K., and Wasan, K. M. (2004b). Heat-treated Fungizone retains amphotericin B antifungal activity without renal toxicity in rats infected with Aspergillus fumigatus. *Pharm. Res.* 21, 1564–1566.

Slesiona, S., Gressler, M., Mihlan, M., Zaehle, C., Schaller, M., Barz, D., et al. (2012). Persistence versus escape: Aspergillus terreus and Aspergillus fumigatus employ different strategies during interactions with macrophages. *PloS One* 7, e31223. doi:10.1371/journal.pone.0031223.

Smith, G. R. (1972). Experimental aspergillosis in mice: aspects of resistance. *J. Hyg. (Lond.)* 70, 741–754.

Smith, G. R. (1973). Effect of sub-lethal treatment with formalin on the germination of Aspergillus fumigatus spores. *J. Hyg. (Lond.)* 71, 745–753.

Smith, G. R. (1977). Aspergillus fumigatus: a possible relationship between spore size and virulence for mice. *J. Gen. Microbiol.* 102, 413–415. doi:10.1099/00221287-102-2-413.

Smith, J. M., Davies, J. E., and Holden, D. W. (1993). Construction and pathogenicity of Aspergillus fumigatus mutants that do not produce the ribotoxin restrictocin. *Mol. Microbiol.* 9, 1071–1077.

Smith, J. M., Tang, C. M., Van Noorden, S., and Holden, D. W. (1994). Virulence of Aspergillus fumigatus double mutants lacking restriction and an alkaline protease in a low-dose model of invasive pulmonary aspergillosis. *Infect. Immun.* 62, 5247–5254.

Sohrabi, N., Hassan, Z. M., Khosravi, A. R., Tebianian, M., Mahdavi, M., Tootian, Z., et al. (2010). Invasive aspergillosis promotes tumor growth and severity in a tumor-bearing mouse model. *Can. J. Microbiol.* 56, 771–776. doi:10.1139/w10-064.

Sorci, G., Giovannini, G., Riuzzi, F., Bonifazi, P., Zelante, T., Zagarella, S., et al. (2011). The danger signal S100B integrates pathogen- and danger-sensing pathways to restrain inflammation. *PLoS Pathog.* 7, e1001315. doi:10.1371/journal.ppat.1001315.

Speth, C., Hagleitner, M., Ott, H. W., Würzner, R., Lass-Flörl, C., and Rambach, G. (2013). Aspergillus fumigatus activates thrombocytes by secretion of soluble compounds. *J. Infect. Dis.* 207, 823–833. doi:10.1093/infdis/jis743.

Spreadbury, C., Holden, D., Aufauvre-Brown, A., Bainbridge, B., and Cohen, J. (1993). Detection of Aspergillus fumigatus by polymerase chain reaction. *J. Clin. Microbiol.* 31, 615–621.

Spreghini, E., Orlando, F., Santinelli, A., Pisa, E., Loretelli, C., Manso, E., et al. (2009). Anidulafungin in combination with amphotericin B against Aspergillus fumigatus. *Antimicrob. Agents Chemother.* 53, 4035–4039. doi:10.1128/AAC.00659-09.

Steele, C., Rapaka, R. R., Metz, A., Pop, S. M., Williams, D. L., Gordon, S., et al. (2005). The beta-glucan receptor dectin-1 recognizes specific morphologies of Aspergillus fumigatus. *PLoS Pathog.* 1, e42. doi:10.1371/journal.ppat.0010042.

Stein, S., Scholz, S., Schwäble, J., Sadat, M. A., Modlich, U., Schultze-Strasser, S., et al. (2013). From bench to bedside: preclinical evaluation of a self-inactivating gammaretroviral vector for the gene therapy of X-linked chronic granulomatous disease. *Hum. Gene Ther. Clin. Dev.* 24, 86–98. doi:10.1089/humc.2013.019.

Steinbach, W. J., Benjamin, D. K., Jr, Trasi, S. A., Miller, J. L., Schell, W. A., Zaas, A. K., et al. (2004). Value of an inhalational model of invasive aspergillosis. *Med. Mycol. Off. Publ. Int. Soc. Hum. Anim. Mycol.* 42, 417–425.

Steinbach, W. J., Cramer, R. A., Perfect, B. Z., Asfaw, Y. G., Sauer, T. C., Najvar, L. K., et al. (2006). Calcineurin controls growth, morphology, and pathogenicity in Aspergillus fumigatus. *Eukaryot. Cell* 5, 1091–1103. doi:10.1128/EC.00139-06.

Stephens-Romero, S. D., Mednick, A. J., and Feldmesser, M. (2005). The pathogenesis of fatal outcome in murine pulmonary aspergillosis depends on the neutrophil depletion strategy. *Infect. Immun.* 73, 114–125. doi:10.1128/IAI.73.1.114-125.2005.

Stojanovic, I., Mirkov, I., Kataranovski, M., Glamoclija, J., and Stosic-Grujicic, S. (2011). A role for macrophage migration inhibitory factor in protective immunity against Aspergillus fumigatus. *Immunobiology* 216, 1018–1027. doi:10.1016/j.imbio.2011.03.005.

Stuehler, C., Khanna, N., Bozza, S., Zelante, T., Moretti, S., Kruhm, M., et al. (2011). Cross-protective TH1 immunity against Aspergillus fumigatus and Candida albicans. *Blood* 117, 5881–5891. doi:10.1182/blood-2010-12-325084.

Sugareva, V., Härtl, A., Brock, M., Hübner, K., Rohde, M., Heinekamp, T., et al. (2006). Characterisation of the laccase-encoding gene abr2 of the dihydroxynaphthalene-like melanin gene cluster of Aspergillus fumigatus. *Arch. Microbiol.* 186, 345–355. doi:10.1007/s00203-006-0144-2.

Sugui, J. A., Losada, L., Wang, W., Varga, J., Ngamskulrungroj, P., Abu-Asab, M., et al. (2011). Identification and characterization of an Aspergillus fumigatus “supermater” pair. *mBio* 2. doi:10.1128/mBio.00234-11.

Sugui, J. A., Pardo, J., Chang, Y. C., Müllbacher, A., Zarember, K. A., Galvez, E. M., et al. (2007a). Role of laeA in the Regulation of alb1, gliP, Conidial Morphology, and Virulence in Aspergillus fumigatus. *Eukaryot. Cell* 6, 1552–1561. doi:10.1128/EC.00140-07.

Sugui, J. A., Pardo, J., Chang, Y. C., Zarember, K. A., Nardone, G., Galvez, E. M., et al. (2007b). Gliotoxin is a virulence factor of Aspergillus fumigatus: gliP deletion attenuates virulence in mice immunosuppressed with hydrocortisone. *Eukaryot. Cell* 6, 1562–1569. doi:10.1128/EC.00141-07.

Sugui, J. A., Peterson, S. W., Clark, L. P., Nardone, G., Folio, L., Riedlinger, G., et al. (2012). Aspergillus tanneri sp. nov., a new pathogen that causes invasive disease refractory to antifungal therapy. *J. Clin. Microbiol.* 50, 3309–3317. doi:10.1128/JCM.01509-12.

Sugui, J. A., Peterson, S. W., Figat, A., Hansen, B., Samson, R. A., Mellado, E., et al. (2014). Genetic relatedness versus biological compatibility between Aspergillus fumigatus and related species. *J. Clin. Microbiol.* 52, 3707–3721. doi:10.1128/JCM.01704-14.

Sugui, J. A., Vinh, D. C., Nardone, G., Shea, Y. R., Chang, Y. C., Zelazny, A. M., et al. (2010). Neosartorya udagawae (Aspergillus udagawae), an emerging agent of aspergillosis: how different is it from Aspergillus fumigatus? *J. Clin. Microbiol.* 48, 220–228. doi:10.1128/JCM.01556-09.

Sun, Z., Zhu, P., Li, L., Wan, Z., Zhao, Z., and Li, R. (2012). Adoptive immunity mediated by HLA-A*0201 restricted Asp f16 peptides-specific CD8+ T cells against Aspergillus fumigatus infection. *Eur. J. Clin. Microbiol. Infect. Dis. Off. Publ. Eur. Soc. Clin. Microbiol.* 31, 3089–3096. doi:10.1007/s10096-012-1670-2.

Sutton, P., Waring, P., and Müllbacher, A. (1996). Exacerbation of invasive aspergillosis by the immunosuppressive fungal metabolite, gliotoxin. *Immunol. Cell Biol.* 74, 318–322. doi:10.1038/icb.1996.57.

Svirshchevskaya, E. V., Shevchenko, M. A., Huet, D., Femenia, F., Latgé, J.-P., Boireau, P., et al. (2009). Susceptibility of mice to invasive aspergillosis correlates with delayed cell influx into the lungs. *Int. J. Immunogenet.* 36, 289–299. doi:10.1111/j.1744-313X.2009.00869.x.

Takasuka, T., Sayers, N. M., Anderson, M. J., Benbow, E. W., and Denning, D. W. (1999). Aspergillus fumigatus catalases: cloning of an Aspergillus nidulans catalase B homologue and evidence for at least three catalases. *FEMS Immunol. Med. Microbiol.* 23, 125–133.

Takazono, T., Izumikawa, K., Mihara, T., Kosai, K., Saijo, T., Imamura, Y., et al. (2009). Efficacy of combination antifungal therapy with intraperitoneally administered micafungin and aerosolized liposomal amphotericin B against murine invasive pulmonary aspergillosis. *Antimicrob. Agents Chemother.* 53, 3508–3510. doi:10.1128/AAC.00285-09.

Takemoto, K., Yamamoto, Y., Ueda, Y., Kanazawa, K., Yoshida, K., and Niki, Y. (2009). Comparative study on the efficacy of liposomal amphotericin B and voriconazole in a murine pulmonary aspergillosis model. *Chemotherapy* 55, 105–113. doi:10.1159/000194661.

Takemoto, K., Yamamoto, Y., Ueda, Y., Sumita, Y., Yoshida, K., and Niki, Y. (2004). Comparative studies on the efficacy of AmBisome and Fungizone in a mouse model of disseminated aspergillosis. *J. Antimicrob. Chemother.* 53, 311–317. doi:10.1093/jac/dkh055.

Takemoto, K., Yamamoto, Y., Ueda, Y., Sumita, Y., Yoshida, K., and Niki, Y. (2006). Comparative study on the efficacy of AmBisome and Fungizone in a mouse model of pulmonary aspergillosis. *J. Antimicrob. Chemother.* 57, 724–731. doi:10.1093/jac/dkl005.

Tanaka, R. J., Boon, N. J., Vrcelj, K., Nguyen, A., Vinci, C., Armstrong-James, D., et al. (2015). In silico modeling of spore inhalation reveals fungal persistence following low dose exposure. *Sci. Rep.* 5, 13958. doi:10.1038/srep13958.

Tandon, R. N., Feuillette, A. R., Mahouy, G., Badillet, G., Friedman, R. M., and Maheshwari, R. K. (1988). Interferon protects mice against an extracellular infection of Aspergillus fumigatus. *Ann. N. Y. Acad. Sci.* 544, 409–411.

Tang, C. M., Cohen, J., Krausz, T., Van Noorden, S., and Holden, D. W. (1993). The alkaline protease of Aspergillus fumigatus is not a virulence determinant in two murine models of invasive pulmonary aspergillosis. *Infect. Immun.* 61, 1650–1656.

Tanio, T., Ichise, K., Nakajima, T., and Okuda, T. (1990). In vivo efficacy of SM-8668 (Sch 39304), a new oral triazole antifungal agent. *Antimicrob. Agents Chemother.* 34, 980–984.

Tansho, S., Abe, S., Ishibashi, H., Torii, S., Otani, H., Ono, Y., et al. (2006). Efficacy of intravenous itraconazole against invasive pulmonary aspergillosis in neutropenic mice. *J. Infect. Chemother. Off. J. Jpn. Soc. Chemother.* 12, 355–362. doi:10.1007/s10156-006-0479-2.

Taylor, P. R., Leal, S. M., Sun, Y., and Pearlman, E. (2014a). Aspergillus and Fusarium corneal infections are regulated by Th17 cells and IL-17-producing neutrophils. *J. Immunol. Baltim. Md 1950* 192, 3319–3327. doi:10.4049/jimmunol.1302235.

Taylor, P. R., Roy, S., Leal, S. M., Sun, Y., Howell, S. J., Cobb, B. A., et al. (2014b). Activation of neutrophils by autocrine IL-17A-IL-17RC interactions during fungal infection is regulated by IL-6, IL-23, RORγt and dectin-2. *Nat. Immunol.* 15, 143–151. doi:10.1038/ni.2797.

te Dorsthorst, D. T. A., Verweij, P. E., Meis, J. F. G. M., and Mouton, J. W. (2005). Efficacy and pharmacodynamics of flucytosine monotherapy in a nonneutropenic murine model of invasive aspergillosis. *Antimicrob. Agents Chemother.* 49, 4220–4226. doi:10.1128/AAC.49.10.4220-4226.2005.

Templeton, S. P., Buskirk, A. D., Law, B., Green, B. J., and Beezhold, D. H. (2011). Role of germination in murine airway CD8+ T-cell responses to Aspergillus conidia. *PloS One* 6, e18777. doi:10.1371/journal.pone.0018777.

Thau, N., Monod, M., Crestani, B., Rolland, C., Tronchin, G., Latgé, J. P., et al. (1994). rodletless mutants of Aspergillus fumigatus. *Infect. Immun.* 62, 4380–4388.

Tkalcevic, J., Novelli, M., Phylactides, M., Iredale, J. P., Segal, A. W., and Roes, J. (2000). Impaired immunity and enhanced resistance to endotoxin in the absence of neutrophil elastase and cathepsin G. *Immunity* 12, 201–210.

Tolman, J. A., Wiederhold, N. P., McConville, J. T., Najvar, L. K., Bocanegra, R., Peters, J. I., et al. (2009). Inhaled voriconazole for prevention of invasive pulmonary aspergillosis. *Antimicrob. Agents Chemother.* 53, 2613–2615. doi:10.1128/AAC.01657-08.

Torosantucci, A., Bromuro, C., Chiani, P., De Bernardis, F., Berti, F., Galli, C., et al. (2005). A novel glyco-conjugate vaccine against fungal pathogens. *J. Exp. Med.* 202, 597–606. doi:10.1084/jem.20050749.

Troke, P. F., Andrews, R. J., Marriott, M. S., and Richardson, K. (1987). Efficacy of fluconazole (UK-49,858) against experimental aspergillosis and cryptococcosis in mice. *J. Antimicrob. Chemother.* 19, 663–670.

Tsitsigiannis, D. I., Bok, J.-W., Andes, D., Nielsen, K. F., Frisvad, J. C., and Keller, N. P. (2005). Aspergillus Cyclooxygenase-Like Enzymes Are Associated with Prostaglandin Production and Virulence. *Infect. Immun.* 73, 4548–4559. doi:10.1128/IAI.73.8.4548-4559.2005.

Tsuchimori, N., Hayashi, R., Kitamoto, N., Asai, K., Kitazaki, T., Iizawa, Y., et al. (2002). In vitro and in vivo antifungal activities of TAK-456, a novel oral triazole with a broad antifungal spectrum. *Antimicrob. Agents Chemother.* 46, 1388–1393.

Turner, K. J., Hackshaw, R., Papadimitriou, J., and Perrott, J. (1976). The pathogenesis of experimental pulmonary aspergillosis in normal and cortisone-treated rats. *J. Pathol.* 118, 65–73. doi:10.1002/path.1711180202.

Turner, K. J., Hackshaw, R., Papadimitriou, J., Wetherall, J. D., and Perrott, J. (1975a). Experimental aspergillosis in rats infected via intraperitoneal and subcutaneous routes. *Immunology* 29, 55–66.

Turner, K. J., Papadimitriou, J., Hackshaw, R., and Wetherall, J. D. (1975b). Experimental aspergillosis in normal rats infected intravenously. *J. Reticuloendothel. Soc.* 17, 300–312.

Ullmann, A. J., Krammes, E., Sommer, S., Buschmann, I., Jahn-Muehl, B., Cacciapuoti, A., et al. (2007). Efficacy of posaconazole and amphotericin B in experimental invasive pulmonary aspergillosis in dexamethasone immunosuppressed rats. *J. Antimicrob. Chemother.* 60, 1080–1084. doi:10.1093/jac/dkm328.

Vaknin, Y., Shadkchan, Y., Levdansky, E., Morozov, M., Romano, J., and Osherov, N. (2014). The three Aspergillus fumigatus CFEM-domain GPI-anchored proteins (CfmA-C) affect cell-wall stability but do not play a role in fungal virulence. *Fungal Genet. Biol. FG B* 63, 55–64. doi:10.1016/j.fgb.2013.12.005.

Valiante, V., Heinekamp, T., Jain, R., Härtl, A., and Brakhage, A. A. (2008). The mitogen-activated protein kinase MpkA of Aspergillus fumigatus regulates cell wall signaling and oxidative stress response. *Fungal Genet. Biol. FG B* 45, 618–627. doi:10.1016/j.fgb.2007.09.006.

Vallon-Eberhard, A., Makovitzki, A., Beauvais, A., Latgé, J.-P., Jung, S., and Shai, Y. (2008). Efficient clearance of Aspergillus fumigatus in murine lungs by an ultrashort antimicrobial lipopeptide, palmitoyl-lys-ala-DAla-lys. *Antimicrob. Agents Chemother.* 52, 3118–3126. doi:10.1128/AAC.00526-08.

Vallor, A. C., Kirkpatrick, W. R., Najvar, L. K., Bocanegra, R., Kinney, M. C., Fothergill, A. W., et al. (2008). Assessment of Aspergillus fumigatus burden in pulmonary tissue of guinea pigs by quantitative PCR, galactomannan enzyme immunoassay, and quantitative culture. *Antimicrob. Agents Chemother.* 52, 2593–2598. doi:10.1128/AAC.00276-08.

Van Cutsem, J., Meulemans, L., Van Gerven, F., and Stynen, D. (1990). Detection of circulating galactomannan by Pastorex Aspergillus in experimental invasive aspergillosis. *Mycoses* 33, 61–69.

Van Cutsem, J., Van Gerven, F., and Janssen, P. A. (1987). Activity of orally, topically, and parenterally administered itraconazole in the treatment of superficial and deep mycoses: animal models. *Rev. Infect. Dis.* 9 Suppl 1, S15-32.

Van Cutsem, J., Van Gerven, F., and Janssen, P. A. (1989). Oral and parenteral therapy with saperconazole (R 66905) of invasive aspergillosis in normal and immunocompromised animals. *Antimicrob. Agents Chemother.* 33, 2063–2068.

Van Cutsem, J., Van Gerven, F., Van de Ven, M. A., Borgers, M., and Janssen, P. A. (1984). Itraconazole, a new triazole that is orally active in aspergillosis. *Antimicrob. Agents Chemother.* 26, 527–534.

van de Sande, W. W. J., Mathot, R. A. A., ten Kate, M. T., van Vianen, W., Tavakol, M., Rijnders, B. J. A., et al. (2009). Combination therapy of advanced invasive pulmonary aspergillosis in transiently neutropenic rats using human pharmacokinetic equivalent doses of voriconazole and anidulafungin. *Antimicrob. Agents Chemother.* 53, 2005–2013. doi:10.1128/AAC.01556-08.

van de Sande, W. W. J., van Vianen, W., ten Kate, M. T., Vissers, J., Laurijsens, J., Tavakol, M., et al. (2008). Caspofungin prolongs survival of transiently neutropenic rats with advanced-stage invasive pulmonary aspergillosis. *Antimicrob. Agents Chemother.* 52, 1345–1350. doi:10.1128/AAC.00536-07.

Van Epps, H. L., Feldmesser, M., and Pamer, E. G. (2003). Voriconazole inhibits fungal growth without impairing antigen presentation or T-cell activation. *Antimicrob. Agents Chemother.* 47, 1818–1823.

Van Etten, E. W., Stearne-Cullen, L. E., ten Kate, M., and Bakker-Woudenberg, I. A. (2000). Efficacy of liposomal amphotericin B with prolonged circulation in blood in treatment of severe pulmonary aspergillosis in leukopenic rats. *Antimicrob. Agents Chemother.* 44, 540–545.

van Vianen, W., de Marie, S., ten Kate, M. T., Mathot, R. A. A., and Bakker-Woudenberg, I. A. J. M. (2006). Caspofungin: antifungal activity in vitro, pharmacokinetics, and effects on fungal load and animal survival in neutropenic rats with invasive pulmonary aspergillosis. *J. Antimicrob. Chemother.* 57, 732–740. doi:10.1093/jac/dkl015.

Vargas-Muñiz, J. M., Renshaw, H., Richards, A. D., Lamoth, F., Soderblom, E. J., Moseley, M. A., et al. (2015). The Aspergillus fumigatus septins play pleiotropic roles in septation, conidiation, and cell wall stress, but are dispensable for virulence. *Fungal Genet. Biol. FG B* 81, 41–51. doi:10.1016/j.fgb.2015.05.014.

Vecchiarelli, A., Mazzolla, R., Farinelli, S., Cassone, A., and Bistoni, F. (1988). Immunomodulation by Candida albicans: crucial role of organ colonization and chronic infection with an attenuated agerminative strain of C. albicans for establishment of anti-infectious protection. *J. Gen. Microbiol.* 134, 2583–2592. doi:10.1099/00221287-134-9-2583.

Verweij, P. E., Oakley, K. L., Morrissey, J., Morrissey, G., and Denning, D. W. (1998). Efficacy of LY303366 against amphotericin B-susceptible and -resistant Aspergillus fumigatus in a murine model of invasive aspergillosis. *Antimicrob. Agents Chemother.* 42, 873–878.

Verweij, P. E., Te Dorsthorst, D. T. A., Janssen, W. H. P., Meis, J. F. G. M., and Mouton, J. W. (2008). In Vitro Activities at pH 5.0 and pH 7.0 and In Vivo Efficacy of Flucytosine against Aspergillus fumigatus. *Antimicrob. Agents Chemother.* 52, 4483–4485. doi:10.1128/AAC.00491-08.

Verwer, P. E. B., ten Kate, M. T., Falcone, F. H., Morroll, S., Verbrugh, H. A., Bakker-Woudenberg, I. A. J. M., et al. (2013). Evidence supporting a role for mammalian chitinases in efficacy of caspofungin against experimental aspergillosis in immunocompromised rats. *PloS One* 8, e75848. doi:10.1371/journal.pone.0075848.

Vethanayagam, R. R., Almyroudis, N. G., Grimm, M. J., Lewandowski, D. C., Pham, C. T. N., Blackwell, T. S., et al. (2011). Role of NADPH oxidase versus neutrophil proteases in antimicrobial host defense. *PloS One* 6, e28149. doi:10.1371/journal.pone.0028149.

Wagener, J., Echtenacher, B., Rohde, M., Kotz, A., Krappmann, S., Heesemann, J., et al. (2008). The putative alpha-1,2-mannosyltransferase AfMnt1 of the opportunistic fungal pathogen Aspergillus fumigatus is required for cell wall stability and full virulence. *Eukaryot. Cell* 7, 1661–1673. doi:10.1128/EC.00221-08.

Waldorf, A. R., and Diamond, R. D. (1985). Neutrophil chemotactic responses induced by fresh and swollen Rhizopus oryzae spores and Aspergillus fumigatus conidia. *Infect. Immun.* 48, 458–463.

Waldorf, A. R., Levitz, S. M., and Diamond, R. D. (1984). In vivo bronchoalveolar macrophage defense against Rhizopus oryzae and Aspergillus fumigatus. *J. Infect. Dis.* 150, 752–760.

Wallace, T. L., Paetznick, V., Cossum, P. A., Lopez-Berestein, G., Rex, J. H., and Anaissie, E. (1997). Activity of liposomal nystatin against disseminated Aspergillus fumigatus infection in neutropenic mice. *Antimicrob. Agents Chemother.* 41, 2238–2243.

Walzl, H. L., Ackerbauer, H., Meingassner, J. G., and Mieth, H. (1987). Histopathology of organ lesions in mice after an intravenous or intratracheal or intrarenal infection with aspergillus fumigatus. *Mykosen* 30, 10–18.

Wang, C., Zhan, P., Wang, L., Zeng, R., Shen, Y., Lv, G., et al. (2014a). The application of laser microdissection in molecular detection and identification of aspergillus fumigatus from murine model of acute invasive pulmonary aspergillosis. *Mycopathologia* 178, 53–61. doi:10.1007/s11046-014-9777-x.

Wang, D.-N., Toyotome, T., Muraosa, Y., Watanabe, A., Wuren, T., Bunsupa, S., et al. (2014b). GliA in Aspergillus fumigatus is required for its tolerance to gliotoxin and affects the amount of extracellular and intracellular gliotoxin. *Med. Mycol.* 52, 506–518. doi:10.1093/mmy/myu007.

Wang, J., Zhou, H., Lu, H., Du, T., Luo, Y., Wilson, I. B. H., et al. (2015). Kexin-like endoprotease KexB is required for N-glycan processing, morphogenesis and virulence in Aspergillus fumigatus. *Fungal Genet. Biol. FG B* 76, 57–69. doi:10.1016/j.fgb.2015.02.006.

Wang, Y., Chen, L., Liu, X., Cheng, D., Liu, G., Liu, Y., et al. (2013). Detection of Aspergillus fumigatus pulmonary fungal infections in mice with (99m)Tc-labeled MORF oligomers targeting ribosomal RNA. *Nucl. Med. Biol.* 40, 89–96. doi:10.1016/j.nucmedbio.2012.10.001.

Warn, P. A., Morrissey, G., Morrissey, J., and Denning, D. W. (2003). Activity of micafungin (FK463) against an itraconazole-resistant strain of Aspergillus fumigatus and a strain of Aspergillus terreus demonstrating in vivo resistance to amphotericin B. *J. Antimicrob. Chemother.* 51, 913–919. doi:10.1093/jac/dkg185.

Warn, P. A., Sharp, A., Morrissey, G., and Denning, D. W. (2010). Activity of aminocandin (IP960; HMR3270) compared with amphotericin B, itraconazole, caspofungin and micafungin in neutropenic murine models of disseminated infection caused by itraconazole-susceptible and -resistant strains of Aspergillus fumigatus. *Int. J. Antimicrob. Agents* 35, 146–151. doi:10.1016/j.ijantimicag.2009.09.029.

Warn, P. A., Sharp, A., Mosquera, J., Spickermann, J., Schmitt-Hoffmann, A., Heep, M., et al. (2006). Comparative in vivo activity of BAL4815, the active component of the prodrug BAL8557, in a neutropenic murine model of disseminated Aspergillus flavus. *J. Antimicrob. Chemother.* 58, 1198–1207. doi:10.1093/jac/dkl396.

Wartenberg, D., Lapp, K., Jacobsen, I. D., Dahse, H.-M., Kniemeyer, O., Heinekamp, T., et al. (2011). Secretome analysis of Aspergillus fumigatus reveals Asp-hemolysin as a major secreted protein. *Int. J. Med. Microbiol. IJMM* 301, 602–611. doi:10.1016/j.ijmm.2011.04.016.

Wasan, E. K., Bartlett, K., Gershkovich, P., Sivak, O., Banno, B., Wong, Z., et al. (2009). Development and characterization of oral lipid-based amphotericin B formulations with enhanced drug solubility, stability and antifungal activity in rats infected with Aspergillus fumigatus or Candida albicans. *Int. J. Pharm.* 372, 76–84. doi:10.1016/j.ijpharm.2009.01.003.

Wasan, K. M., Sivak, O., Rosland, M., Risovic, V., and Bartlett, K. (2007). Assessing the antifungal activity, pharmacokinetics, and tissue distribution of amphotericin B following the administration of Abelcet and AmBisome in combination with caspofungin to rats infected with Aspergillus fumigatus. *J. Pharm. Sci.* 96, 1737–1747. doi:10.1002/jps.20801.

Werner, J. L., Gessner, M. A., Lilly, L. M., Nelson, M. P., Metz, A. E., Horn, D., et al. (2011). Neutrophils produce interleukin 17A (IL-17A) in a dectin-1- and IL-23-dependent manner during invasive fungal infection. *Infect. Immun.* 79, 3966–3977. doi:10.1128/IAI.05493-11.

Werner, J. L., Metz, A. E., Horn, D., Schoeb, T. R., Hewitt, M. M., Schwiebert, L. M., et al. (2009). Requisite role for the dectin-1 beta-glucan receptor in pulmonary defense against Aspergillus fumigatus. *J. Immunol. Baltim. Md 1950* 182, 4938–4946. doi:10.4049/jimmunol.0804250.

Wharton, R. E., Stefanov, E. K., King, R. G., and Kearney, J. F. (2015). Antibodies generated against Streptococci protect in a mouse model of disseminated aspergillosis. *J. Immunol. Baltim. Md 1950* 194, 4387–4396. doi:10.4049/jimmunol.1401940.

White, L. O. (1977). Germination of Aspergillus fumigatus conidia in the lungs of normal and cortisone-treated mice. *Sabouraudia* 15, 37–41.

White, P. L., Wiederhold, N. P., Loeffler, J., Najvar, L. K., Melchers, W., Herrera, M., et al. (2016). Comparison of Nonculture Blood-Based Tests for Diagnosing Invasive Aspergillosis in an Animal Model. *J. Clin. Microbiol.* 54, 960–966. doi:10.1128/JCM.03233-15.

Wiederhold, N. P., Kontoyiannis, D. P., Chi, J., Prince, R. A., Tam, V. H., and Lewis, R. E. (2004). Pharmacodynamics of caspofungin in a murine model of invasive pulmonary aspergillosis: evidence of concentration-dependent activity. *J. Infect. Dis.* 190, 1464–1471. doi:10.1086/424465.

Wiederhold, N. P., Najvar, L. K., Bocanegra, R., Kirkpatrick, W. R., Patterson, T. F., and Thornton, C. R. (2013). Interlaboratory and interstudy reproducibility of a novel lateral-flow device and influence of antifungal therapy on detection of invasive pulmonary aspergillosis. *J. Clin. Microbiol.* 51, 459–465. doi:10.1128/JCM.02142-12.

Wiederhold, N. P., Najvar, L. K., Matsumoto, S., Bocanegra, R. A., Herrera, M. L., Wickes, B. L., et al. (2015). Efficacy of the investigational echinocandin ASP9726 in a guinea pig model of invasive pulmonary aspergillosis. *Antimicrob. Agents Chemother.* 59, 2875–2881. doi:10.1128/AAC.04857-14.

Wiederhold, N. P., Najvar, L. K., Vallor, A. C., Kirkpatrick, W. R., Bocanegra, R., Molina, D., et al. (2008). Assessment of serum (1->3)-beta-D-glucan concentration as a measure of disease burden in a murine model of invasive pulmonary aspergillosis. *Antimicrob. Agents Chemother.* 52, 1176–1178. doi:10.1128/AAC.01425-07.

Wiederhold, N. P., Tam, V. H., Chi, J., Prince, R. A., Kontoyiannis, D. P., and Lewis, R. E. (2006). Pharmacodynamic activity of amphotericin B deoxycholate is associated with peak plasma concentrations in a neutropenic murine model of invasive pulmonary aspergillosis. *Antimicrob. Agents Chemother.* 50, 469–473. doi:10.1128/AAC.50.2.469-473.2006.

Wiederhold, N. P., Thornton, C. R., Najvar, L. K., Kirkpatrick, W. R., Bocanegra, R., and Patterson, T. F. (2009). Comparison of lateral flow technology and galactomannan and (1->3)-beta-D-glucan assays for detection of invasive pulmonary aspergillosis. *Clin. Vaccine Immunol. CVI* 16, 1844–1846. doi:10.1128/CVI.00268-09.

Willger, S. D., Cornish, E. J., Chung, D., Fleming, B. A., Lehmann, M. M., Puttikamonkul, S., et al. (2012). Dsc orthologs are required for hypoxia adaptation, triazole drug responses, and fungal virulence in Aspergillus fumigatus. *Eukaryot. Cell* 11, 1557–1567. doi:10.1128/EC.00252-12.

Willger, S. D., Puttikamonkul, S., Kim, K.-H., Burritt, J. B., Grahl, N., Metzler, L. J., et al. (2008). A sterol-regulatory element binding protein is required for cell polarity, hypoxia adaptation, azole drug resistance, and virulence in Aspergillus fumigatus. *PLoS Pathog.* 4, e1000200. doi:10.1371/journal.ppat.1000200.

Williams, D. M., Weiner, M. H., and Drutz, D. J. (1981). Immunologic studies of disseminated infection with Aspergillus fumigatus in the nude mouse. *J. Infect. Dis.* 143, 726–733.

Winkelströter, L. K., Bom, V. L. P., de Castro, P. A., Ramalho, L. N. Z., Goldman, M. H. S., Brown, N. A., et al. (2015). High osmolarity glycerol response PtcB phosphatase is important for Aspergillus fumigatus virulence. *Mol. Microbiol.* 96, 42–54. doi:10.1111/mmi.12919.

Wong, B., Brauer, K. L., Tsai, R. R., and Jayasimhulu, K. (1989). Increased amounts of the Aspergillus metabolite D-mannitol in tissue and serum of rats with experimental aspergillosis. *J. Infect. Dis.* 160, 95–103.

Wong Sak Hoi, J., Lamarre, C., Beau, R., Meneau, I., Berepiki, A., Barre, A., et al. (2011). A novel family of dehydrin-like proteins is involved in stress response in the human fungal pathogen Aspergillus fumigatus. *Mol. Biol. Cell* 22, 1896–1906. doi:10.1091/mbc.E10-11-0914.

Xu, P., Qu, J.-M., Xu, J.-F., Zhang, J., Jiang, H.-N., and Zhang, H.-J. (2009). NAC is associated with additional alleviation of lung injury induced by invasive pulmonary aspergillosis in a neutropenic model. *Acta Pharmacol. Sin.* 30, 980–986. doi:10.1038/aps.2009.83.

Xu, Q., Zhao, G., Lin, J., Wang, Q., Hu, L., and Jiang, Z. (2015). Role of Dectin-1 in the innate immune response of rat corneal epithelial cells to Aspergillus fumigatus. *BMC Ophthalmol.* 15, 126. doi:10.1186/s12886-015-0112-1.

Yamada, H., Tsuda, T., Watanabe, T., Ohashi, M., Murakami, K., and Mochizuki, H. (1993). In vitro and in vivo antifungal activities of D0870, a new triazole agent. *Antimicrob. Agents Chemother.* 37, 2412–2417.

Yamakami, Y., Hashimoto, A., Tokimatsu, I., and Nasu, M. (1996). PCR detection of DNA specific for Aspergillus species in serum of patients with invasive aspergillosis. *J. Clin. Microbiol.* 34, 2464–2468.

Yan, Y., Zhao, Z., Wan, H., Wu, R., Fang, J., and Liu, H. (2014). A novel fungus concentration-dependent rat model for acute invasive fungal rhinosinusitis: an experimental study. *BMC Infect. Dis.* 14, 3856. doi:10.1186/s12879-014-0713-y.

Yang, J., Liu, W., Lu, Q., Wan, Z., Wang, X., and Li, R. (2009a). Different expression of dectin-1 and Toll-like receptor 2 in the lungs of different immune status mice infected with Aspergillus fumigatus. *Chin. Med. J. (Engl.)* 122, 2017–2021.

Yang, J., Lu, Q., Liu, W., Wan, Z., Wang, X., and Li, R. (2010). Cyclophosphamide reduces dectin-1 expression in the lungs of naive and Aspergillus fumigatus-infected mice. *Med. Mycol.* 48, 303–309. doi:10.1080/13693780903136887.

Yang, Z., Kontoyiannis, D. P., Wen, X., Xiong, C., Zhang, R., Albert, N. D., et al. (2009b). Gamma scintigraphy imaging of murine invasive pulmonary aspergillosis with a (111)In-labeled cyclic peptide. *Nucl. Med. Biol.* 36, 259–266. doi:10.1016/j.nucmedbio.2008.12.004.

Yasmin, S., Alcazar-Fuoli, L., Gründlinger, M., Puempel, T., Cairns, T., Blatzer, M., et al. (2012). Mevalonate governs interdependency of ergosterol and siderophore biosyntheses in the fungal pathogen Aspergillus fumigatus. *Proc. Natl. Acad. Sci. U. S. A.* 109, E497-504. doi:10.1073/pnas.1106399108.

Yonezawa, M., Sugiyama, H., Kizawa, K., Hori, R., Mitsuyama, J., Araki, H., et al. (2000). A new model of pulmonary superinfection with Aspergillus fumigatus and Pseudomonas aeruginosa in mice. *J. Infect. Chemother. Off. J. Jpn. Soc. Chemother.* 6, 155–161. doi:10.1007/s101560000005.

Yu, B., Niki, Y., and Armstrong, D. (1990). Use of immunoblotting to detect Aspergillus fumigatus antigen in sera and urines of rats with experimental invasive aspergillosis. *J. Clin. Microbiol.* 28, 1575–1579.

Zaas, A. K., Liao, G., Chien, J. W., Weinberg, C., Shore, D., Giles, S. S., et al. (2008). Plasminogen alleles influence susceptibility to invasive aspergillosis. *PLoS Genet.* 4, e1000101. doi:10.1371/journal.pgen.1000101.

Zelante, T., Bozza, S., De Luca, A., D’Angelo, C., Bonifazi, P., Moretti, S., et al. (2009). Th17 cells in the setting of Aspergillus infection and pathology. *Med. Mycol.* 47 Suppl 1, S162-169. doi:10.1080/13693780802140766.

Zelante, T., De Luca, A., Bonifazi, P., Montagnoli, C., Bozza, S., Moretti, S., et al. (2007). IL-23 and the Th17 pathway promote inflammation and impair antifungal immune resistance. *Eur. J. Immunol.* 37, 2695–2706. doi:10.1002/eji.200737409.

Zelante, T., Iannitti, R. G., De Luca, A., Arroyo, J., Blanco, N., Servillo, G., et al. (2012). Sensing of mammalian IL-17A regulates fungal adaptation and virulence. *Nat. Commun.* 3, 683. doi:10.1038/ncomms1685.

Zelante, T., Wong, A. Y. W., Ping, T. J., Chen, J., Sumatoh, H. R., Viganò, E., et al. (2015). CD103(+) Dendritic Cells Control Th17 Cell Function in the Lung. *Cell Rep.* 12, 1789–1801. doi:10.1016/j.celrep.2015.08.030.

Zhang, C., Kong, Q., Cai, Z., Liu, F., Chen, P., Song, J., et al. (2015). The newly nonsporulated characterization of an Aspergillus fumigatus isolate from an immunocompetent patient and its clinic indication. *Fungal Genet. Biol. FG B* 81, 250–260. doi:10.1016/j.fgb.2015.03.001.

Zhang, C.-R., Lin, J.-C., Xu, W.-M., Li, M., Ye, H.-S., Cui, W.-L., et al. (2013a). Interleukin-12 and interleukin-2 alone or in combination against the infection in invasive pulmonary aspergillosis mouse model. *Mycoses* 56, 117–122. doi:10.1111/j.1439-0507.2012.02220.x.

Zhang, F., An, Y., Li, Z., and Zhao, C. (2013b). A novel model of invasive fungal rhinosinusitis in rats. *Am. J. Rhinol. Allergy* 27, 361–366. doi:10.2500/ajra.2013.27.3953.

Zhang, H., Qu, J., Shao, C., Zhang, J., He, L., and Yuan, Z. (2008a). Aspergillus fumigatus conidia upregulates NOD2 protein expression both in vitro and in vivo. *Acta Pharmacol. Sin.* 29, 1202–1208. doi:10.1111/j.1745-7254.2008.00860.x.

Zhang, L., Wang, M., Li, R., and Calderone, R. (2005). Expression of Aspergillus fumigatus virulence-related genes detected in vitro and in vivo with competitive RT-PCR. *Mycopathologia* 160, 201–206. doi:10.1007/s11046-005-0141-z.

Zhang, L., Zhou, H., Ouyang, H., Li, Y., and Jin, C. (2008b). Afcwh41 is required for cell wall synthesis, conidiation, and polarity in Aspergillus fumigatus. *FEMS Microbiol. Lett.* 289, 155–165.

Zhang, M., Su, X., Sun, W.-K., Chen, F., Xu, X.-Y., and Shi, Y. (2014). Efficacy of the combination of voriconazole and caspofungin in experimental pulmonary aspergillosis by different Aspergillus species. *Mycopathologia* 177, 11–18. doi:10.1007/s11046-013-9719-z.

Zhao, J., Cheng, Y., Song, X., Wang, C., Su, G., and Liu, Z. (2015). A Comparative Treatment Study of Intravitreal Voriconazole and Liposomal Amphotericin B in an Aspergillus fumigatus Endophthalmitis Model. *Invest. Ophthalmol. Vis. Sci.* 56, 7369–7376. doi:10.1167/iovs.15-17266.

Zhao, W., Panepinto, J. C., Fortwendel, J. R., Fox, L., Oliver, B. G., Askew, D. S., et al. (2006). Deletion of the regulatory subunit of protein kinase A in Aspergillus fumigatus alters morphology, sensitivity to oxidative damage, and virulence. *Infect. Immun.* 74, 4865–4874. doi:10.1128/IAI.00565-06.

Zhao, Y., Park, S., Warn, P., Shrief, R., Harrison, E., and Perlin, D. S. (2010). Detection of Aspergillus fumigatus in a Rat Model of Invasive Pulmonary Aspergillosis by Real-Time Nucleic Acid Sequence-Based Amplification. *J. Clin. Microbiol.* 48, 1378–1383. doi:10.1128/JCM.02214-09.

Zhao, Y., and Perlin, D. S. (2013). Quantitative detection of Aspergillus spp. by real-time nucleic acid sequence-based amplification. *Methods Mol. Biol. Clifton NJ* 968, 83–92. doi:10.1007/978-1-62703-257-5_6.

Zhong, W., Yin, H., and Xie, L. (2009). Expression and potential role of major inflammatory cytokines in experimental keratomycosis. *Mol. Vis.* 15, 1303–1311.

Zhou, H., Hu, H., Zhang, L., Li, R., Ouyang, H., Ming, J., et al. (2007). O-Mannosyltransferase 1 in Aspergillus fumigatus (AfPmt1p) is crucial for cell wall integrity and conidium morphology, especially at an elevated temperature. *Eukaryot. Cell* 6, 2260–2268. doi:10.1128/EC.00261-07.

Zimmerli, S., Knecht, U., and Leib, S. L. (2007). A model of cerebral aspergillosis in non-immunosuppressed nursing rats. *Acta Neuropathol. (Berl.)* 114, 411–418. doi:10.1007/s00401-007-0255-0.
